# Macrocyclic Inhibitors Targeting the Prime Site of the Fibrinolytic Serine Protease Plasmin

**DOI:** 10.1002/cmdc.202400360

**Published:** 2024-09-30

**Authors:** Simon J. A. Wiedemeyer, Guojie Wu, Heike Lang‐Henkel, James C Whisstock, Ruby H. P. Law, Torsten Steinmetzer

**Affiliations:** ^1^ Department of Pharmacy Institute of Pharmaceutical Chemistry Philipps University Marburg Marbacher Weg 6 D-35032 Marburg Germany; ^2^ Biomedicine Discovery Institute Department of Biochemistry and Molecular Biology Monash University Melbourne 3800 Australia

**Keywords:** Plasmin, Protease inhibitor, Fibrinolysis, Enzyme kinetics, Crystal structure

## Abstract

Two series of macrocyclic inhibitors addressing the S1 pocket and the prime site of the fibrinolytic serine protease plasmin have been developed. In the first series, a P1 tranexamoyl residue was coupled to 4‐aminophenylalanine in P1’ position, which provided moderately potent inhibitors with inhibition constants around 1 μM. In the second series, a substituted biphenylalanine was incorporated as P1’ residue leading to approximately 1000‐fold stronger plasmin inhibitors, the best compounds possess subnanomolar inhibition constants. The most effective compounds already exhibit a certain selectivity as plasmin inhibitors compared to other trypsin‐like serine proteases such as trypsin, plasma kallikrein, thrombin, activated protein Ca, as well as factors XIa and Xa. For inhibitor **28** of the second series, the co‐crystal structure in complex with a Ser195Ala microplasmin mutant revealed that the P2’ residue adopts multiple conformations. Most polar contacts to plasmin and surrounding water molecules are mediated through the P1 tranexamoyl residue, whereas the bound conformation of the macrocycle is mainly stabilized by two intramolecular hydrogen bonds.

## Graphical abstract

New macrocyclic inhibitors addressing the S1 pocket and the prime site of the fibrinolytic serine protease plasmin have been developed. A strong potency was found for compounds containing a substituted biphenylalanine as P1’ residue, some of them already exhibit a certain selectivity as plasmin inhibitors. For one inhibitor, a co‐crystal structure in complex with a Ser195Ala microplasmin mutant was determined.

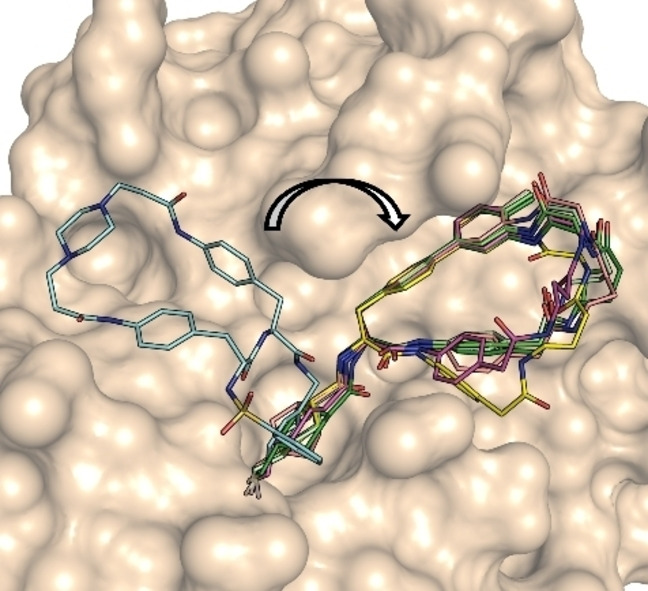

## Introduction

The trypsin‐like serine protease plasmin catalyzes the degradation of fibrin clots into soluble fragments, thereby maintaining a normal blood flow in the circulation. Intravascular plasmin is formed from its zymogen plasminogen after activation by tissue‐type plasminogen activator (tPA), whereas extravascular plasmin, which contributes to tissue remodeling and cell migration, is mainly produced by the urokinase‐type plasminogen activator (uPA), especially when bound to its receptor uPAR leading to an enhanced uPA activity.[^1^, ^2^] Under physiological conditions, the activity of plasmin is tightly controlled by endogenous inhibitors, the serpin α2‐antiplasmin and by α2‐macroglobulin. On the other hand, enhanced plasmin activities can lead to hyperfibrinolytic situations associated with life‐threatening bleeding, e. g., in cardiac surgery with cardiopulmonary bypass, transplants of liver and lung, trauma, hemorrhagic shock, and postpartum hemorrhage. Such bleeding disorders can be either treated with inhibitors of the plasmin formation, e. g., by tranexamic acid (TXA), or by direct plasmin inhibitors like aprotinin. TXA is blocking the kringle domains of plasminogen and tPA, thereby preventing an efficient binding of both proteins on fibrin surfaces and subsequent plasminogen activation. TXA has no effect on activated plasmin and its moderate affinity requires the use of relatively high TXA concentrations, which can lead to seizures, probably caused by a nonspecific binding to the GABA_A_ and glycine receptors.[^3^, ^4^] Numerous side effects, like an increased risk of myocardial infarction, heart failure, stroke, or encephalopathy have been reported for the use of the direct plasmin inhibitor aprotinin in cardiac surgery.[^5^, ^6^] As a consequence, aprotinin is no longer used in the United States to reduce perioperative bleeding. Therefore, the development of new effective and specific plasmin inhibitors is of general interest.[^1^, ^7^, ^8^]

**Figure 1:**
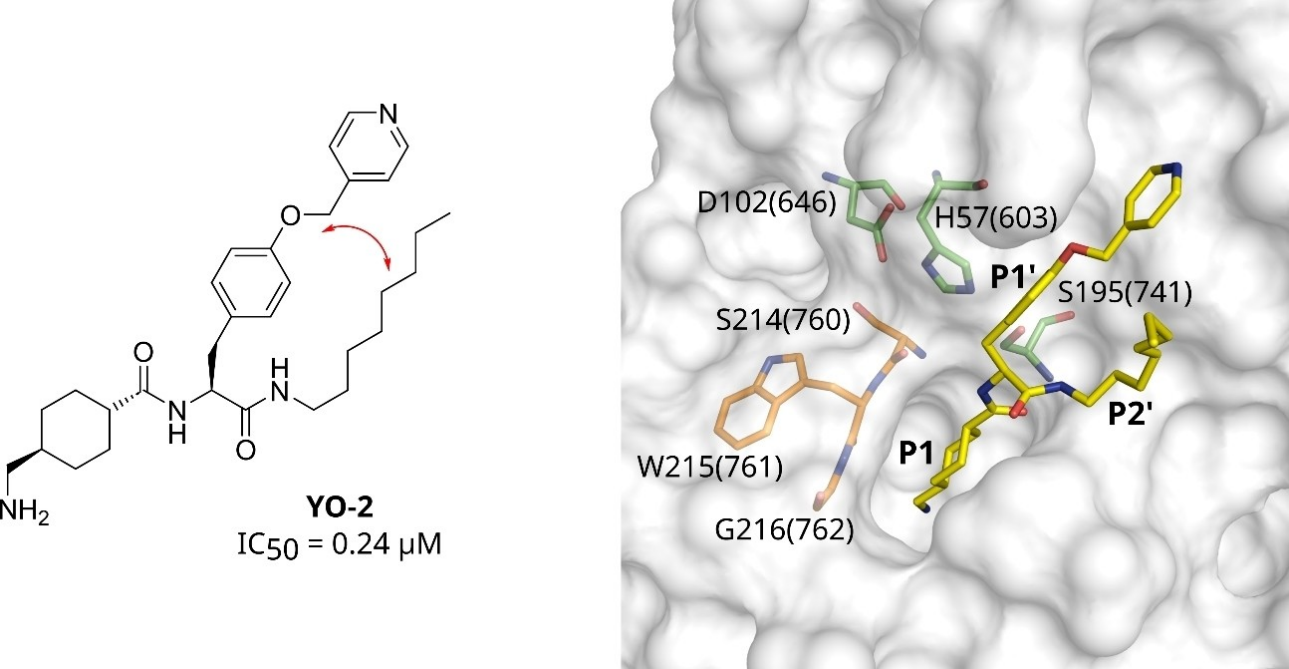
Chemical structure and crystal structure of the plasmin inhibitor YO‐2 (carbon atoms in yellow, PDB ID: 5UGD) in complex with μ‐plasmin (shown with its transparent surface in gray).^[15]^ Both arms of the Y‐shaped inhibitor are directed towards the prime site of plasmin. The spatial proximity of the P1′ side chain and the P2′ group indicated by the red arrow (left) suggested a cyclization between these residues. Residues of the catalytic triad are shown with carbon atoms in green. The plasmin segment S214(760)‐G216(762) with carbons in orange indicates the unoccupied non‐prime site. In the whole manuscript, the first plasmin residue number always corresponds to the chymotrypsinogen numbering and the second number in parenthesis to the full‐length plasminogen numbering.

The currently most effective synthetic plasmin inhibitors comprise two types of macrocyclic inhibitors, both also possessing an excellent selectivity profile. The first series consists of optimized analogs of the 14‐mer bicyclic sunflower‐trypsin inhibitor SFTI‐1,^[9]^ which inhibit plasmin with *K*_i_ values of approximately 50 pM. These inhibitors achieve their strong potency by simultaneously addressing non‐prime and prime‐site binding pockets in the active site of plasmin. The second series are substrate analog inhibitors cyclized between the side chains of a P2 amino acid in L‐configuration and P3 residue in D‐configuration. These considerably smaller inhibitors only address non‐prime binding pockets; depending on the used P1 residue, also with these compounds subnanomolar inhibitory potencies against plasmin could be achieved.[^10^, ^11^, ^12^] Several years earlier, numerous acyclic plasmin inhibitors containing a tranexamic acid residue coupled to the amino group of an alkylated tyrosine were developed, which inhibit plasmin with IC_50_ values of approximately 0.2 μM.[^13^, ^14^] Two crystal structures of these inhibitors in complex with μ‐plasmin confirmed the binding of the tranexamoyl (Txa) and tyrosine residues in the S1 and S1' pockets, respectively (Figure 1). Furthermore, the octylamide occupies the S2' area leading to a Y‐shaped overall conformation of these inhibitors.^[15]^ The structures have also shown that the S1' and S2' pockets form a open and well‐accessible binding region. (Figure 1). At the same time, Tsuda *et al*. have described first cyclic analogues of this inhibitor type, where the side chain oxygen and the carboxyl group of the P1' Tyr residue have been connected by various linker segments. The most potent compound of this series inhibits plasmin with an IC_50_ value of 3.7 μM.^[16]^

Because of our general interest in developing macrocycylic protease inhibitors not only for plasmin,[^17^, ^18^, ^19^] we speculated that the inhibitory efficacy of such inhibitors could be enhanced by a cyclization between a phenylalanine derived P1' side chain and a suitable P2' group. To test this hypothesis, two series of Txa‐derived macrocyclic plasmin inhibitors have been synthesized. Their inhibitory potency was tested with plasmin and selected trypsin‐like serine proteases. For one of the potent inhibitors of the second series, a crystal structure in complex with a Ser195Ala mutant of μ‐plasmin has been determined.

## Results

### Synthesis

**Scheme 1:**
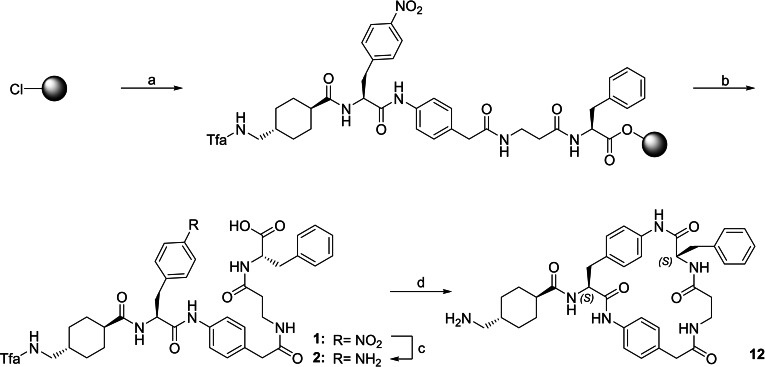
Synthesis of inhibitor 12 starting from 2‐CTC resin. a) initial resin loading using Fmoc‐Phe‐OH, followed by subsequent coupling of Fmoc‐β‐Ala‐OH, Fmoc‐4‐NH‐phenylacetic acid, Fmoc‐Phe(4‐NO_2_)‐OH, and Tfa‐tranexamic acid using a standard Fmoc protocol with 20 % piperidin in DMF for Fmoc deprotection and HATU and DIPEA for coupling (couplings were performed with 3‐fold excess of amino acids and HATU, respectively, in presence of 6 equiv. DIPEA); b) 2 % TFA in DCM; c) hydrogenation with H_2_ and Pd/C as catalyst; d) i: HATU, DIPEA in DMF, ii: 1 N NaOH in dioxane/DMSO, iii: purification by preparative HPLC.

The inhibitors were synthesized by a combination of solid phase peptide synthesis (SPPS) using a standard Fmoc strategy and solution synthesis. The preparation of the most potent inhibitor **12** of the first series is shown in Scheme 1. The linear precursor was synthesized by SPPS on 2‐chlorotrityl chloride (2‐CTC) resin. After cleavage of the peptide from the resin, the subsequent reduction of the nitro group, cyclisation, and final removal of the Tfa protecting group were performed in solution. The other inhibitors **3**–**11** of this series (Table 1) were synthesized by an identical strategy using the respective Fmoc amino acids.

**Table 1:** Inhibition of plasmin by macrocyclic Txa‐derived inhibitors containing 4‐aminophenylalanine in P1’ position (series one).

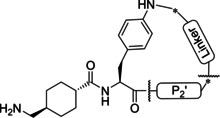			
No.	P2’	Linker	*K*_i_ (μM)
3	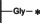	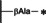	150±31
4	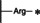	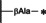	110±56
5	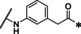	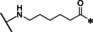	5.81±1.09
6	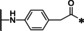	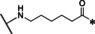	1.38±0.15
7	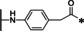	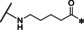	2.45±0.02
8	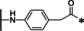	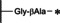	5.53±0.42
9	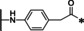	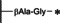	2.82±0.20
10	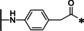	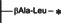	3.33±0.41
11	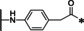	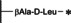	3.85±0.28
12	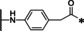	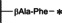	0.714±0.032

**Scheme 2:**
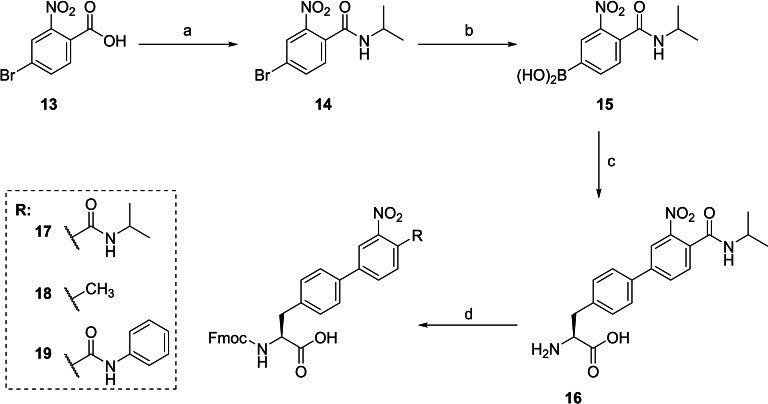
Synthesis of the Fmoc‐protected Bpa derivative 17. a) isopropylamine, HATU, DIPEA in DMF; b) B_2_pin_2_, Pd(dppf)Cl_2_, KOAc in dimethoxyethane; c) i: Boc‐Phe(4‐Br)‐OH, Pd(dppf)Cl_2_, 2 M CsCO_3_ in H_2_O, dimethoxyethane, ii: 4 M HCl in dioxane; d) TMSCl, DIPEA followed by Fmoc‐Cl and DIPEA.^[21]^ Analogues 18 and 19 were prepared by an identical strategy using 4‐methyl‐3‐nitro‐phenylboronic acid or 4‐phenylamido‐3‐nitro‐phenylboronic acid for the Suzuki coupling step.

For the inhibitors of the second series, three Fmoc protected biphenylalanine (Bpa) derivatives were prepared by Suzuki coupling, the synthesis of compound **17** is shown in Scheme 2. The respective boronic acid **15** was synthesized in two steps from bromide **13**. Initial attempts to use Fmoc‐Phe(4‐Br)‐OH for the Suzuki coupling provided negligible amounts of the required product, although this strategy was recently described for analogous Fmoc‐Bpa‐OH derivatives.^[20]^ In contrast, the Suzuki coupling with Boc‐Phe(4‐Br)‐OH and subsequent removal of the Boc group provided intermediate **16**, which was finally converted to the Fmoc protected amino acid **17**. As described in the supporting information, the analogous Bpa derivative **18** was prepared from commercially available 4‐methyl‐3‐nitro‐phenylboronic acid and the 4‐phenylamido‐3‐nitro‐phenylboronic acid required for the synthesis of compound **19** was obtained as described for compound **15** using aniline for coupling with benzoic acid **13**.

**Scheme 3:**
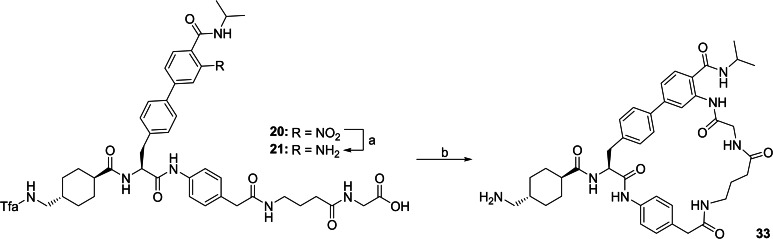
Synthesis of inhibitor 33. Intermediate 20 was prepared by standard Fmoc SPPS on 2‐CTC‐resin as described above in Scheme 1: The 2‐CTC‐resin was loaded with Fmoc‐Gly‐OH, followed by subsequent coupling of Fmoc‐Gaba‐OH, Fmoc‐4‐aminophenylacetic acid, Fmoc‐Bpa(4‐Ipa,3‐NO_2_)‐OH (17), and Tfa‐Txa‐OH. Mild acidic cleavage from resin provided compound 20. a) hydrogenation with H_2_ and Pd/C as catalyst; b) i: HATU, DIPEA in DMF, ii: 1 N NaOH in dioxane/DMSO, iii: purification by preparative HPLC.

The Fmoc‐protected Bpa derivatives were used to synthesize the inhibitors shown in Table 2 according to an identical strategy as described above. The preparation of the most potent inhibitor **33** is given in Scheme 3. Its acyclic precursor **20** was synthesized by Fmoc SPPS, followed by mild acidic cleavage from resin. Reduction of the nitro group, subsequent cyclization, and final removal of the Tfa protection provided inhibitor **33**.

**Table 2:** Inhibition of plasmin by series two macrocyclic Txa‐derived inhibitors containing substituted Bpa derivatives in P1’ position (n indicates the number of main chain atoms in the linker segment). Additional *k*_on_ and *k*_off_ values are provided for slow‐binding inhibitors with *K*_i_ values ≤10 nM.

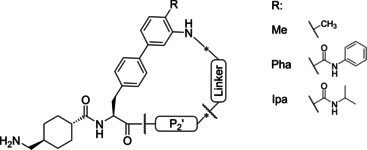							
No.	R	P2’	Linker	n	*K*_i_ (nM)	*k*_on_ (M^−1^⋅s^−1^)	*k*_off_ (s^−1^)
22	Me	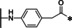	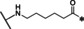	7	42.1±4.4	–	–
23	Me	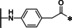	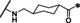	7	109±4.0	–	–
24	Me	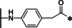	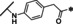	7	10.5±1.4	1.12 ⋅ 10^5^±1.06 ⋅ 10^4^	1.17 ⋅ 10^−3^±1.26 ⋅ 10^−4^
25	Me	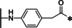	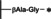	7	33.8±1.4	–	–
26	Me	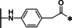	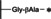	7	33.1±3.2	–	–
27	Me	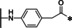	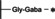	8	17.0±0.6	–	–
28	Me	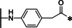	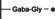	8	0.832±0.044	2.79 ⋅ 10^6^±1.47 ⋅ 10^4^	2.28 ⋅ 10^−3^±1.22 ⋅ 10^−4^
29	Me	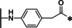	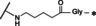	9	62.6±2.3	–	–
30	Me	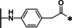	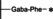	8	2.08±0.05	9.60 ⋅ 10^5^ ±3.75 ⋅ 10^4^	1.99 ⋅ 10^−3^±9.94 ⋅ 10^−5^
31	Me	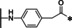	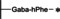	8	0.831±0.154	1.07 ⋅ 10^6^±7.84 ⋅ 10^4^	8.90 ⋅ 10^−4^±1.64 ⋅ 10^−4^
32	Pha	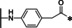	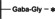	8	1.46±0.07	1.79 ⋅ 10^6^±4.56 ⋅ 10^4^	2.60 ⋅ 10^−3^±1.23 ⋅ 10^−4^
33	Ipa	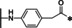	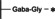	8	0.524±0.011	2.76 ⋅ 10^6^±3.66 ⋅ 10^4^	1.44 ⋅ 10^−3^±8.55 ⋅ 10^−6^
34	Me	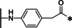	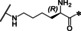	7	36.5±0.7	–	–
35	Me	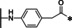	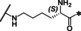	7	29.8±1.6	–	–
36	Me	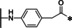	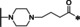	8	112±6	–	–
37	Me	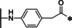	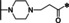	7	13.0±0.2	–	–
38	Me	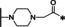	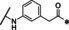	6	35.7±1.1	–	–
39	Me	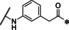	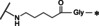	9	26.9±0.5	–	–
40	Me	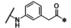	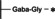	8	9.47±0.75	2.53 ⋅ 10^5^±2.22 ⋅ 10^4^	2.39 ⋅ 10^−3^±9.91 ⋅ 10^−5^
41	Me	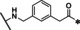	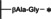	7	20.6±0.5	–	–
42	Me	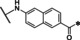	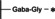	8	284±24	–	–
43	Me	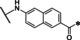	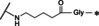	9	5.14±0.27	3.81 ⋅ 10^5^±4.04 ⋅ 10^4^	1.96 ⋅ 10^−3^±1.86 ⋅ 10^−4^
44	Me	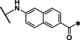	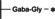	8	10.3±0.2	–	–
45	Me	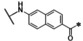	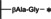	7	2.53±0.23	1.57 ⋅ 10^6^±1.00 ⋅ 10^4^	3.98 ⋅ 10^−3^±3.56 ⋅ 10^4^

### Enzyme Kinetics

All inhibitors prepared in this study act as reversible competitive plasmin inhibitors, their *K*_i_ values were calculated by fitting the steady‐state velocities as function of the inhibitor concentrations using equation 1. 4‐aminophenylalanine (Phe(4‐NH_2_)) was incorporated as P1’ residue in the first inhibitor series, which enabled a macrocyclization via amide bond formation from its side chain amino group using various linker segments and P2’ residues (Table 1). A weak plasmin inhibition of 150 μM was observed for inhibitor **3** containing Gly in P2’ position and β‐alanine as linker. The improved potency of SFTI‐1 analogues with basic P2’ residues^[9]^ stimulated the incorporation of Arg in that position leading to inhibitor **4** with a slightly stronger inhibitory potency. A significant improvement could be achieved when using 3‐ or 4‐aminophenylacetic acid as P2’ group in combination with elongated linker residues (**5**‐**6**). The enhanced potency of inhibitor **6** (*K*_i_=1.38 μM) encouraged the synthesis of additional 4‐aminophenylacetic acid derived inhibitors like compounds **7**–**12**. The truncation of the seven atoms long 6‐aminocaproyl linker by one methylene group to an 5‐aminovaleroyl residue resulted in a twofold reduced potency (**7**). Furthermore, no benefit was achieved after replacing the flexible aminocaproyl linker with the more rigid Gly‐β‐Ala or β‐Ala‐Gly linker segments, also possessing seven main chain atoms. However, inhibitor **9** is a twofold stronger plasmin inhibitor compared to its analogue **8**. Further replacement of the Gly residue in inhibitor **9** with standard amino acids resulted in inhibitor **12**, the first analogue of this series possessing a submicromolar plasmin inhibition (*K*_i_=0.71 μM) (Table 1).

The design of a second inhibitor series was stimulated by patents from Bayer[^22^, ^23^, ^24^] disclosing acyclic Txa‐derived inhibitors containing substituted Bpa residues in P1’ position, selected structures are shown in recent reviews.[^7^, ^8^] However, these compounds possess limited selectivity and act as dual inhibitors of both the blood coagulation factor XIa and plasmin. Nevertheless, we tried to incorporate substituted Bpa residues suitable for macrocyclization in our second series (Table 2). Most compounds have been prepared with a Bpa(4’‐Me, 3’‐NO_2_) residue in P1’ position, whereat the side chain nitro group was reduced to an amino group suitable for cyclization. Compared with the inhibitors described above, a considerably improved plasmin affinity was already found for the first compound **22** (*K*_i_=42 nM) containing 4‐aminophenylacetic acid in P2’ position and a flexible 6‐aminocaproic acid as linker segment, both known from the first series. A further 4‐fold enhanced potency was determined for compound **24** (*K*_i_=10.5 nM), containing a second and more rigid 4‐aminophenylacetyl residue as linker group, whereas only a weak potency >100 nM was obtained for the P2′ Txa‐derived inhibitor **23**. Variations of the linker length using aliphatic amino acids revealed a clear preference for an eight atoms long linker consisting of Gaba and Gly, where Gaba was coupled to the P2’ residue. This provided inhibitor **28** (*K*_i_=0.83 nM), the first subnanomolar inhibitor of this series. The tested compounds with a shorter seven atoms long linker (**25**‐**26**) or longer nine atoms long linker (**29**), as well as the reverse incorporation of Gaba and Gly in case of inhibitor **27**, exhibit a considerably reduced potency. The replacement of Gly in the linker of inhibitor **28** by Phe resulted in a slight loss of plasmin affinity (**30**), whereas the incorporation of homoPhe in this position provided an equipotent inhibitor (**31**). Two further inhibitors with differently substituted Bpa residues were prepared. No benefit was found for the phenylamide analog **32**, whereas an enhanced plasmin inhibition could be achieved when the methyl substituent was replaced by an isopropylamide group. Compound **33** with a *K*_i_ value of 0.52 nM is the most potent plasmin inhibitor of this second series.

Notably, for inhibitors with *K*_i_ values ≤10 nM, a pronounced slow‐binding behavior^[25]^ was observed, which leads to nonlinear progress curves at the beginning of the measurement followed by a linear part, when the steady‐state is established. For these inhibitors the steady‐state velocities were calculated by fitting the progress curves to equation 2 providing an additional apparent first‐order rate constant *k*_obs_ for each curve. The steady‐state rates were used for *K*_i_ determination with equation 1 and the *k*_obs_ values for the calculation of the association rate constants *k*_on_ according to equation 3. The dissociation rate constant *k*_off_ was calculated from the known *K*_i_ and *k*_on_ values using equation 4. The enzyme kinetic evaluation of slow‐binding inhibitors of plasmin has already been described in detail in our two previous publications.[^11^, ^12^]

Compounds **34**–**38** have been prepared to enhance the water‐solubility of this inhibitor type by introducing an additional protonatable group. In inhibitors **34** and **35** d‐ or l‐lysine was incorporated via their side chain into the macrocycle. The potencies of these compounds are in a similar range as found for compound **22** containing the ϵ‐aminocaproyl group possessing the same seven atoms long linker length, but are significantly reduced when compared with inhibitor **33**. Compounds **36**–**38** contain a protonatable alkylated piperazine moiety and only differ in their linker length. The *K*_i_ values show a preference for inhibitor **37** (*K*_i_=13 nM) with a seven atoms long linker group.

Additional compounds containing different P2’ groups have been synthesized (**39**–**45**). The data reveal a preference of a 3‐amino‐phenylacetyl residue in P2’ position when compared with the elongated 3‐aminomethyl‐phenylacetyl group. A relatively potent plasmin inhibition was also found for compound **45** (*K*_i_=2.3 nM) containing a rigid 6 amido‐2‐naphthoyl group in P2’ position combined with an β‐Ala‐Gly linker. However, all of these compounds are less potent than the subnanomolar inhibitors **28**, **31**, or **33**.

**Figure 2:**
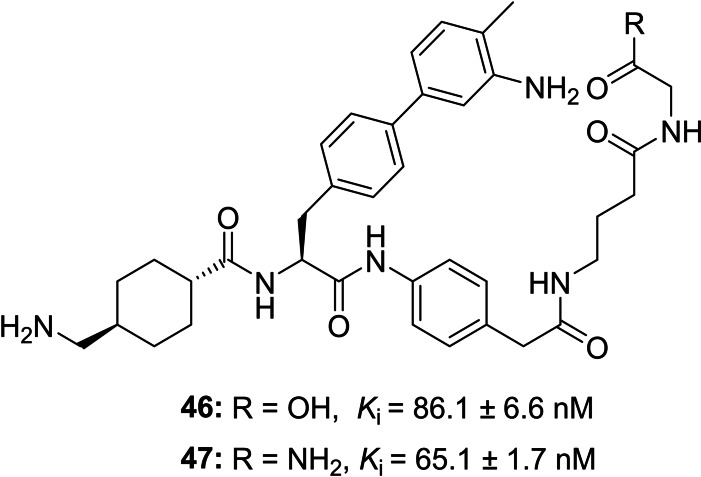
Plasmin inhibition by the acyclic inhibitors **46** and **47** derived from the cyclic analogue **28**.

Furthermore, two acyclic analogs of inhibitor **28** with a C‐terminal glycine or glycine amide have been prepared to investigate the influence of the cyclization on plasmin inhibition (Figure 2). Both compounds are at least 75‐fold weaker plasmin inhibitors than the cyclic analogue.

### Selectivity Studies

Selected inhibitors of the second series have also been tested with a set of related trypsin like serine proteases. For all inhibitors, a negligible inhibition with K_i_ values >4000 μM was found for the clotting proteases thrombin and fXa, as well as for the anticoagulant protease activated protein C (aPC) (Table 3). In contrast, an inhibition in the nanomolar range was observed for trypsin, plasma kallikrein (PK), and fXIa, despite all inhibitors are stronger plasmin inhibitors. For these three proteases a selectivity index (SI=*K*_i(protease)_/*K*_i(plasmin)_) was calculated, which is provided in brackets after the inhibition constants (Table 3). Especially compounds **32** and **33** potently inhibit fXIa and PK with *K*_i_ values≤15 nM, whereas inhibitor **28** is the strongest trypsin inhibitor (*K*_i_=33 nM). The most promising selectivity profile was found for compound **31**.

**Table 3:** Selectivity of selected inhibitors of the second series. For the inhibition of trypsin, PK, and fXIa an additional selectivity index (*K*_i(protease)_/K_i(plasmin)_) is provided in brackets.

No.	Plasmin	Trypsin	PK	fXIa	Thrombin	aPC	fXa
*K*_i_ (nM) and [selectivity index]							
28	0.832	33±1 [40]	31±14 [38]	41±3.2 [49]	>60000	>35000	>8000
30	2.08	800±80 [398]	60±12 [30]	47±2.0 [23]	>30000	>5000	>80000
31	0.831	550±30 [730]	67±4 [89]	88±23 [117]	>75000	>10000	>80000
32	1.46	140±10 [101]	15±1 [11]	5.3±1.4 [4]	>25000	>20000	>5000
33	0.524	130±20 [288]	9.4±2.1 [21]	8.1±1.6 [18]	>45000	>35000	>10000
40	9.47	100±10 [12]	120±10 [14]	110±10 [13]	>80000	>40000	>9000
45	2.53	120±20 [56]	156±35 [62]	320±50 [137]	>40000	>4000	>40000

### Structure of Inhibitor 28 in Complex with a μ‐Plasmin Mutant

**Figure 3:**
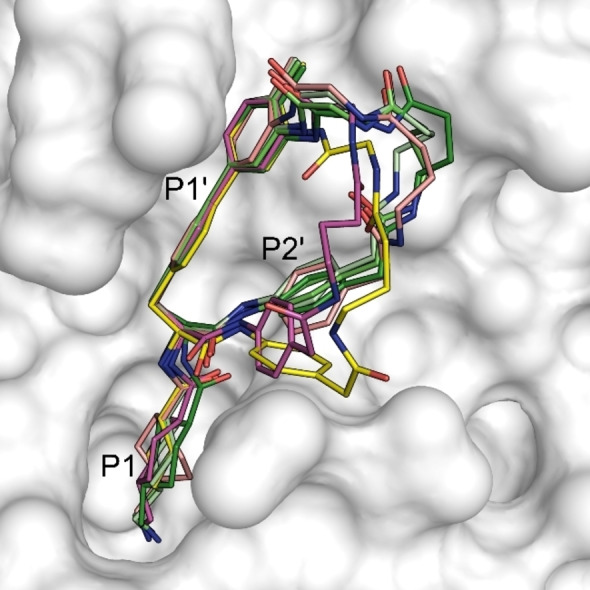
Superimposition of the crystal structures of inhibitor **28** taken from the six monomer complexes with Ser195Ala μ‐plasmin (shown with its surface in gray taken from complex A). The three macrocycles of the inhibitor shown with carbon atoms in different green colors adopt a very similar conformation (complexes A, C, and D), which differs from the ligands shown with carbon atoms in magenta (complex B), yellow (complex E) and salmon (complex F).

For inhibitor **28**, a crystal structure in complex with a stabilized Ser195(741)Ala mutant of μ–plasmin was determined. In this structure, six monomer complexes (namely A–F) were found per asymmetric unit. The P1 and P1’ residues are identically placed in all complexes. In three complexes (A, C, and D), the macrocycle (shown with carbon atoms in different green colors) adopts a similar structure indicating a preferred inhibitor conformation. Here, the electron densities of the complete ligands are well‐defined (Figure S1). In the three other complexes (B, E, and F), the P2’ residue and the adjacent linker are placed differently (Figure 3). Notably, here, the electron densities around the P2’ residue and linker segment of the ligands are not well defined, indicating a higher flexibility (Figure S1).

**Figure 4:**
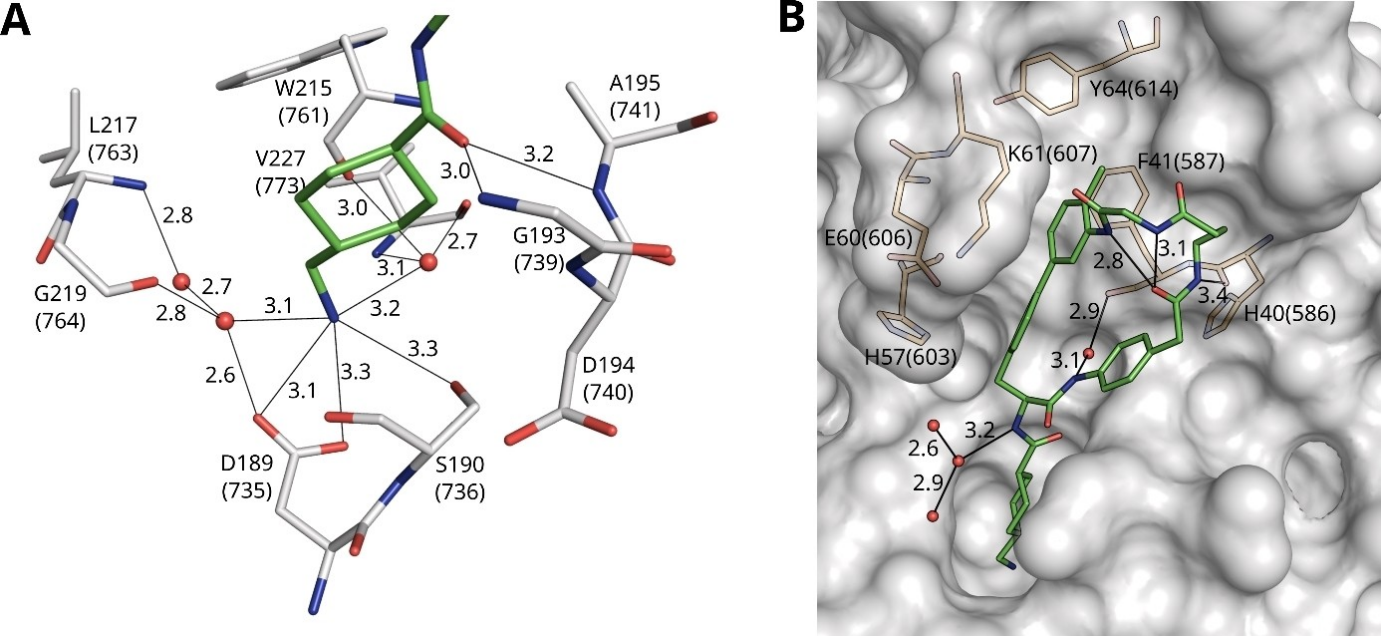
Crystal structure of inhibitor **28** taken from complex C in the active site of Ser195Ala μ‐plasmin. Polar contacts are shown as black lines with distances given in Å, and water molecules as red spheres. (A) Interactions of the P1 Txa residue (carbon atoms in green) within the S1 pocket (plasmin residues with carbons in white). (B) Intramolecular stabilization of the macrocycle by two hydrogen bonds between the carbonyl oxygen of the P2’ residue and the NH groups on the P1’ side chain and of the adjacent Gly residue within the linker. A bridging water molecule forms contacts between the P2’ NH and the carbonyl of Phe41(587), furthermore a weak contact exists between the NH of Gaba in the linker and the carbonyl of His40(586). Plasmin is shown as a transparent surface in gray, and its residues as sticks in orange.

Numerous polar contacts are formed from the P1 residue, which is similarly placed in all six complexes as shown for complex C in Figure 4A. The carbonyl oxygen of the Txa group makes H‐bonds to the amide NH of Gly193(739) and Ala195(741), which form the oxyanion hole. The P1 amino group is involved in a complex H‐bond network at the bottom of the S1 pocket to plasmin residues Asp189(735) and Ser190(736), and via three bridging water molecules also to residues Trp215(761), Leu 217(763), Gly219(764), and Val227(773) (Figure 4A).

The macrocycle of inhibitor **28** in complexes A, C, and D is stabilized by two intramolecular H‐bonds between the carbonyl oxygen of the P2’ residue and the NH on the P1’ biphenyl side chain and the NH of Gly from the linker (all distances between 3.1 and 2.8 Å, e. g., complex C in Figure 4B). These contacts may explain the strong preference of the Gaba‐Gly linker, when compared with the reverse Gly‐Gaba (**27**), one atom longer Ava‐Gly (**29**) or shorter β‐Ala‐Gly and Gly‐β‐Ala linkers (**25** and **26**). Although the P2’ carbonyl group in complex F (carbons in salmon) is similarly positioned as in complexes A, C, and D, here, these two distances are too long for a polar contact (4.1 and 4.0 Å). The completely different placement and rotation of the P2’ carbonyl group in complexes B and E (carbons in magenta and yellow, Figure 3), where the oxygen is rather directed towards the solvent, also prohibit the formation of these intramolecular contacts suggesting that these complexes do not adopt a preferred ligand conformation.

Only few additional polar contacts of the whole macrocycle to μ‐plasmin residues or surrounding water molecules could be detected in complexes A, C, and D (Figure 4B for complex C). A water molecule mediates a contact between the P2’ NH and the carbonyl oxygen of Phe41(587). However, the biphenyl side chain of the Bpa residue occupies very well the relatively long S1’ cleft between His57(603), Glu60(606), and Lys61(607) on the left side, as well as Phe41(587) on the right flank. The phenyl ring of the latter residue makes face‐to‐face contact with the terminal ring of the P1’ Bpa residue (closest C−C‐distance 3.7 Å), whereas an edge‐to‐face interaction is found between the other Bpa ring and the side chain of His57(603) (Figure 4B). The shortest distance of an imidazole nitrogen and the Cβ of Bpa is 3.7 Å. Possibly, also a cation‐π‐interaction between the Lys61(607) and Bpa side chains contributes to the binding affinity.

## Discussion

Nature provides a variety of peptidic and nonpeptidic macrocyclic compounds, some of them were successfully developed as drugs.^[26]^ This stimulated the design of fully‐synthetic macrocycles, also in the field of protease inhibitors. In recent years, cyclic inhibitors of the HCV NS3/4 A protease (Glecaprevir, Grazpprevir, Paritaprevir, Simeprevir, and Voxilaprevir) have been approved and the anticoagulant factor XIa inhibitor milvexian has entered clinical phase 3 studies. Like plasmin, fXIa belongs to the family of trypsin‐like serine proteases. A crystal structure revealed that milvexian occupies the S1 pocket and the prime side region of fXIa.^[27]^ In principle, the same binding regions are addressed by our newly developed plasmin inhibitors, as shown by the structure with inhibitor **28**. This distinguishes these compounds from our previously described cyclic inhibitors, which occupy the nonprime binding region in the active site of plasmin next to the S1 pocket.[^11^, ^12^]

The P1’ Bpa‐derivatives of the second series given in Table 2 possess an approximately 1000‐fold stronger potency compared with the initially prepared Phe(4‐NH_2_)‐derivatives shown in Table 1. The crystal structure of inhibitor **28** in complex with the used μ‐plasmin mutant revealed that the Bpa side chain perfectly fills the relatively long S1’ pocket, which is most probably only partially occupied with the shorter Phe(4‐NH_2_)‐derived inhibitors. Furthermore, it shows that the methyl substituent of inhibitor **28** in para position on the biphenyl moiety is not able to fill the empty space in the shallow pocket above the side chain of Tyr64(614) at the end of this binding cleft (Figure 4B). The slightly enhanced potency of inhibitor **33** (*K*_i_ 0.52 nM, Table 2) containing the bulkier isopropylamide substitution on the Bpa sidechain suggests that a further optimization of the substitution pattern in this position could improve the plasmin affinity, although the larger phenylamide analogue **32** was less potent. To our surprise, only very few polar contacts between the macrocyclic structure of inhibitor **28** and plasmin or surrounding water molecules were observed in the crystal structure, whereas a complex H‐bond network is formed from the P1 tranexamoyl residue. The conformation of the macrocycle is mainly stabilized by two intramolecular contacts from the carbonyl group of the 4‐amino‐phenylacetyl residue in P2’ position and two amide NH of the cycle. This suggests that the approximately 75‐fold stronger plasmin inhibition by inhibitor **28** compared to its acyclic analogues **46** and **47** (Figure 2) is most likely entropy driven by stabilizing a similar preferred ring conformation of the inhibitor in solution. This should result in a reduced entropic penalty during complex formation. Interestingly, all three particularly potent compounds (**28**, **31**, and **33**) possess a similar linker with eight backbone atoms and an amide bond at identical position, which should enable an analogue intramolecular H‐bond pattern within the macrocycle, as determined for inhibitor **28** in the crystal structure. The importance of these two intramolecular interactions for binding affinity is evident, as the relatively similar compound **27** with a reversed linker segment (Gly‐Gaba instead of Gaba‐Gly) and inhibitor **25** with a one atom shorter linker (β‐Ala‐Gly) inhibit plasmin at least 20 times less effectively. The impact is even more drastic with compound **29** containing a linker segment extended by one atom (Ava‐Gly). All these changes in the linker segment most likely prohibit the formation of at least one of these intramolecular polar contacts leading to a reduced inhibitory potency.

The best compounds with inhibition constants in the subnanomolar range like compound **31** already exhibit a preferred plasmin selectivity, although all of them also inhibit trypsin, PK, and fXIa in the nanomolar range (Table 3). So far, we do not yet know any clear structural feature in the prime side region that allows the development of highly selective plasmin inhibitors. In contrast, this was possible with our previously described macrocyclic plasmin inhibitors addressing the nonprime binding pockets, where we used the unique 94‐shunt (lack of the 99‐hairpin loop) of plasmin to achieve a superior selectivity profile. Although the design of highly selective compounds is usually favored for drug development to avoid side effects by addressing off‐targets, it is not clear, if the inhibition of trypsin, PK, and fXIa would be a real drawback for an injectable plasmin inhibitor used to reduce perioperative bleeding. Normally, trypsin is only found in the intestinal tract and therefore absent in the blood circulation. Hence, it should not come in contact with a parenterally given inhibitor. Furthermore, a moderate inhibition of PK associated with a beneficial anti‐inflammatory effect via reducing bradykinin levels was even described as a potential advantage of aprotinin, which inhibits PK in a similar range with a *K*_i_ value of 30 nM. A fXIa inhibition could be also acceptable, because in cardiac surgery with cardiopulmonary bypass, patients are usually co‐treated with coagulation inhibitors like heparin to avoid thrombosis. However, further *in vivo* experiments are required to prove these assumptions.

The most selective inhibitor **31** contains a hPhe residue in the linker segment, therefore, it is a relatively hydrophobic compound and possesses a reduced water solubility compared to inhibitor **28** with Gly in this position. This could be a disadvantage for an intravenous application in an antifibrinolytic therapy. Attempts to incorporate more water‐soluble moieties into the linker segment provided inhibitor **37** (*K*_i_=13 nM) as a promising candidate for further optimzation. The Lys‐ and piperazine‐containing compounds **34**–**38** with an additional protonatable group are at least 15 times weaker plasmin inhibitors than analogue **28**.

In summary, we have prepared first potent macrocyclic inhibitors addressing the prime‐side region of plasmin. Undoubtedly, these inhibitors can be further optimized, especially in P2’ position, the linker segment and the substitution pattern on the Bpa side chain. For the synthesis it is advantageous that large parts of the inhibitor backbone can be conveniently synthesized through standard SPPS. Additional issues for future work are the improvement of the selectivity profile and water solubility of these inhibitors.

## Experimental Section

### General Information

Reagents, solvents, amino acid derivatives were obtained from Acros Organics, Alfa Aesar, Bachem, BLDpharm, Carbolution, Fisher Scientific, Fluorochem, Iris Biotech, Merck KGaA and Roth and were used without further purification.

Analytical HPLC measurements were performed on a Primaide (VWR, Hitachi) system (column: NUCLEODUR C18 ec, 5 μm, 100 Å, 4.6 mm x 250 mm, Macherey‐Nagel) with 0.1 % TFA in water (solvent A) and 0.1 % TFA in acetonitrile (solvent B) as eluents using a linear gradient with an increase of 2 % B/min (method A, start at 10 % solvent B) or 1 % B/min (method B, start at indicated concentration of solvent B) at a flow rate of 1 mL/min and detection at 220 nm. Purifications via preparative HPLC were performed on a Knauer Azura system (pump P 2.1 L equipped with pump head E4099AB, detector UVD 2.1 L, Knauer GmbH, Berlin, Germany) using the same solvents as described above for analytical HPLC and a linear gradient with an increase of 0.5 % B/min at a flow rate of 20 mL/min (detection at 220 nm). After preparative HPLC, all inhibitors were obtained as lyophilized TFA‐salts in a purity >95 %. ESI mass spectra were measured on a QTrap 2000 ESI spectrometer (Applied Biosystems). NMR‐spectra were measured on a ECA500 (^1^H: 500 MHz, ^13^C: 126 MHz) with the respective deuterated solvent as internal standard. The chemical shifts δ are reported in ppm and the coupling constants J are given in Hz.

### Synthesis

The preparation of the most potent plasmin inhibitors from each series (compounds **12** and **33)** is described below. The synthesis of other intermediates and inhibitors is given in the supporting information.

### Synthesis of Inhibitor 12

(3‐(2‐(4‐((S)‐3‐(4‐nitrophenyl)‐2‐((1r,4S)‐4‐((2,2,2‐trifluoroacetamido)methyl)cyclohexane‐1‐carboxamido)propanamido)phenyl)acetamido)propanoyl)‐L‐phenylalanine **(1)**.

In a 5 mL syringe with filter frit, 2‐chlorotritylchloride resin (150 mg, 1 equiv, resin loading capacity 1.6 mmol/g) was treated with a solution of Fmoc‐Phe‐OH (93 mg, 0.24 mmol, 1 equiv) and DIPEA (125 μL, 0.72 mmol, 3 equiv) in 1.5 mL DCM and shaken at rt for 2 h. The solvent was removed and the resin was 3× washed with a mixture of DCM, MeOH and DIPEA (17/2/1, v/v/v), 2× with DCM, and 3×DMF.

The resin was treated with 20 % (v/v) piperidine in DMF (5 min and 15 min, respectively) and washed 9× with DMF. For the next coupling, a solution of Fmoc‐β‐alanine (224 mg, 0.72 mmol, 3 equiv), HATU (274 mg, 0.72 mmol, 3 equiv) and DIPEA (250 μL μL, 1.44 mmol, 6 equiv) was added. The syringe was shaken at rt for 1.5 h, the solvent was removed and the resin was washed 3× with DMF. The following amino acids were coupled analogously (Fmoc‐4‐aminophenylacetic acid: 269 mg, 0.72 mmol, 3 equiv; Fmoc‐Phe(4‐NO_2_)‐OH: 311 mg, 0.72 mmol, 3 equiv); and Tfa‐Txa‐OH: 182 mg, 0.72 mmol, 3 equiv). After the last coupling step, the resin was subsequently washed 9× with DMF and 3× with DCM. The peptide was cleaved from resin thrice with a solution of 1 % TFA in DCM (v/v) for 30 min, respectively. The eluates were combined and the solvent was removed *in vacuo*. The crude intermediate **1** was obtained as yellow oil and used for the next step without further purification (HPLC method A: 24.73 min, MS calcd.: 796.30, m/z found: 795.70 [M−H]^−^).

(3‐(2‐(4‐((S)‐3‐(4‐aminophenyl)‐2‐((1r,4S)‐4‐((2,2,2‐trifluoroacetamido)methyl)cyclohexane‐1‐carboxamido)propanamido)phenyl)acetamido)propanoyl)‐L‐phenylalanine **(2)**.

The crude compound **1** was dissolved in 150 mL 90 % aq. AcOH and the mixture was stirred under a hydrogen atmosphere at ambient pressure and rt overnight. After filtration, the solvent was removed *in vacuo* and the product was purified by preparative HPLC (33 mg, 0.037 mmol as colorless lyophilized solid, HPLC method A: 17.10 min, MS calcd.: 766.33, m/z found: 767.47 [M+H]^+^.

(1r,4S)‐4‐(aminomethyl)‐N‐((4S,9S)‐9‐benzyl‐3,8,11,15‐tetraoxo‐2,7,10,14‐tetraaza‐1,6(1,4)‐dibenzenacyclohexadecaphane‐4‐yl)cyclohexane‐1‐carboxamide×TFA **(12)**.

Intermediate **2** (33 mg, 0.037 mmol 1 equiv) dissolved in 33 mL DMF was stirred at 0 °C and treated with solutions of HATU (18 mg, 0.048 mmol, 1.3 equiv) dissolved in 5 mL DMF and DIPEA (17 μL, 0.096 mmol, 2.6 equiv) dissolved in 5 mL DMF in ten similar portions, respectively, within 30 minutes. The mixture was stirred at 0 °C for 1 h and at rt overnight, afterwards the solvent was removed *in vacuo* (HPLC method A: 22.29 min, MS calc.: 748.32, m/z found: 749.50 [M+H]^+^). The obtained cyclized Tfa‐protected crude intermediate was suspended in 3 mL of a 4 : 1 mixture of 1,4‐dioxane and aq. 1 N NaOH solution, followed by dropwise addition of DMSO until dissolution of the mixture. After stirring at rt for 1 h, the obtained clear solution was neutralized with TFA, the solvent was removed *in vacuo* and the product was purified by preparative HPLC (6 mg as colorless lyophilized solid, 0.008 mmol, 21 %, HPLC method B, start at 15 % B: 14.90 min, purity >98 %, MS calcd.: 652.34, m/z found: 653.37 [M+H]^+^. ^1^H NMR (500 MHz, DMSO‐*d*_6_): δ[ppm]=9.85 (s, 0.5H), 9.62 (s, 0.5H), 8.90 (s, 0.5H), 8.85 (s, 0.5H), 8.21 (d, ^*3*^ *J*=8.5 Hz, 0.5H), 8.13 (d, ^*3*^ *J*=8.1 Hz, 0.5H), 8.10 (d, ^*3*^ *J*=7.4 Hz, 0.5H), 8.04 (d, ^*3*^ *J*=7.3 Hz, 0.5H), 7.72–7.60 (m, 3H), 7.35 (d, ^*3*^ *J*=8.7 Hz, 1H), 7.28–7.12 (m, 7H), 7.02 (d, ^*3*^ *J*=8.2 Hz, 1H), 6.97 (d, ^*3*^ *J*=8.5 Hz, 1H), 6.89 (d, ^*3*^ *J*=8.5 Hz, 1H), 6.80 (d, ^*3*^ *J*=8.5 Hz, 1H), 6.73 (d, ^*3*^ *J*=8.4 Hz, 2H), 4.62–4.52 (m, 1.5H), 4.45 (dt, ^*2*^ *J*=11.4 Hz, ^*3*^ *J*=6.8 Hz, 0.5H), 3.19–3.09 (m, 3H), 3.07–2.90 (m, 2H), 2.84–2.75 (td, ^*2*^ *J*=13.6 Hz, ^*3*^ *J*=9.1 Hz, 1H), 2.65 (p, ^*3*^ *J*=5.7 Hz, 2H), 2.61–2.51 (m, 1H), 2.42–2.34 (m, 2H), 2.24–2.16 (m, 1H), 2.13–2.05 (m, 1H), 1.81–1.68 (m, 4H), 1.53–1.42 (m, 1H), 1.38–1.26 (m, 2H), 0.94 (qd, ^*2*^ *J*=12.6 Hz, ^*3*^ *J*=3.0 Hz, 2H).

### Synthesis of Inhibitor 33

4‐Bromo‐N‐Isopropyl‐2‐Nitrobenzamide (14).

Isopropylamine (241 mg, 4.08 mmol, 1 eq.) and 4‐bromo‐2‐nitrobenzoic acid **13** (1.00 g, 4.08 mmol, 1 eq.) were dissolved in 15 mL DMF, cooled to 0 °C and treated with DIPEA (1.78 mL, 10.2 mmol, 2.5 eq.). Under vigorous stirring, HATU (2.02 g, 5.30 mmol, 1.3 eq.) was added in 10 similar portions within 10 minutes. After 1 h, the ice bath was removed and the mixture was stirred at rt overnight. The solvent was removed *in vacuo* and the crude product was dissolved in in a mixture of ethyl acetate and 5 % aqueous KHSO_4_ solution. The aqueous layer was extracted thrice with ethyl acetate and the combined organic layers were washed thrice with 5 % (m/v) aqueous KHSO_4_, 1× with brine, thrice with saturated aqueous NaHCO_3_ solution and once with brine. The organic layer was dried over anhydrous MgSO_4_ and filtered before the solvent was removed *in vacuo*. The product was purified by preparative HPLC (1.10 g as colorless solid, 3.82 mmol, 94 %, HPLC method A: 21.44 min, purity >98 %, MS calcd.: 286.00, m/z found: 286.98 [M+H]^+^, ^1^H NMR (500 MHz, DMSO‐*d*_6_): δ[ppm]=8.52 (d, ^*3*^ *J*=7.6 Hz, 1H), 8.25 (d, ^*4*^ *J*=1.8 Hz, 1H), 7.99 (dd, ^*3*^ *J*=8.2, ^*4*^ *J*=1.9 Hz, 1H), 7.53 (d, ^*3*^ *J*=8.2 Hz, 1H), 4.07–3.84 (m, 1H), 1.14 (d, ^*3*^ *J*=6.6 Hz, 6H). ^13^C NMR (126 MHz, DMSO‐*D*_6_): δ[ppm]=163.48, 147.71, 136.08, 131.68, 130.76, 126.57, 122.41, 41.13, 21.84.

(4‐(Isopropylcarbamoyl)‐3‐Nitrophenyl)Boronic Acid (15).

Intermediate **13** (1.05 g, 3.65 mmol, 1 eq.), bis(pinakolato)diboron (B_2_pin_2_, 1.02 g, 4.02 mmol, 1.1 eq.), Pd(dppf)Cl_2_ (89 mg, 0.11 mmol, 0.03 eq.) and potassium acetate (1.08 g, 11 mmol, 3 eq.) were suspended in 105 mL dimethoxyethane in a Schlenk flask equipped with a reflux condenser. Under rapid stirring, an argon atmosphere was established by evacuating and subsequently flooding the system with argon gas for ten times, respectively. After boiling under reflux for 3 h, the mixture was filtered and the solvent removed *in vacuo*. To remove the pinacol group, the residue was treated with a 9 : 1 mixture of water and acetonitrile supplemented with 0.1 % (v/v) TFA for 1 h at rt, followed by purification with preparative HPLC (685 mg colorless solid, 2.7 mmol, 74 %, HPLC method A: 12.88 min, purity >98 %, MS calcd.: 252.09, m/z found: 253.12 [M+H]^+^, ^1^H NMR (500 MHz, DMSO‐*d*_6_): δ[ppm]=8.46 (d, ^*3*^ *J*=7.7 Hz, 1H), 8.36 (d, ^*4*^ *J*=0.9 Hz, 1H), 8.11 (dd, ^*3*^ *J*=7.5 Hz, ^*4*^ *J*=1.1 Hz, 1H), 7.53 (d, ^*3*^ *J*=7.5 Hz, 1H), 4.07–3.94 (m, 1H), 1.15 (d, ^*3*^ *J*=6.6 Hz, 6H). ^13^C NMR (126 MHz, DMSO‐*D*_6_): δ[ppm]=164.64, 146.39, 138.90, 134.30, 128.75, 128.24, 40.99, 21.90.

(S)‐2‐Amino‐3‐(4′‐(Isopropylcarbamoyl)‐3′‐Nitro‐[1,1′‐Biphenyl]‐4‐Yl)Propanoic Acid×TFA (16).

Boc‐Phe(4‐Br)‐OH (668 mg, 1.94 mmol, 1 eq.), the boronic acid **15** (636 mg, 2.53 mmol, 1.3 eq.), Pd(dppf)Cl_2_ (82 mg, 0.10 mmol, 0.04 eq.), 2 N aqueous Cs_2_CO_3_ solution (3.16 mL, 2.07 g Cs_2_CO_3_ 6.32 mmol, 3.25 eq.) were suspended in 35 mL dimethoxyethane in a Schlenk flask equipped with a reflux condenser. Under rapid stirring, an argon atmosphere was established by evacuating and subsequently flooding the system with argon gas for ten times, respectively. After boiling under reflux for 4 h, the mixture was filtered and the solvent removed *in vacuo*. The residue was dissolved in a mixture of ethyl acetate and aqueous 20 % (w/v) citric acid solution. The layers were separated and the organic layer was washed thrice with the aforementioned citric acid solution and once with brine. The solvent was removed and the Boc‐protected intermediate (HPLC method A: 25.14 min) was treated with 5 mL 4 N HCl in 1,4‐dioxane for 2 h at rt. The product was precipitated with diethyl ether and purified by preparative HPLC (786 mg colorless lyophilized solid, 1.62 mmol, 83 %, HPLC method A: 14.55 min, purity >95 %, MS calcd.: 371.15, m/z found: 372.14 [M+H]^+^, ^1^H NMR (500 MHz, DMSO‐*d*_6_): δ[ppm]=8.53 (d, *J*=7.7 Hz, 1H), 8.24 (d, ^*4*^ *J*=1.8 Hz, 1H), 8.06 (dd, ^*3*^ *J*=8.0 Hz, ^*4*^ *J*=1.8 Hz, 1H), 7.77 (d, ^*3*^ *J*=8.3 Hz, 2H), 7.65 (d, ^*3*^ *J*=8.0 Hz, 1H), 7.43 (d, ^*3*^ *J*=8.3 Hz, 2H), 4.11–3.95 (m, 2H), 3.19 (dd, ^*2*^ *J*=14.3 Hz, ^*3*^ *J*=5.8 Hz, 1H), 3.10 (dd, ^*2*^ *J*=14.3 Hz, ^*3*^ *J*=7.0 Hz, 1H), 1.16 (d, ^*3*^ *J*=6.6 Hz, 6H). ^13^C NMR (126 MHz, DMSO‐*D*_6_): δ[ppm]=170.16, 164.15, 147.87, 141.75, 136.63, 135.74, 131.27, 130.74, 130.34, 129.71, 127.00, 121.39, 53.72, 41.07, 35.79, 21.91.

(S)‐2‐((((9H‐Fluoren‐9‐yl)Methoxy)Carbonyl)Amino)‐3‐(4′‐(Isopropylcarbamoyl)‐3′‐Nitro‐[1,1′‐Biphenyl]‐4‐yl)Propanoic Acid or Fmoc‐Bpa(4‐Ipa,3‐NO_2_)‐OH (17).

Compound **16** (786 mg, 1.62 mmol, 1 eq.) was suspended in 10 mL dichloromethane and stirred at 0 °C in a water ice bath. The mixture was treated with chlorotrimethylsilane (TMSCl, 387 mg, 3.56 mmol, 2.2 eq.) in one portion and DIPEA (931 μL, 5.34 mmol, 3.3 eq.) was added dropwise within 10 minutes. After stirring for 5 minutes at 0 °C and subsequently boiling under reflux for 30 minutes, the obtained clear solution was cooled to 0 °C, before a solution of Fmoc‐Cl (406 mg, 1.57 mmol, 0.97 eq.) in 5 mL DCM was added dropwise within 10 minutes. The mixture was stirred at 0 °C for 5 minutes and again boiled under reflux for 30 minutes. After cooling to room temperature, 3 mL of an aqueous 10 % (w/v) (NH_4_)_2_SO_4_ solution (pH adjusted to 1 with conc. H_2_SO_4_) were added before the solvent was removed *in vacuo*. The remaining residue was dissolved in a mixture of ethyl acetate and the aforementioned (NH_4_)_2_SO_4_ solution. The aqueous layer was separated and extracted thrice with ethyl acetate. Then, the combined organic layers were washed thrice with the (NH_4_)_2_SO_4_ solution and once with brine, dried over anhydrous MgSO_4_ and filtered before the solvent was removed *in vacuo* (869 mg colorless solid, 1.51 mmol, 93 %, HPLC method A: 29.92 min, purity >97 %. MS calcd.: 593.22, m/z found: 594.30 [M+H]^+^, ^1^H NMR (500 MHz, DMSO‐*d*_6_): δ[ppm]=12.78 (bs, 1H), 8.52 (d, ^*3*^ *J*=7.8 Hz, 1H), 8.20 (d, ^*4*^ *J*=1.7 Hz, 1H), 8.02 (dd, ^*3*^ *J*=8.0 Hz, ^*4*^ *J*=1.7 Hz, 1H), 7.87 (d, ^*3*^ *J*=7.6 Hz, 2H), 7.76 (d, ^*3*^ *J*=8.6 Hz, 1H), 7.70 (d, ^*3*^ *J*=8.2 Hz, 2H), 7.66–7.61 (m, 3H), 7.45–7.36 (m, 4H), 7.35–7.22 (m, 2H), 4.28–4.19 (m, 2H), 4.18–4.13 (m, 2H), 4.06–3.93 (m, 1H), 3.16 (dd, ^*2*^ *J*=13.9 Hz, ^*3*^ *J*=4.2 Hz, 1H), 2.95 (dd, ^*2*^ *J*=13.8 Hz, ^*3*^ *J*=10.8 Hz, 1H), 1.16 (d, ^*3*^ *J*=6.6 Hz, 6H). ^13^C NMR (126 MHz, DMSO‐*D*_6_): δ[ppm]=173.13, 164.17, 155.92, 147.83, 143.69, 141.91, 140.62, 138.92, 135.18, 131.15, 130.72, 129.98, 129.63, 127.55, 126.98, 126.74, 125.19, 121.37, 120.03, 65.56, 55.26, 46.53, 41.05, 39.85, 36.03, 21.92.

(4‐(2‐(4‐((S)‐3‐(3′‐Amino‐4′‐(Isopropylcarbamoyl)‐[1,1′‐Biphenyl]‐4‐yl)‐2‐((1r,4S)‐4‐((2,2,2‐Trifluoroacetamido)Methyl)Cyclohexane‐1‐Carboxamido)Propanamido)Phenyl)Acetamido)Butanoyl)Glycine (21).

Precursor **20** was synthesized according to a standard Fmoc‐SPPS protocol described above for compound **12**. 150 mg of 2‐CTC resin (loading capacity 1.6 mmol/g, absolute 0.24 mmol) were loaded as described above with Fmoc‐Gly‐OH (71 mg, 0.24 mmol, 1 eq.) and 125 μL DIPEA (0.72 mmol, 3 eq.). Then, the following amino acids were coupled subsequently: Fmoc‐Gaba‐OH (234 mg, 0.72 mmol, 3 eq.), Fmoc‐4‐aminophenylacetic acid (269 mg, 0.72 mmol, 3 equiv), compound **17** (427 mg, 0.72 mmol, 3 eq.) and Tfa‐Txa‐OH (182 mg, 0.72 mmol, 3 equiv). After cleavage from the resin with 2 % TFA in DCM and neutralization of the eluates, the solvent was removed *in vacuo* yielded the crude nitro‐substituted intermediate **20** (HPLC method A: 25.15 min, MS calc.: 915.34, m/z found: 914.50 [M−H]^−^). The yellow oil was dissolved in 150 mL 90 % aq. AcOH and the mixture was stirred under a hydrogen atmosphere at ambient pressure and rt overnight. After filtration, the solvent was removed *in vacuo* and the acyclic precursor **21** was purified by preparative HPLC (136 mg, 0.19 mmol, 79 % colorless lyophilized solid, HPLC method A: 20.27 min, purity >97 %. MS calcd.: 851.38, m/z found: 852.51 [M+H]^+^, ^1^H NMR (500 MHz, DMSO‐*d*_6_): δ[ppm]=12.38 (bs, 1H), 10.02 (s, 1H), 9.33 (t, ^*3*^ *J*=5.9 Hz, 1H), 8.11 (t, ^*3*^ *J*=5.9 Hz, 1H), 8.04 (d, ^*3*^ *J*=8.3 Hz, 1H), 7.99 (d, ^*3*^ *J*=7.8 Hz, 1H), 7.96 (t, ^*3*^ *J*= 5.6 Hz, 1H), 7.58 (d, ^*3*^ *J*=8.3 Hz, 1H), 7.53–7.47 (m, 4H), 7.35 (d, ^*3*^ *J*=8.3 Hz, 2H), 7.18 (d, ^*3*^ *J*=8.6 Hz, 2H), 7.00 (d, ^*4*^ *J*=1.8 Hz, 1H), 6.84 (dd, ^*3*^ *J*=8.2 Hz, ^*4*^ *J*=1.7 Hz, 1H), 4.68 (td, ^*3*^ *J*=8.8 Hz, 5.1 Hz, 1H), 4.13–4.03 (m, 1H), 3.73 (d, ^*3*^ *J*=5.9 Hz, 2H), 3.34 (s, 2H), 3.12–2.97 (m, 5H), 2.90 (dd, ^*2*^ *J*=13.7 Hz, ^*3*^ *J*=9.5 Hz, 1H), 2.20–2.04 (m, 3H), 1.77–1.54 (m, 6H), 1.48–1.37 (m, 1H), 1.30–1.20 (m, 2H), 1.16 (d, ^*3*^ *J*=6.6 Hz, 6H), 1.01–0.68 (m, 2H). ^13^C NMR (126 MHz, DMSO‐*d*_6_): δ[ppm]=175.01, 172.14, 171.33, 170.09, 170.05, 167.58, 156.30 (pd, ^*2*^ *J*_*C‐F*_=35.9 Hz), 142.73, 137.61, 137.48, 137.09, 131.48, 129.70, 129.14, 128.85, 126.05, 119.28, 117.13, 114.29, 113.66, 54.32, 45.07, 43.44, 41.78, 40.52, 40.44, 38.29, 37.40, 36.33, 32.56, 29.35, 29.25, 28.49, 28.32, 25.28, 22.29.

(S)‐4‐((1r,4S)‐4‐(aminomethyl)cyclohexane‐1‐carboxamido)‐N‐isopropyl‐5,9,14,17‐tetraoxo‐6,10,15,18‐tetraaza‐1(1,3),2,7(1,4)‐tribenzenacyclooctadecaphane‐14‐carboxamide x TFA **(33)**.

Compound **21** (136 mg, 0.19 mmol, 1 eq.) was cyclized as described above for compound **12** using HATU (94 mg, 0.25 mmol, 1.3 eq.) and DIPEA (86 μL, 0.49 mmol 2.6 eq.) in 136 mL of DMF. After removal of the solvent, the crude product (HPLC method A: 23.57 min) was suspended in 4 mL of a 4 : 1 mixture of 1,4‐dioxane and aq. 1 N NaOH solution. A few drops of THF and acetone were added until the mixture cleared up significantly. After stirring at rt for 1 h, the obtained clear solution was neutralized with TFA, the solvent was removed *in vacuo* and the crude inhibitor was purified by preparative HPLC (69 mg colorless lyophilized solid, 0.08 mmol, 42 % over two steps, HPLC method A: 16.12 min, purity >99 %, MS calc.: 737.39, m/z found: 738.51 [M+H]^+^. ^1^H NMR (500 MHz, DMSO‐*d*_6_): δ[ppm]=11.55 (s, 1H), 9.84 (s, 1H), 8.64 (d, ^4^ *J*=1.8 Hz, 1H), 8.54 (d, ^3^ *J*=7.7 Hz, 1H), 8.23 (t, ^*3*^ *J*=5.9 Hz, 1H), 8.10 (d, ^*3*^ *J*=7.5 Hz, 1H), 8.00 (t, ^*3*^ *J*=5.8 Hz, 1H), 7.84 (d, ^*3*^ *J*=8.4 Hz, 1H), 7.71 (s, 3H), 7.51 (d, ^*3*^ *J*=8.3 Hz, 2H), 7.42–7.36 (m, 3H), 7.24 (d, ^*3*^ *J*=8.3 Hz, 2H), 7.18 (d, ^*3*^ *J*=8.6 Hz, 2H), 4.65–4.60 (m, 1H), 4.18–4.09 (m, 1H), 3.85 (d, ^*3*^ *J*=6.1 Hz, 2H), 3.32–3.22 (m, 1H), 3.08–2.95 (m, 4H), 2.73–2.64 (m, 2H), 2.32–2.23 (m, 1H), 2.14–2.00 (m, 2H), 1.89–1.73 (m, 4H), 1.65–1.57 (m, 2H), 1.56–1.47 (m, 1H), 1.44–1.29 (m, 2H), 1.24–1.14 (m, 6H), 1.04–0.90 (m, 2H). ^13^C NMR (126 MHz, DMSO‐*d*_6_): δ[ppm]=175.14, 173.25, 170.53, 169.64, 168.29, 167.58, 143.76, 140.09, 137.95, 137.49, 137.44, 132.24, 130.51, 129.42, 129.39, 127.23, 120.82, 120.04, 119.42, 118.60, 55.39, 44.94, 43.54, 43.47, 43.05, 41.66, 38.94, 38.90, 35.66, 33.83, 29.48, 29.42, 28.97, 28.85, 27.49, 27.04, 22.64, 22.63.

### Kinetic Measurements

All measurements were conducted as triplicates in black 96‐well FluoroNunc MaxiSorp plates (Nunc, Langenselbold, Germany) and suitable fluorogenic substrates at room temperature. Measurements with human plasmin (molecular weight 78 kDa, Calbiochem, San Diego, California, USA) were performed using a Tecan Spark® microplate reader (Tecan Group AG, Männedorf, Switzerland) in 50 mM TRIS‐HCl buffer pH 8.0 containing 154 mM NaCl (λ_ex_=380 nm and λ_em_=460 nm) with eight different inhibitor concentrations and a control in absence of inhibitor. Each well contained a total volume of 180 μL consisting of 100 μL of inhibitor dissolved in buffer, 40 μL of the substrate Mes‐dArg‐Phe‐Arg‐AMC^[11]^ (100 μM in assay, K_M_=16.1 μM) dissolved in water and 40 μL of plasmin solution (0.27 nM in assay). The following equations were used for the enzyme kinetic analysis as described in the results section and in our previous publications.[^11^, ^12^]
(1)

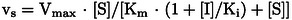



(2)

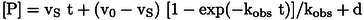



(3)

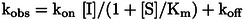



(4)

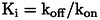




The measurements with the related trypsin‐like serine proteases trypsin, plasma kallikrein, factor XIa, thrombin, activated protein Ca and factor Xa used in selectivity studies were performed as described previously.[^11^, ^12^] All K_i_ values in the micromolar range, which are given with a greater than sign in Table 3, were deduced from pre‐assays with only three different inhibitor concentrations. From these measurements inhibition constants were calculated, which were slightly lower than the given values.

### Crystallography

#### Recombinant Protein Production and Crystallization

The serine protease domain of human plasminogen (μ‐plasminogen) was expressed as the active‐site mutant Ser195(741)Ala, followed by tPA activation into μ‐plasmin and purification as previously described.^[9]^ For crystallization, about 10 mg/mL of purified μ‐plasmin and excess inhibitor **28** (1 : 1.5 molar ratio) were mixed and crystals were obtained by hanging drop vapor phase diffusion method at 20 °C. The protein sample (2 μL) was mixed with 1 μL of well solution containing 0.1 M Tris‐HCl pH 8.5 and 0.2 M NH_4_H_2_PO_4_.

#### Structure Determination

Single crystals of μ‐plasmin/inhibitor **28** complex were quickly cooled in liquid nitrogen with 15 % glycerol as the cryoprotectant and diffracted at the Australian Synchrotron using MX2 beamline. Data was processed using XDS^[28]^ and the complex structures were built by molecular replacement in COOT^[29]^ using the μ‐plasmin (PDB ID 5UGG) as the searching model. The macrocyclic inhibitor **28** was generated by eLBOW and fit into the electron density map using LigandFit in Phenix.^[30]^ The final structure was further refined by Phenix and Coot. The data collection and refinement statistics are summarized in Table S1. The structure of μ‐plasmin in complex with inhibitor **28** was deposited in the Protein Data Bank with accession code 9AZK. Figures were generated using PyMOL.^[31]^

## Abbreviations

AcOH, acetic acid; Boc, *tert*‐Butyloxycarbonyl; Bpa, biphenylalanine; B_2_pin_2_, Bis(pinacolato)diboron; 2‐CTC, 2‐chlorotrityl chloride; DCM, dichloromethane; DIPEA, diisopropylethyl amine; DMF, dimethylformamide; DMSO, dimethylsulfoxide; Fmoc, fluorenyloxycarbonyl; fXa, factor Xa; Gaba, 4‐aminobutyric acid; HATU, 1‐[bis(dimethylamino)methylene]‐1*H*‐1,2,3‐triazolo[4,5‐b]pyridinium 3‐oxide hexafluoro‐phosphate; Ipa, isopropylamide; Pd(dppf)Cl_2,_ [1,1′‐Bis(diphenylphosphino)ferrocene]palladium(II) dichloride; Pha, phenylamide; Tfa, trifluoroacetyl; TFA, trifluoroacetic acid, Txa, tranexamoyl, TXA tranexamic acid.

## Conflict of Interests

The authors declare no conflict of interest.

1

## Supporting information

As a service to our authors and readers, this journal provides supporting information supplied by the authors. Such materials are peer reviewed and may be re‐organized for online delivery, but are not copy‐edited or typeset. Technical support issues arising from supporting information (other than missing files) should be addressed to the authors.

Supporting Information

## Data Availability

The data that support the findings of this study are available from the corresponding author upon reasonable request.
