# Supplementary material for: Macrocyclic Inhibitors Targeting the Prime Site of the Fibrinolytic Serine Protease Plasmin
Source: ChemMedChem. 2024 Sep 30;19(23):e202400360. doi: 10.1002/cmdc.202400360 (PMC11617653; doi:10.1002/cmdc.202400360)
Supplement: Supplementary file 1 — Supporting Information [file CMDC-19-e202400360-s001.pdf]

ChemMedChem

Supporting Information

Macrocyclic Inhibitors Targeting the Prime Site of the Fibrinolytic Serine Protease Plasmin

Simon J. A. Wiedemeyer, Guojie Wu, Heike Lang-Henkel, James C Whisstock, Ruby H. P. Law, and Torsten Steinmetzer*

Content

1.	General information and methods	S2
2.	Synthesis of P1 building blocks	S3
3.	Synthesis of P1' building blocks and their intermediates	S4
4.	Synthesis of P2' building blocks	S8
5.	Synthesis of building blocks and intermediates for linker segment	S10
6.	Synthesis of inhibitors of first series (Table 1 in main manuscript)	S13
7.	Synthesis of inhibitors of second series (Table 2 in main manuscript)	S23
8.	Synthesis of linear reference inhibitors **46** and **47**	S49
9.	Structure determination of μ-plasmin mutant in complex with inhibitor **28**	S51
10.	Abbreviations	S52

1. General information and methods

Reagents, solvents, amino acid derivatives were obtained from Acros Organics, Alfa Aesar, Bachem, BLDpharm, Carbolution, Fisher Scientific, Fluorochem, Iris Biotech, Merck KGaA and Roth and were used without further purification.

Analytical HPLC measurements were performed on a Primaide (VWR, Hitachi) system (column: NUCLEODUR C18 ec, 5 µm, 100 Å, 4.6 mm x 250 mm, Macherey-Nagel) with 0.1 % TFA in water (solvent A) and 0.1 % TFA in acetonitrile (solvent B) as eluents using a linear gradient with an increase of 2 % B/min (method A, start at 10 % solvent B) or 1 % B/min (method B, start at indicated concentration of solvent B) at a flow rate of 1 mL/min and detection at 220 nm. Purifications via preparative HPLC were performed on a Knauer Azura system (pump P 2.1 L equipped with pump head E4099AB, detector UVD 2.1L, Knauer GmbH, Berlin, Germany) using the same solvents as described above for analytical HPLC and a linear gradient with an increase of 0.5 % B/min at a flow rate of 20 mL/min (detection at 220 nm). After preparative HPLC, all inhibitors were obtained as lyophilized TFA-salts in a purity > 95 %. Some intermediates were purified by preparative MPLC using a Teledyne Isco system (CombiFlash Sg 100C, UA-6 UV/Vis detector, absorbance at λ = 254 nm, Foxy Jr. fraction collector, Thousand Oaks, CA, USA), the respective FlashPure silica cartridges (12 g or 40 g silica gel, irregular particle size 35 - 45 µM) were purchased from Büchi Labortechnik AG (Flawil, CH). Thereby, gradient methods with mixtures of apolar and polar solvents were applied as stated in the respective experimental protocols, whereas the flow rate was selected depending on the applied silica cartridge (12 g silica gel at 30 mL per minute, 40 g silica gel at 40 mL per minute). After keeping the respective starting conditions constant for 3 minutes, a linear gradient of +1 % (v/v) per minute was applied. The individual conditions are stated in the respective protocols. The product containing fractions were dried in vacuo.

ESI mass spectra were measured on a QTrap 2000 ESI spectrometer (Applied Biosystems). NMR-spectra were measured on an ECA500 (^{1}H at 500 MHz, ^{13}C at 126 MHz) with the respective deuterated solvent as internal standard (s singlet, d doublet, dd doublet of doublet, t triplet, td triplet of doublet, q quartet, qd quartet of doublet, quintet, multiplet, br broad signal, prefix p pseudo, prefix b broad). The chemical shifts δ are reported in ppm and the coupling constants *J* are given in Hz.

Certain building blocks used as P1, P1', P2' and linker residues during Fmoc-SPPS were prepared by solution synthesis as described below and are numbered as S1, S2, S3, etc. The numbers of the final inhibitors correspond to the numbering used in the main manuscript, their linear peptidic precursors are named with the same number and an additional letter (a for the nitro derivative, b for the reduced amine compound, and c for the cyclized but still protected intermediate).

2. Synthesis of P1 building blocks

(1r,4r)-4-((2,2,2-Trifluoroacetamido)methyl)cyclohexane-1-carboxylic acid or Tfa-Txa-OH **(S1)**

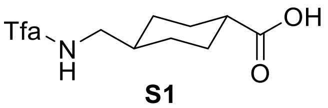

S1

For the incorporation of the protecting group, a previously described general procedure was adapted.[21] Tranexamic acid (2.00 g, 12.7 mmol, 1 eq.) was suspended in 30 mL DCM, cooled in an ice bath, and treated with chlorotrimethylsilane (TMSCl, 3.04 g, 28.0 mmol, 2.2 eq.), DIPEA (4.87 mL, 28 mmol, 2.2 eq.). After 5 min, the mixture was heated and refluxed for 30 min. After cooling in an ice bath, trifluoroacetic anhydride (2.94 g, 14 mmol, 1.1 eq.) dissolved in 8 mL DCM was added dropwise within 30 min. The mixture was stirred 5 min at 0 °C and then refluxed again for 30 minutes. After aqueous workup (see synthesis of intermediate **17** in the main manuscript), the compound was used for Fmoc-SPPS without further purification (2.95 mg as colorless solid, 11.68 mmol, 92 %, MS calcd.: 253.09, m/z found: 252.14 $[M-H]^-$, ^{1}H NMR (500 MHz, DMSO-d_6): δ[ppm] = 11.96 (s, 1H), 9.36 (t, ^3J = 5.6 Hz, 1H), 3.03 (t, ^3J = 6.4 Hz, 2H), 2.12 (tt, $^3J_{ax.-ax.}$ = 12.1 Hz, $^3J_{ax.-eq.}$ = 3.6 Hz, 1H), 1.89 (pdd, $^3J_{ax.-ax.}$ = 13.8 Hz, $^3J_{ax.-eq.}$ = 3.3 Hz, 2H), 1.70 (dd, $^3J_{ax.-ax.}$ = 13.7 Hz, $^3J_{ax.-eq.}$ = 3.2 Hz, 2H), 1.55 - 1.39 (m, 1H), 1.25 (qd, ^2J = 13.1 Hz, ^3J = 3.4 Hz, 2H), 0.93 (qd, ^2J = 13.2 Hz, ^3J = 3.5 Hz, 2H). ^{13}C NMR (126 MHz, DMSO-d_6): δ[ppm] = 176.51, 156.29 (q, $^2J_{C-F}$ = 35.9 Hz), 44.99, 42.28, 36.30, 29.14, 28.06.

(1r,4r)-4-(((Tert-butoxycarbonyl)amino)methyl)cyclohexane-1-carboxylic acid or Boc-Txa-OH **(S2)**

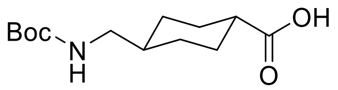

S2

Tranexamic acid (500 mg, 3.18 mmol, 1 eq.) was dissolved in 10 mL 1,4-dioxane and treated with 1 N aqueous NaOH solution (6.4 mL, 6.4 mmol, 2 eq.). The mixture was cooled to 0 °C in an ice bath and di-tert-butyl dicarbonate (Boc_2O, 764 mg, 3.5 mmol, 1.1 eq.) was added in one portion. The mixture was stirred at 0 °C for 90 minutes, whereas the pH value was maintained between 9 and 10 through several additions of 1 N aqueous NaOH (in total 1.5 mL, 1.5 mmol, 0.47 eq.). The solvent was removed *in vacuo*, the remaining residue was dissolved in a mixture of ethyl acetate and 5 % aqueous $KHSO_4$ solution. The organic layer was washed thrice with 5 % aqueous $KHSO_4$ and once with brine, then dried over anhydrous $MgSO_4$ and filtered before the solvent was removed *in vacuo*. The compound was used for Fmoc-SPPS without further purification (781 mg as colorless solid, 3.04 mmol, 96 %, MS calcd.: 257.16, m/z found: 258.18 $[M+H]^+$, ^{1}H NMR (500 MHz, DMSO-d_6): δ[ppm] = 11.93 (s, 1H), 6.75 (t, ^3J = 5.6 Hz, 1H), 2.76 (t, ^3J = 6.4 Hz, 2H), 2.09 (tt, $^3J_{ax.-ax.}$ = 12.1 Hz, $^3J_{ax.-eq.}$ = 3.6 Hz, 1H), 1.92 - 1.82 (m, 2H), 1.72 - 1.62 (m, 2H), 1.37 (s, 9H), 1.33-1.17 (m, 3H), 0.86 (qd, $^{2,3}J$ = 13.1 Hz, $^3J_{ax.-eq.}$ = 3.3 Hz, 2H).

3. Synthesis of P1' building blocks and their intermediates

(S)-2-amino-3-(4'-methyl-3'-nitro-[1,1'-biphenyl]-4-yl)propanoic acid x HCl (H-Bpa(4'-Me,3'-NO_2)-OH × HCl or H-Bpa(4'-Me,3'-NO_2)-OH × HCl **(S3)**

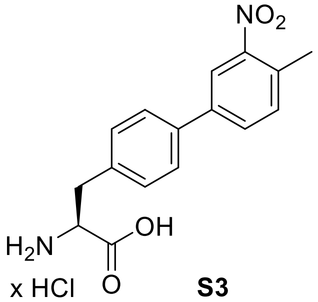

S3

The Suzuki coupling was performed as described for compound **16** in the main manuscript using Boc-Phe(4-Br)-OH (3.000 g, 8.72 mmol, 1 eq.), 4-methyl-3-nitrophenylboronic acid

(1.735 g, 9.59 mmol, 1.1 eq.), Pd(dppf)Cl_2 (48 mg, 0.065 mmol, 0.006 eq.), 2 N aqueous Cs_2CO_3 solution (12.00 mL, 24 mmol, 2.5 eq.) in 150 mL dimethoxyethane with a reaction time of 5 h. The Boc-protected BPA derivative (HPLC method A: 30.02 min) was treated with 15 mL of 4 N HCl in 1,4-dioxane for 1 h at rt. The product was precipitated by addition of cold diethyl ether and washed thrice with cold diethyl ether (2.94 g as pale brown solid, 7.95 mmol, 91 %, HPLC method A: 18.92 min, purity > 95 %, MS calcd.: 300.31, m/z 301.13 $[M+H]^+$, ^{1}H NMR (500 MHz, DMSO-d_6): δ[ppm] = 8.21 (d, ^3J = 2.0 Hz, 1H), 7.95 (dd, ^3J = 7.9 Hz, ^4J = 2.0 Hz, 1H), 7.75 (d, ^3J = 8.4 Hz, 2H), 7.59 (d, ^3J = 8.4 Hz, 1H), 7.40 (d, ^3J = 8.3 Hz, 2H), 4.19 (t, ^3J = 6.5 Hz, 1H), 3.18 (dd, ^2J = 14.3 Hz, ^3J = 6.1 Hz, 1H), 3.12 (dd, ^2J = 14.3 Hz, ^3J = 6.8 Hz, 1H), 2.54 (s, 3H).

(S)-2-((((9H-fluoren-9-yl)methoxy)carbonyl)amino)-3-(4'-methyl-3'-nitro-[1,1'-biphenyl]-4-yl)propanoic acid or Fmoc-Bpa(4’-Me,3’-NO_2)-OH **(S4)**

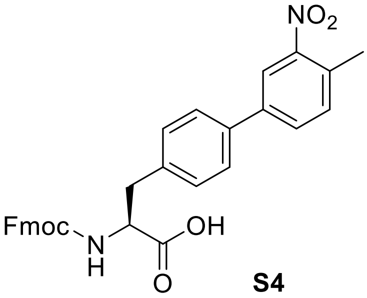

S4

The Fmoc-protecting group was introduced as described in the main manuscript for compound **17** using compound **S3** (2.69 g, 7.98 mmol, 1 eq.), TMSCl (2.00 g, 18.35 mmol, 2.3 eq.), DIPEA (4.6 mL, 26.33 mmol, 3.3 eq.) and 20 mL DCM as well as Fmoc-Cl (1.96 g, 7.58 mmol, 0.95 eq.) in 20 mL DCM. After aqueous workup, the compound was used for Fmoc-SPPS without further purification (3.84 g as pale brown solid, 7.35 mmol, 97 %, HPLC method A: 35.14 min, purity > 97 %, MS calcd.: 522.56, m/z 521.40 $[M-H]^-$.

4-bromo-2-nitro-N-phenylbenzamide **(S5)**

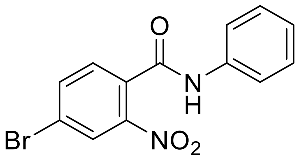

S5

The compound was synthesized as described in the main manuscript for analogue **14** using 4-bromobenzoic acid (2.010 g, 8.17 mmol, 1 eq.), aniline (761 mg, 8.17 mmol, 1eq.), HATU (4.038 g, 10.62 mmol, 1.3 eq.), DIPEA (3.56 mL, 20.43 mmol, 2.5 eq.) and 26 mL DMF. The solvent was removed *in vacuo* and the residue was purified by preparative MPLC (40 g silica gel, cyclohexane / ethyl acetate gradient, start: 0 % ethyl acetate) (2.135 g pale brown solid, 6.65 mmol, 81 %, purity > 95 %). A small sample was purified by preparative HPLC for analytical characterization (HPLC method A: 26.6 min, purity > 99 %, MS calcd.: 319.98, m/z found: 321.04 $[M+H]^+$, ^{1}H NMR (500 MHz, DMSO-d_6): δ[ppm] = 10.66 (s, 1H), 8.37 (d, ^4J = 1.9 Hz, 1H), 8.10 (dd, ^3J = 8.2 Hz, ^4J = 1.9 Hz, 1H), 7.75 (d, ^3J = 8.2 Hz, 1H), 7.64 (dd, ^3J = 8.6 Hz, ^4J = 1.1 Hz, 2H), 7.37 (dd, ^3J = 8.4 Hz, ^4J = 7.5 Hz, 2H), 7.13 (tt, ^3J = 7.7 Hz, ^4J = 1.2 Hz, 1H). ^{13}C NMR (126 MHz, DMSO-D_6): δ[ppm] = 163.03, 147.33, 138.56, 136.56, 131.38, 130.96, 128.79, 126.86, 124.06, 123.02, 119.68.

(S)-2-amino-3-(4'-(isopropylcarbamoyl)-3'-nitro-[1,1'-biphenyl]-4-yl)propanoic acid (H-Bpa(4’-Pha,3’-NO_2)-OH × HCl or H-Bpa(4’-Pha,3’-NO_2)-OH × HCl **(S6)**

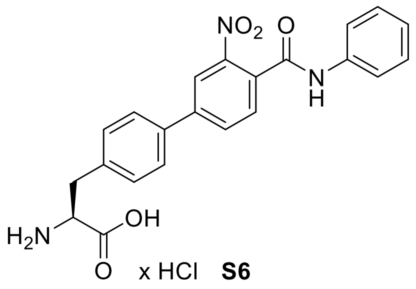

S6

The Miyaura borylation was performed as described in the main manuscript for compound **15** using compound **S5** (2.135 g, 6.65 mmol, 1 eq.), bis(pinakolato)diboron (B_2Pin_2, 1.857 g, 7.3 mmol, 1.1 eq.), Pd(dppf)Cl_2 (163 mg, 0.20 mmol, 0.03 eq.), potassium acetate (1.958 g, 20 mmol, 3 eq.) and 200 mL dimethoxyethane. After a reaction time of 4 h, the mixture was filtrated and a Suzuki coupling was performed without further purification. In a flask equipped with a reflux condenser, the solution of the boronic acid intermediate (HPLC method A: 19.54 min) was treated with Boc-Phe(4-Br)-OH (2.518 g, 7.32 mmol, 1.1 eq.), Pd(dppf)Cl_2 (271 mg, 0.33 mmol, 0.05 eq.) and aqueous 2 N Cs_2CO_3 solution (9.15 mL, 18.3 mmol, 2.5 eq.). After refluxing for 3 h under an argon atmosphere, the mixture was filtered and the solvent was removed *in vacuo*. The crude Boc-protected Bpa-derivative (HPLC method A: 28.25 min) was purified by preparative MPLC (40 g silica gel, DCM / methanol gradient, both solvents

supplemented with 0.1 % (v/v) TFA, start: 0 % methanol). After removal of the solvent *in vacuo*, the dry residue was treated with 6 mL 4 N HCl in 1,4-dioxane at rt for 1 h. The hydrochloride salt **S6** was precipitated from cold diethyl ether and washed thrice with cold diethyl ether (2.244 mg as pale brown solid, 5.07 mmol, 76 %, HPLC method A: 18.6 min, purity > 82 %. A small sample was purified by preparative HPLC for characterization. MS calcd.: 405.13, m/z found: 406.30 $[M+H]^+$, ^1H NMR (500 MHz, DMSO-d_6): δ[ppm] = 10.70 (s, 1H), 8.36 (d, ^4J = 1.8 Hz, 1H), 8.18 (dd, ^3J = 8.0 Hz, ^4J = 1.8 Hz, 1H), 7.87 (d, ^3J = 8.0 Hz, 1H), 7.84 (d, ^3J = 8.3 Hz, 2H), 7.71 - 7.65 (m, 2H), 7.45 (d, ^3J = 8.3 Hz, 2H), 7.42 - 7.29 (m, 2H), 7.14 (tt, ^3J = 7.3 Hz, ^4J = 1.0 Hz, 1H), 4.22 (t, ^3J = 6.5 Hz, 1H), 3.20 (dd, ^2J = 14.3 Hz, ^3J = 6.2 Hz, 1H), 3.15 (dd, ^2J = 14.4 Hz, ^3J = 6.9 Hz, 1H). ^{13}C NMR (126 MHz, DMSO-D_6): δ[ppm] = 170.27, 163.71, 147.53, 142.17, 138.77, 136.12, 135.85, 131.20, 131.02, 130.40, 129.95, 128.78, 127.16, 123.96, 121.70, 119.67, 53.15, 35.53.

(S)-2-((((9H-fluoren-9-yl)methoxy)carbonyl)amino)-3-(3'-nitro-4'-(phenylcarbamoyl)-[1,1'-biphenyl]-4-yl)propanoic acid or Fmoc-Bpa(4'-Pha,3'-NO_2)-OH **(S7)**

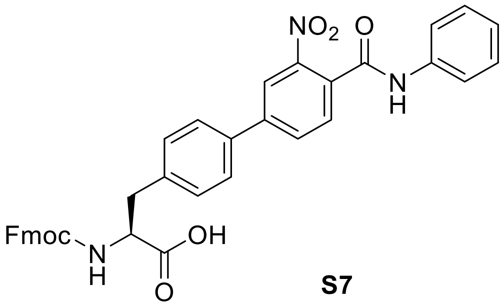

S7

The Fmoc-protecting group was introduced as described in the main manuscript for compound **17** using compound **S6** (4.06 g, 9.19 mmol, 1 eq.), chlorotrimethylsilane (TMSCl, 2.20 g, 20.23 mmol, 2.2 eq.), DIPEA (5.12 mL, 29.41 mmol, 3.2 eq.) and 40 mL DCM as well as FmocCl (2.30 mg, 8.91 mmol, 0.97 eq.) in 20 mL DCM. After aqueous workup, the crude product was purified by preparative MPLC (40 g silica gel, DCM / methanol, both solvents supplemented with 0.1 % (v/v) TFA, start at 0 % methanol). (3.346 g pale brown solid, 7.57 mmol, 85%, HPLC method A: 32.36 min, purity > 96 %, MS calcd.: 627.20, m/z found: 628.14 $[M+H]^+$.

4. Synthesis of P2' building blocks

(1r,4r)-4-(((((9H-fluoren-9-yl)methoxy)carbonyl)amino)methyl)cyclohexane-1-carboxylic acid or Fmoc-Txa-OH **(S8)**

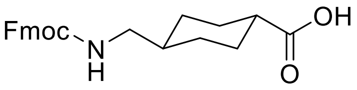

S8

The Fmoc-protecting group was introduced as described for intermediate **17** in the main manuscript using tranexamic acid (350 mg, 2.22 mmol, 1 eq.), chlorotrimethylsilane (TMSCl, 556 mg, 5.11 mmol, 2.3 eq.), DIPEA (1.24 mL, 7.10 mmol, 3.2 eq.) and 10 mL DCM as well as Fmoc-Cl (547 mg, 2.12 mmol, 0.95 eq.) dissolved in 5 mL DCM. After aqueous workup, the compound was used for Fmoc-SPPS without further purification (715 mg colorless solid, 1.88 mmol, 85 %, HPLC method A: 28.92 min, purity > 99 %, MS calcd.: 379.18, m/z found: 380.22 $[M+H]^+$.

2-(3-((((9H-fluoren-9-yl)methoxy)carbonyl)amino)phenyl)acetic acid (Fmoc-3-aminophenylacetic acid or Fmoc-3-aPhac-OH **(S9)**

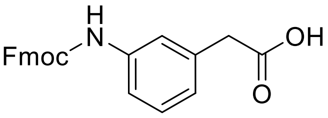

S9

The Fmoc-protecting group was introduced as described in the main manuscript for compound **17** using 2-(3-aminophenyl)acetic acid (2.00 g, 13.2 mmol, 1.0 eq.), chlorotrimethylsilane (3.15 g, 29.0 mmol, 2.2 eq.) and DIPEA (5.05 mL, 29.0 mmol, 2.2 eq.) in 33 mL DCM as well as Fmoc-Cl (3.25 g, 12.5 mmol, 0.95 eq.) dissolved in 8 mL DCM. After aqueous workup, the compound was used for the Fmoc-SPPS without further purification (4.54 g colorless solid, 12.2 mmol, 97 %, HPLC: method A: 29.53 min, purity > 98 %, MS calcd.: 373.13, m/z found: 374.14 $[M+H]^+$, ^{1}H NMR (500 MHz, DMSO-d_6): δ[ppm] = 12.28 (bs, 1H), 9.69 (s, 1H), 7.91 (d, ^3J = 7.5 Hz, 2H), 7.76 (d, ^3J = 7.5 Hz, 2H), 7.50 - 7.29 (m, 6H), 7.20 (t, ^3J = 7.9 Hz, 1H), 6.89 (d, ^3J = 7.5 Hz, 1H), 4.46 (d, ^3J = 6.8 Hz, 2H), 4.31 (t, ^3J = 6.8 Hz, 1H), 3.50 (s, 2H).

2-(4-((((9H-Fluoren-9-yl)methoxy)carbonyl)amino)phenyl)acetic acid or Fmoc-4-aPhac-OH **(S10)**

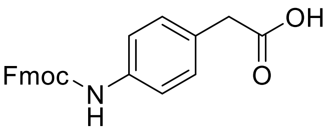

S10

The Fmoc-protecting group was introduced as described in the main manuscript for compound **17** using 2-(4-aminophenyl)acetic acid (1.84 g, 12.2 mmol, 1.0 eq.), chlorotrimethylsilane (3.31 g, 30.5 mmol, 2.5 eq.), DIPEA (5.31 mL, 30.5 mmol, 2.5 eq.) and 33 mL DCM as well as Fmoc-Cl (3.06 g, 11.8 mmol, 0.97 eq.) dissolved in 20 mL DCM. After aqueous workup, the compound was used for Fmoc-SPPS without further purification (4.29 g pale yellow solid, 11.5 mmol, 97 %, HPLC method A: 29.10 min, purity > 97 %, MS calcd.: 373.13, m/z found: 374.18 $[M+H]^+$, ^1H NMR (500 MHz, DMSO-d_6): δ[ppm] = 12.24 (s, 1H), 9.64 (s, 1H), 7.91 (d, ^3J = 7.5 Hz, 2H), 7.75 (d, ^3J = 7.4 Hz, 2H), 7.49 - 7.27 (m, 6H), 7.15 (d, ^3J = 8.4 Hz, 2H), 4.48 (d, ^3J = 6.6 Hz, 2H), 4.31 (t, ^3J = 6.7 Hz, 1H), 3.48 (s, 2H).

6-((((9H-fluoren-9-yl)methoxy)carbonyl)amino)-2-naphthoic acid (Fmoc-6-amino-2-naphthoic acid, Fmoc-6-aNap-OH **(S11)**

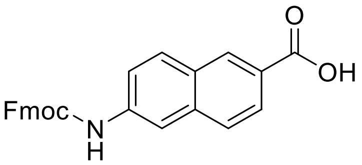

S11

The Fmoc-protecting group was introduced as described in the main manuscript for compound **17** using 6-amino-2-naphthoic acid (500 mg, 2.67 mmol, 1 eq.), chlorotrimethylsilane (639 mg, 5.88 mmol, 2.2 eq.), DIPEA (1.00 mL, 5.88 mmol, 2.2 eq.) and 12 mL DCM as well as Fmoc-Cl (663 mg, 2.56 mmol, 0.96 eq.) dissolved in 3 mL DCM. After aqueous workup, the compound was used for Fmoc-SPPS without further purification (994 mg pale brown solid, 0.243 mmol, 95%, HPLC method B: start at 10 % B, 32.85 min, purity > 98 %. MS calcd.: 409.44, m/z found: 410.13 $[M+H]^+$, ^1H NMR (500 MHz, DMSO-d_6): δ[ppm] = 12.87 (bs, 1H), 10.06 (s, 1H), 8.49 (s, 1H), 8.12 (s, 1H), 8.01 (d, ^3J = 8.9 Hz, 1H), 7.96 - 7.89 (m, 3H), 7.83 (d, ^3J = 8.7 Hz, 1H), 7.78 (d, ^3J = 7.5 Hz, 2H), 7.61 (pd, ^3J = 7.7 Hz, 1H), 7.44 (t, ^3J = 7.4 Hz, 2H), 7.39 - 7.31 (m, 2H), 4.56 (d, ^3J = 6.3 Hz, 2H), 4.35 (t, ^3J = 6.4 Hz, 1H).

5. Synthesis of building blocks and intermediates for linker segment

(9H-fluoren-9-yl)methyl piperazine-1-carboxylate x HCl or Fmoc-Pip-H × HCl **(S12)**

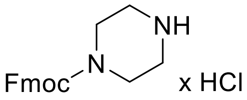

S12

Fmoc-Cl (695 mg, 2.68 mmol, 1 eq.) and Boc-piperazine (1.00 g, 5.37 mmol, 2 eq.) were dissolved in 20 mL DCM, which was previously cooled to at 0 °C in an ice bath. The solution was stirred at 0 °C - rt overnight. Then, the mixture was washed thrice with 5 % aqueous $KHSO_4$ solution, thrice with saturated aqueous $NaHCO_3$ solution and once with brine, dried over anhydrous $MgSO_4$ and filtered, before the solvent was removed *in vacuo*. The remaining residue was treated with 7 mL of 4 N HCl in 1,4-dioxane for 1 h at room temeprature and the product was precipitated from cold diethyl ether. After washing thrice with cold diethyl ether, compound **12** was dried in vacuo and used without further purification (722 mg as colorless solid, 2.09 mmol, 78 %, HPLC method A: 18.34 min, purity > 99 %, MS calcd.: 308.15, m/z found: 309.21 $[M+H]^+$, ^{1}H NMR (500 MHz, DMSO-*d*$_6$): δ[ppm] = 7.91 - 7.89 (m, 2H), 7.64 (dd, ^3J = 7.4 Hz, ^4J = 0.9 Hz, 2H), 7.45 - 7.39 (m, 2H), 7.35 (td, ^3J = 7.4 Hz, ^4J = 1.2 Hz, 2H), 4.40 (d, ^3J = 6.6 Hz, 2H), 4.29 (t, ^3J = 6.5 Hz, 1H), 3.55 (t, ^3J = 5.5 Hz, 4H), 3.01 (t, ^3J = 5.1, 4.0 Hz, 4H). ^{13}C NMR (126 MHz, DMSO-d$_6$): δ[ppm] = 154.07, 143.66, 140.70, 127.66, 127.11, 124.99, 120.09, 66.93, 46.65, 42.11, 40.21.

2-(4-(((9H-fluoren-9-yl)methoxy)carbonyl)piperazin-1-yl)acetic acid × TFA or Fmoc-Pip-Ac-OH × TFA **(S13)**

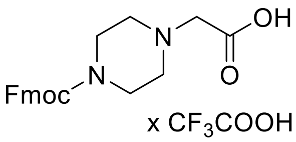

S13

A mixture of compound **S12** (350 mg, 1.02 mmol, 1 eq.), DIPEA (1.07 mL, 6.12 mmol, 6 eq.) and 5 mL DCM was cooled to 0 °C. Bromoacetic acid (425 mg, 3.06 mmol, 3 eq.) was added in one portion and the mixture was stirred in an ice bath at 0 °C and at room temperature overnight. The solution was acidified with TFA and the solvent removed *in vacuo*. The product was purified by preparative HPLC (239 mg colorless lyophilized solid, 0.50 mmol, 49 %,

HPLC method A: 19.14 min, purity > 95 %, MS calcd.: 366.42, m/z found: 367.16 $[M+H]^+$. ^1H NMR (500 MHz, DMSO-d_6): δ[ppm] = 7.90 (d, ^3J = 7.5 Hz, 2H), 7.64 (dd, ^3J = 7.4 Hz, ^4J = 0.9 Hz, 2H), 7.46 - 7.41 (m, 2H), 7.35 (td, ^3J = 7.4 Hz, ^4J = 1.2 Hz, 2H), 4.38 (d, ^3J = 6.7 Hz, 2H), 4.29 (t, ^3J = 6.6 Hz, 1H), 3.97 (s, 2H), 3.58 (bs, 4H), 3.13 (bs, 4H). ^{13}C NMR (126 MHz, DMSO-D_6): δ[ppm] = 157.74, 154.03, 143.63, 140.71, 127.68, 127.13, 125.00, 120.12, 67.04, 55.66, 51.24, 51.24, 46.61. One expected aliphatic signal was not resolved, - probably due to the flexibility of the aliphatic ring.

3-(4-(((9H-fluoren-9-yl)methoxy)carbonyl)piperazin-1-yl)propanoic acid × TFA (Fmoc-Pip-Prop-OH × TFA **(S14)**

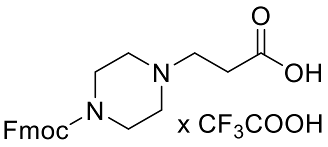

S14

The compound was synthesized analogously to derivative **S13** using compound **S12** (350 mg, 1.02 mmol, 1 eq.), 3-bromopropanoic acid (468 mg, 3.06 mmol, 3 eq.), DIPEA (1.07 mL, 6.12 mmol, 6 eq.) and 5 mL DCM (305 mg colorless solid, 0.62 mmol, 60 %, HPLC method A: 20.74 min, purity > 95 %, MS calcd.: 380.17, m/z found: 381.11 $[M+H]^+$, ^1H NMR (500 MHz, DMSO-d_6): δ[ppm] = 12.44 (bs, 1H), 9.95 (bs, 1H),7.91 (dt, ^3J = 7.6 Hz, ^4J = 0.8 Hz, 2H), 7.69 - 7.59 (m, 2H), 7.48 - 7.40 (m, 2H), 7.35 (td, ^3J = 7.4 Hz, ^4J = 1.2 Hz, 2H), 4.41 (d, ^3J = 6.6 Hz, 2H), 4.29 (t, ^3J = 6.4 Hz, 1H), 3.77 (br), 3.30 (t, ^3J = 7.4 Hz, 2H), 3.17 (bs, 4H), 2.74 (t, ^3J = 7.4 Hz, 2H). ^{13}C NMR (126 MHz, DMSO-D_6): δ[ppm] = 171.44, 153.97, 143.63, 140.72, 127.68, 127.12, 124.99, 120.12, 67.02, 51.47, 50.66, 46.63, 28.55. One expected aliphatic signal was not resolved, - probably due to the flexibility of the aliphatic ring.

Benzyl 4-bromobutanoate **(S15)**

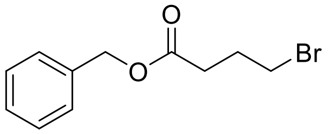

S15

The compound was synthesized as described before.[12]

4-(4-(((9H-fluoren-9-yl)methoxy)carbonyl)piperazin-1-yl)butanoic acid × 2 TFA or Pip-But-Bn × 2 TFA **(S16)**

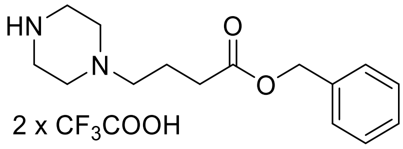

S16

Boc-Piperazine (188 mg, 1.01 mmol, 1.3 eq.) and triethylamine (158 µL, 1.56 mmol, 2.0 eq.) were suspended in 2 mL abs. THF in a 10 mL microwave reaction tube. A solution of compound **S15** (200 mg, 0.78 mmol, 1.0 eq.) in 1 mL abs. THF was added in one portion and the mixture was stirred in a laboratory microwave (Discover SP microwave, CEM GmbH, Kamp-Lintfort, Germany) at 80 °C for 8 h and subsequently outside the microwave at rt overnight. The solvent was removed *in vacuo* and the residue was treated with 4 mL 4 N HCl in 1,4-dioxane for 1 h at room temperature. The HCl salt was precipitated from cold diethyl ether and purified by preparative HPLC (275 mg colorless solid, 0.56 mmol, 72 %, HPLC method A: 11.99 min, purity > 98 %, MS calcd.: 262.17, m/z found: 263.13 $[M+H]^+$, ^{1}H NMR (500 MHz, DMSO-d_6): δ[ppm] = 9.17 (s, 2H), 7.41 - 7.31 (m, 5H), 5.11 (s, 2H), 3.31 (bs, 4H), 3.20 (s, 4H), 2.96 (bs, 2H), 2.46 (t, ^3J = 7.3 Hz, 2H), 1.87 (p, ^3J = 7.5 Hz, 2H). ^{13}C NMR (126 MHz, DMSO-D_6): δ[ppm] = 171.96, 136.05, 128.39, 128.01, 127.92, 65.57, 55.29, 48.35, 40.90, 30.46. Not all expected aliphatic signals could be resolved, probably due to the flexibility of the aliphatic ring.

6. Synthesis of inhibitors of first series (Table 1 in main manuscript)

The provided inhibitor numbers correspond to the numbering used in the main manuscript. Precursors are labeled with the respective inhibitor number and additional suffixes.

Inhibitor 3

(1r,4s)-4-(Aminomethyl)-N-((S)-3,7,10-trioxo-2,6,9-triaza-1(1,4)-benzenacyclododecaphane-11-yl)cyclohexane-1-carboxamide × TFA
or H-Txa-Phe(*c*[4-NH)-Gly-βAla] × TFA

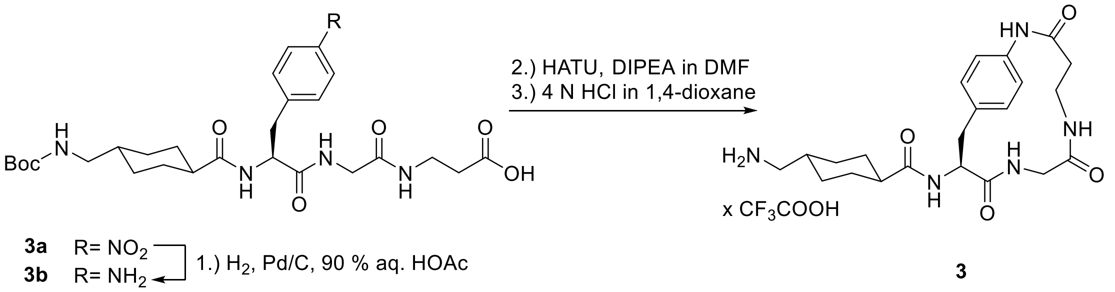

The linear precursor was synthesized on 250 mg 2-CTC resin (0.4 mmol absolute) as described for inhibitor **12** in the main manuscript using commercially available Fmoc-βAla-OH, Fmoc-Gly-OH, Fmoc-Phe(4-NO_2)-OH and Boc-Txa-OH (**S2**). After mild acidic cleavage from resin (cleavage solution neutralized after each cleavage step) and hydrogenation of the crude peptide **3a** (HPLC method A: 21.67 min, MS calcd.: 577.27, m/z found: 578.33 $[M+H]^+$) the amine **3b** was purified by preparative HPLC (121 mg, 0.22 mmol colorless solid, HPLC Method A: 13.81 min, purity > 85 %, MS calcd.: 547.30, m/z found: 548.06 $[M+H]^+$). Cyclization of **3b** provided the Boc-protected intermediate **3c** (structure not shown, HPLC method A: 17.52 min, MS calcd.: 529.29, m/z found: 530.23 $[M+H]^+$), which was dried *in vacuo*. The residue was treated with 5 mL 20 % (v/v) TFA in DCM for 1 h at room temperature, the crude product was precipitated with cold diethyl ether and purified by preparative HPLC (21 mg colorless lyophilizate, 0.039 mmol, 18 % over two steps for cyclization and Boc removal, HPLC method B: 16.28 min, start at 1 % B, purity > 98 %, MS calcd.: 429.24, m/z found: 430.20 $[M+H]^+$, ^{1}H NMR (500 MHz, DMSO-d_6): δ[ppm] = 9.34 (s, 1H), 8.11 (d, ^3J = 7.2 Hz, 1H), 7.73 (s, 3H), 7.42 – 7.34 (m, 1H), 7.32 (dd, ^3J = 8.2, ^4J = 2.0 Hz, 1H), 7.20 (dd, ^3J = 8.1, ^4J = 2.2 Hz, 1H), 7.07 (dd, ^3J = 8.1, ^4J = 2.2 Hz, 1H), 6.98 (dd, ^3J = 8.1, ^4J = 2.0 Hz, 1H), 6.06 (t, ^3J = 5.8 Hz, 1H), 4.48 – 4.37 (m, 1H), 3.59 (dd, ^2J = 16.0, ^3J = 7.9 Hz, 1H), 3.25 – 3.17 (m, 1H), 3.08 – 2.97

(m, 3H), 2.73 – 2.62 (m, 3H), 2.64 – 2.57 (m, 1H), 2.34 – 2.28 (m, 1H), 2.19 (tt, $^3J_{ax.-ax.}$ = 11.9, $^3J_{ax.-eq.}$ = 3.3 Hz, 1H), 1.84 – 1.69 (m, 4H), 1.57 – 1.44 (m, 1H), 1.40 – 1.23 (m, 2H), 1.01 – 0.87 (m, 2H). ^{13}C NMR (126 MHz, DMSO-D_6): δ[ppm] = 174.68, 173.68, 170.30, 167.71, 136.64, 134.99, 130.33, 129.83, 54.89, 44.33, 42.93, 42.05, 37.63, 35.67, 35.04, 34.26, 28.85, 28.28.

Inhibitor 4

(1r,4S)-4-(aminomethyl)-N-((8S,11S)-8-(3-guanidinopropyl)-3,7,10-trioxo-2,6,9-triaza-1(1,4)-benzenacyclododecaphane-11-yl)cyclohexane-1-carboxamide × 2 TFA
or H-Txa-Phe(c[4-NH)-Arg-βAla] × 2 TFA

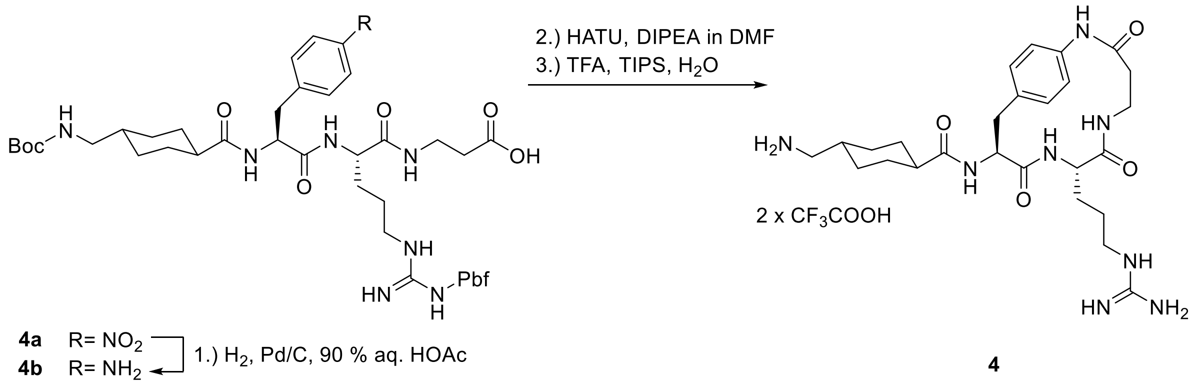

Inhibitor **4** was prepared with minor modifications as described for inhibitor **12** in the main manuscript and inhibitor **3** above using Fmoc-βAla-OH, Fmoc-Arg(Pbf)-OH, Fmoc-Phe(4-NO_2)-OH, and Boc-Txa-OH (**S2**) for SPPS on 250 mg 2-CTC resin (0.4 mmol absolute). After mild acidic cleavage from resin (cleavage solution neutralized after each cleavage step), intermediate **4a** (HPLC method A: 28.87 min, MS calcd.: 928.44, m/z found: 929.43 $[M+H]^+$) was hydrogenated. Purification by preparative MPLC (12 g silica gel, DCM/methanol gradient, both solvents supplemented with 1 % (v/v) formic acid, start at 0 % methanol) yielded intermediate **4b** (225 mg, 0.25 mmol brown solid, HPLC: method A: 21.82 min, purity > 80 %, MS calcd.: 898.46, m/z found: 899.35 $[M+H]^+$). Cyclization provided intermediate **4c** (structure not shown, HPLC method A: 25.70 min), which was treated with 3 mL of a mixture of TFA, water and triisopropyl silane (95/2.5/2.5, v/v/v) at rt for 60 min. Precipitation in cold diethyl ether and purification by preparative HPLC provided inhibitor **4** (37 mg colorless lyophilizate, 0.049 mmol, 20 % for cyclization and deprotection, HPLC method B: 16.00 min, start at 1 % B, purity > 98 %, MS calcd.: 528.32, m/z found: 529.28 $[M+H]^+$.

Inhibitor 5

(1r,4S)-4-(aminomethyl)-N-((S)-3,8,15-trioxo-2,7,14-triaza-1(1,3),6(1,4)-dibenzenacyclohexadecaphane-4-yl)cyclohexane-1-carboxamide × TFA
or H-Txa-Phe(*c*[4-NH)-3-aPhac-Aca] × TFA

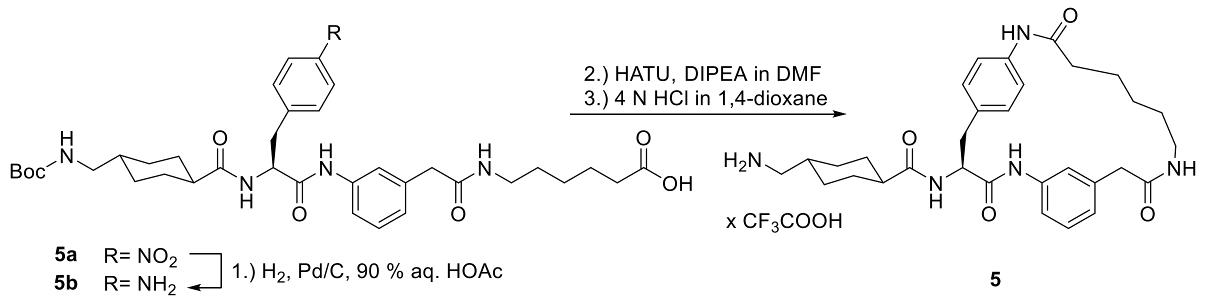

Inhibitor **5** was prepared as described above for inhibitor **3** using Fmoc-6-aminohexanoic acid, Fmoc-3-aminophenylacetic acid **(S9)**, Fmoc-Phe(4-NO_2)-OH, and Boc-Txa-OH (**S2**) for SPPS on 250 mg 2-CTC-resin (0.4 mmol absolute). After mild acidic cleavage (cleavage solution neutralized after each cleavage step), intermediate **5a** (HPLC method A: 26.14 min, MS calcd.: 695.35, m/z found: 696.41 $[M+H]^+$) was hydrogenated to compound **5b**, which was purified by MPLC (12 g silica gel, DCM/methanol gradient, both solvents supplemented with 1 % (v/v) formic acid, start at 0 % methanol). Intermediate **5b** (229 mg, 0.34 mmol pale brown solid, HPLC method A: 18.19 min, purity > 88 %, MS calcd.: 665.38, m/z found: 666.41 $[M+H]^+$) was cyclized providing the Boc-protected derivative **5c** (structure not shown, HPLC method A: 23.77 min, MS calcd.: 647.37, m/z found: 648.15 $[M+H]^+$), which was dried *in vacuo* and deprotected with 5 mL 20 % (v/v) TFA in DCM for 1 h at room temperature. Inhibitor **5** was precipitated from cold diethyl ether and purified by preparative HPLC (37 mg colorless solid, 0.063 mmol, 19 % for cyclization and deprotection, HPLC method B: 16.28 min, start at 1 % B, purity > 98 %, MS calcd.: 547.32, m/z found: 548.38 $[M+H]^+$, ^{1}H NMR (500 MHz, DMSO-d_6): δ[ppm] = 9.70 (s, 1H), 9.35 (s, 1H), 8.06 (d, ^3J = 7.4 Hz, 1H), 7.72 (bs, 3H), 7.70 – 7.64 (m, 2H), 7.43 (d, ^3J = 8.2 Hz, 2H), 7.13 (t, ^3J = 7.9 Hz, 1H), 7.04 (d, ^3J = 8.4 Hz, 2H), 6.96 (d, ^3J = 7.7 Hz, 1H), 6.48 (s, 1H), 4.55 (ddd, ^3J = 12.4 Hz, 7.2, 5.2 Hz, 1H), 3.22 (d, ^3J = 3.9 Hz, 2H), 3.13 – 3.04 (m, 1H), 2.98 – 2.85 (m, 2H), 2.79 – 2.67 (m, 1H), 2.68 (p, ^3J = 5.9 Hz, 2H), 2.29 – 2.17 (m, 3H), 1.87 – 1.71 (m, 4H), 1.67 – 1.56 (m, 2H), 1.55 – 1.46 (m, 1H), 1.42 – 1.26 (m, 4H), 1.25 – 1.11 (m, 2H), 0.96 1.02 – 0.90 (m, 2H). ^{13}C NMR (126 MHz, DMSO-D_6): δ[ppm] = 175.15, 171.73, 170.11, 167.10, 158.57, 138.59, 138.16, 136.85, 129.85, 129.84,

128.58, 125.73, 119.20, 118.36, 55.60, 44.94, 43.54, 38.88, 37.15, 35.65, 29.46, 29.43, 29.30, 28.92, 28.87, 26.61, 25.67.

Inhibitor 6

(1r,4S)-4-(aminomethyl)-N-((S)-3,8,15-trioxo-2,7,14-triaza-1,6(1,4)-dibenzenacyclohexadecaphane-4-yl)cyclohexane-1-carboxamide × TFA
or H-Txa-Phe(c[4-NH)-4-aPhac-Aca] × TFA

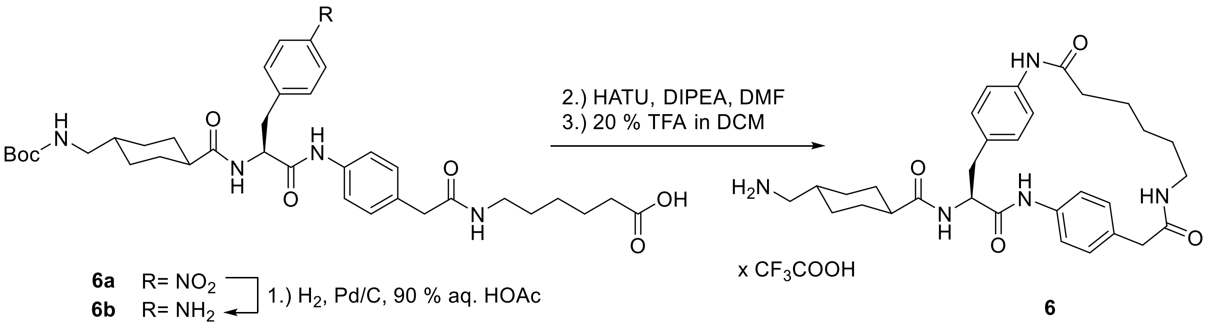

The linear precursor was synthesized on 250 mg 2-CTC resin (0.4 mmol absolute) using Fmoc-6-aminohexanoic acid, Fmoc-4-aPhac-OH **(S10)**, Fmoc-Phe(4-NO_2)-OH, and Boc-Txa-OH **(S2)**. After mild acidic cleavage from the resin, compound **6a** (HPLC method A: 26.44 min, MS calcd.: 695.35, m/z found: 696.56 $[M+H]^+$) was hydrogenated to obtain the amine intermediate **6b**, which was purified by flash chromatography (12g silica gel, methanol/DCM incl. 1 % HCOOH, start at 0 % methanol) (80 mg, 0.12 mmol colorless solid, HPLC method A: 17.88 min, purity > 95 %, MS calcd.: 665.38, m/z found: 666.12 $[M+H]^+$). Compound **6b** was cyclized as described before providing the Boc-derivative **6c** (structure not shown, HPLC method A: 22.71 min, MS (ESI, positive: calcd. 647.37, m/z 648.34 $[M+H]^+$). Intermediate **6c** was treated with 5 mL 20 % (v/v) TFA in DCM for 1 h at room temperature and precipitated with cold diethyl ether. The residue was purified by preparative HPLC providing inhibitor **6** (8 mg colorless lyophilized solid, 0.012 mmol, 10 %, HPLC method B: 15.87 min, start at 10 % B, purity > 98 %, MS calcd.: 547.32, m/z found: 548.38 $[M+H]^+$, ^{1}H NMR (500 MHz, DMSO-d_6): δ[ppm] = 9.61 (s, 1H), 9.09 (s, 1H), 8.11 (d, ^3J = 7.5 Hz, 1H), 7.73 (t, ^3J = 5.5 Hz, 1H), 7.67 (s, 3H), 7.36 (d, ^3J = 8.4 Hz, 2H), 7.04 (d, ^3J = 8.5 Hz, 2H), 6.96 (d, ^3J = 8.3 Hz, 2H), 6.82 (d, ^3J = 8.3 Hz, 2H), 4.61 – 4.54 (m, 1H), 3.21 (s, 2H), 3.19 – 3.09 (m, 1H), 3.07 – 2.94 (m, 2H), 2.74 – 2.57 (m, 3H), 2.30 – 2.17 (m, 3H), 1.87 – 1.72 (m, 4H), 1.67 – 1.40 (m, 3H), 1.42 – 1.20 (m, 4H), 1.17 – 1.05 (m, 2H), 1.04 – 0.90 (m, 2H).

Inhibitor 7

(1r,4S)-4-(aminomethyl)-N-((S)-3,8,14-trioxo-2,7,13-triaza-1,6(1,4)-dibenzenacyclopentadecaphane-4-yl)cyclohexane-1-carboxamide × TFA
or H-Txa-Phe(c[4-NH)-4-aPhac-Ava] × TFA

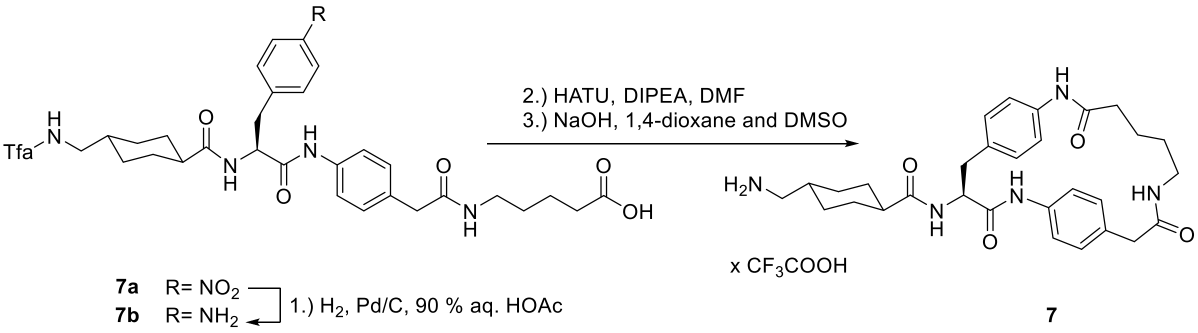

The synthesis was performed as described for inhibitor **12** in the main manuscript. The linear precursor was synthesized on 250 mg 2-CTC resin (0.4 mmol absolute) using Fmoc-5-aminopentanoic acid, Fmoc-4-aPhac-OH **(S10)**, Fmoc-Phe(4-NO_2)-OH, and Tfa-Txa-OH **(S1)**. After mild acidic cleavage from the resin, the crude compound **7a** (HPLC method A: 23.24 min, MS calcd.: 677.27, m/z found: 676.49 $[M-H]^-$) was hydrogenated and purified by preparative HPLC providing intermediate **7b** (201 mg, 0.31 mmol colorless solid, HPLC method A: 14.84 min, purity > 99 %, MS calcd.: 647.29, m/z found: 648.29 $[M+H]^+$, ^1H NMR (500 MHz, DMSO-d_6): δ[ppm] = 11.94 (br, 1H), 9.98 (s, 1H), 9.35 (t, ^3J = 5.8 Hz, 1H), 8.00 (d, ^3J = 8.3 Hz, 1H), 7.95 (t, ^3J = 5.6 Hz, 1H), 7.47 (d, ^3J = 8.6 Hz, 2H), 7.24 (d, ^3J = 8.3 Hz, 2H), 7.16 (d, ^3J = 8.6 Hz, 2H), 7.02 (d, ^3J = 8.2 Hz, 2H), 4.61 (td, $^{2,3}J$ = 8.7 Hz, 5.4 Hz, 1H), 3.33 (s, 2H), 3.08 – 2.93 (m, 5H), 2.82 (dd, ^2J = 13.6 Hz, ^3J = 9.4 Hz, 1H), 2.20 (t, ^3J = 7.3 Hz, 2H), 2.11 (tt, $^3J_{ax.-ax.}$ = 12.0 Hz, $^3J_{ax.-eq.}$ 3.3 Hz, 1H), 1.73 – 1.54 (m, 4H), 1.53 – 1.36 (m, 5H), 1.30 – 1.11 (m, 2H), 0.99 – 0.80 (m, 2H).

Compound **7b** was cyclized as described before. Afterwards, the solvent was removed and the remaining Tfa-protected derivative **7c** (HPLC method A: 18.53 min) was treated with a mixture of 1 N aq. NaOH, 1,4-dioxane and DMSO as solvent for 1 h at rt to cleave the Tfa-protecting group. The final inhibitor **7** was purified by preparative HPLC (32 mg colorless solid, 0.05 mmol, 16 %, HPLC method B: 13.13 min, start at 10 % B, purity > 99 %, MS calcd.: 533.30, m/z found: 534.35 $[M+H]^+$, ^1H NMR (500 MHz, DMSO-d_6): δ[ppm] = 9.54 (s, 1H), 9.10 (s, 1H), 8.12 (d, ^3J = 7.4 Hz, 1H), 7.84 (t, ^3J = 6.2 Hz, 1H), 7.68 (bs, 3H), 7.26 (d, ^3J = 8.7 Hz, 1H), 7.05 – 6.97 (m, 4H), 6.92 (d, J = 8.5 Hz, 2H), 4.57 (dt, ^2J = 11.3 Hz, ^3J = 7.0 Hz, 1H), 3.20 (d, ^3J = 12.4 Hz, 1H), 3.17 – 2.99 (m, 2H), 2.90 – 2.77 (m, 1H), 2.68 (p, ^3J = 5.8 Hz,

2H), 2.65 – 2.58 (m, 1H), 2.23 (tt, $^3J_{ax.-ax.}$ = 12.0 Hz, $^3J_{ax.-eq.}$ = 3.3 Hz, 1H), 2.20 – 2.13 (m, 1H), 2.12 – 2.05 (m, 2H), 1.84 – 1.74 (m, 4H), 1.58 – 1.48 (m, 1H), 1.48 – 1.39 (m, 2H), 1.39 – 1.31 (m, 2H), 1.30 – 1.14 (m, 2H), 1.14 – 1.02 (m, 1H), 0.97 (qd, ^2J and $^3J_{ax.-ax}$ = 13.0 Hz, $^3J_{ax.-eq.}$ = 3.5 Hz, 2H).

Inhibitor 8

(1r,4S)-4-(aminomethyl)-N-((S)-3,8,12,15-tetraoxo-2,7,11,14-tetraaza-1,6(1,4)-dibenzenacyclohexadecaphane-4-yl)cyclohexane-1-carboxamide × TFA
or H-Txa-Phe(*c*[4-NH)-4-aPhac-Gly-βAla] × TFA

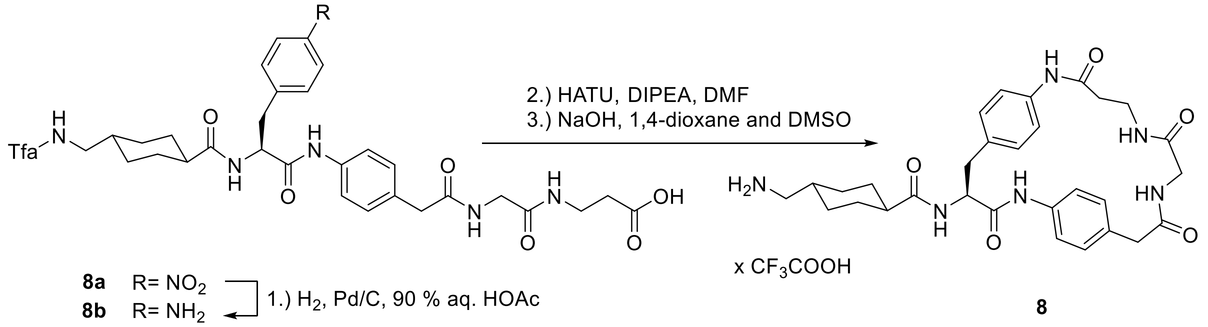

The synthesis was performed as described for inhibitor **12** in the main manuscript. The linear precursor was synthesized on 250 mg 2-CTC resin (0.4 mmol absolute) using Fmoc-β-Ala-OH, Fmoc-Gly-OH, Fmoc-4-aPhac-OH **(S10)**, Fmoc-Phe(4-NO_2)-OH, and Tfa-Txa-OH **(S1)**. After mild acidic cleavage from resin, the crude compound **8a** (HPLC method A: 21.04 min, MS calcd.: 706.26, m/z found 705.33 $[M-H]^-$) was hydrogenated and the amine was purified by preparative HPLC yielding intermediate **8b** (140 mg colorless solid, 0.21 mmol, HPLC method A: 12.86 min, purity > 99 %, MS calcd.: 676.28, m/z found: 677.27 $[M+H]^+$, ^{1}H NMR (500 MHz, DMSO-d_6): δ[ppm] = 12.21 (br, 1H), 9.99 (s, 1H), 9.36 (t, ^3J = 5.9 Hz, 1H), 8.15 (t, ^3J = 5.8 Hz, 1H), 8.02 (d, ^3J = 8.3 Hz, 1H), 7.90 (t, ^3J = 5.6 Hz, 1H), 7.47 (d, ^3J = 8.6 Hz, 2H), 7.26 (d, ^3J = 8.4 Hz, 2H), 7.19 (d, ^3J = 8.6 Hz, 2H), 7.05 (d, ^3J = 8.3 Hz, 2H), 4.62 (td, $^{2,3}J$ = 8.6, 5.5 Hz, 1H), 3.65 (d, ^3J = 5.8 Hz, 2H), 3.42 (s, 2H), 3.26 (pq, J = 6.9 Hz, 2H), 3.06 – 2.96 (m, 3H), 2.83 (dd, ^2J = 13.7 Hz, ^3J = 9.4 Hz, 1H), 2.38 (t, ^3J = 7.0 Hz, 2H), 2.12 (tt, $^3J_{ax.-ax.}$ = 12.0 Hz, $^3J_{ax.-eq.}$3.2 Hz, 1H), 1.75 – 1.56 (m, 4H), 1.52 – 1.38 (m, 1H), 1.30 – 1.11 (m, 2H), 0.98 – 0.82 (m, 2H).

Compound **8b** was cyclized as described before. Afterwards, the solvent was removed and the remaining Tfa-protected derivative **8c** (structure not shown, HPLC method A: 17.19 min) was

treated with a mixture of 1 N aq. NaOH, 1,4-dioxane and DMSO as solvent for 1 h at rt to cleave the Tfa-protecting group. The final inhibitor **8** was purified by preparative HPLC (35 mg colorless solid, 0.05 mmol, 25 %, HPLC method B: 19.76 min, start at 1 % B, purity > 97 %, MS calcd.: 562.29, m/z found: 563.18 $[M+H]^+$, ^{1}H NMR (500 MHz, DMSO-d_6): δ[ppn]= 9.63 (s, 1H), 9.06 (s, 1H), 8.08 (d, ^3J = 7.4 Hz, 1H), 7.92 (t, ^3J = 5.5 Hz, 1H), 7.65 (bs, 3H), 7.28 (t, ^3J = 4.8 Hz, 1H), 7.22 (d, ^3J = 8.0 Hz, 2H), 6.99 (d, ^3J = 8.3 Hz, 2H), 6.96 (d, ^3J = 8.3 Hz, 2H), 6.85 (d, ^3J = 8.1 Hz, 2H), 4.51 (dt, ^2J = 11.3 Hz, ^3J = 7.0 Hz, 1H), 3.63 (d, ^3J = 5.6 Hz, 2H), 3.31 (d, J = 14.1 Hz, 2H), 3.26 (d, J = 14.0 Hz, 1H), 3.00 (dd, ^2J = 12.9 Hz, ^3J = 6.5 Hz, 1H), 2.70 – 2.55 (m, 3H), 2.39 – 2.30 (m, 2H), 2.21 (tt, $^3J_{ax.-ax.}$ = 11.1 Hz, $^3J_{ax.-eq.}$ 2.7 Hz, 1H), 1.83 – 1.71 (m, 4H), 1.55 – 1.42 (m, 1H), 1.40 – 1.23 (m, 2H), 0.94 (qd, J = $^3J_{ax.-ax.}$ = 12.9 Hz, $^3J_{ax.-eq.}$ = 3.2 Hz, 2H).

Inhibitor 9

(1r,4S)-4-(aminomethyl)-N-((S)-3,8,11,15-tetraoxo-2,7,10,14-tetraaza-1,6(1,4)-dibenzenacyclohexadecaphane-4-yl)cyclohexane-1-carboxamide × TFA
or H-Txa-Phe(*c*[4-NH)-4-aPhac-βAla-Gly] × TFA

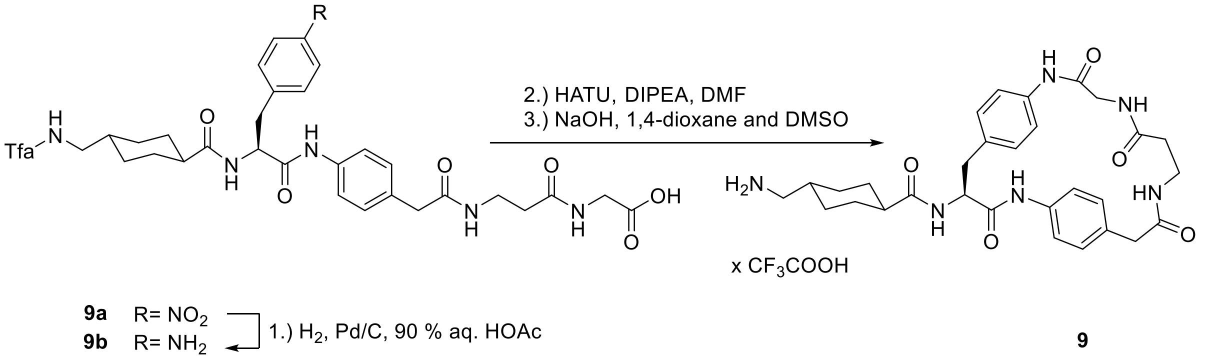

The synthesis was performed as described for inhibitor **12** in the main manuscript. The linear precursor was synthesized on 250 mg 2-CTC resin (0.4 mmol absolute) using Fmoc-Gly-OH, Fmoc-β-Ala-OH, Fmoc-4-aPhac-OH **(S10)**, Fmoc-Phe(4-NO_2)-OH, and Tfa-Txa-OH **(S1)**. After mild acidic cleavage from resin, the crude compound **9a** (HPLC method A: 20.85 min, MS calcd: 706.26, m/z found 705.42 $[M-H]^-$) was hydrogenated and the amine was purified by preparative HPLC yielding intermediate **9b** (102 mg colorless solid, 0.15 mmol, HPLC method A: 12.99 min, purity > 97 %, MS calcd.: 676.28, m/z found: 677.31 $[M+H]^+$). Compound **9b** was cyclized as described before. Afterwards, the solvent was removed and the remaining Tfa-protected derivative **9c** (structure not shown, HPLC method A: 16.90 min) was

treated with a mixture of 1 N aq. NaOH, 1,4-dioxane and DMSO as solvent for 1 h at rt to cleave the Tfa-protecting group. The final inhibitor **9** was purified by preparative HPLC (16 mg as colorless solid, 0.023 mmol, 16 % over two steps for cyclization and Tfa removal, HPLC method B: 19.93 min, start at 1 % B, purity > 98 %, MS calcd.: 562.29, m/z found: 563.47 $[M+H]^+$, ^{1}H NMR (500 MHz, DMSO-d_6): δ[ppm]= 9.54 (s, 1H), 8.89 (s, 1H), 8.17 (bs, 1H), 8.12 (d, ^3J = 7.4 Hz, 1H), 7.69 (bs, 3H), 7.37 (t, ^3J = 5.6 Hz, 1H), 7.27 (bs, 2H), 7.01 (d, ^3J = 7.4 Hz, 2H), 6.92 (d, ^3J = 8.1 Hz, 2H), 6.80 (bs, 1H), 4.59 – 4.40 (m, 1H), 3.93 – 3.80 (m, 1H), 3.71 – 3.59 (m, 1H), 3.34 – 3.17 (m, 4H), 3.03 (dd, ^2J = 12.5 Hz, ^2J = 6.0 Hz, 1H), 2.74 – 2.60 (m, 3H), 2.41 – 2.28 (m, 2H), 2.24 (tt, $^3J_{ax.-ax.}$ = 11.8 Hz, $^3J_{ax.-eq.}$ = 3.3 Hz, 1H), 1.85 – 1.71 (m, 4H), 1.60 – 1.46 (m, 1H), 1.42 – 1.29 (m, 2H), 0.97 (qd, $^3J_{ax.-ax.}$ = 13.9 Hz, $^3J_{ax.-eq.}$ = 3.7 Hz, 2H).

Inhibitor 10

(1r,4S)-4-(aminomethyl)-N-((4S,9S)-9-isobutyl-3,8,11,15-tetraoxo-2,7,10,14-tetraaza-1,6(1,4)-dibenzenacyclohexadecaphane-4-yl)cyclohexane-1-carboxamide × TFA
or H-Txa-Phe(c[4-NH)-4-aPhac-βAla-Leu] × TFA

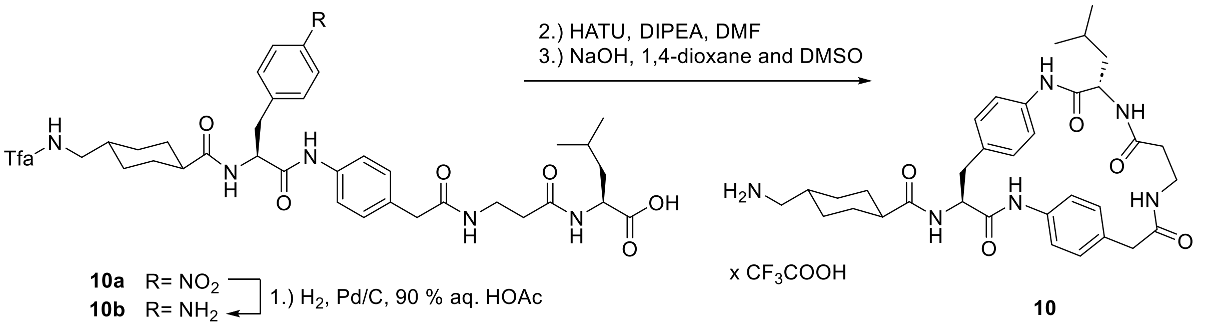

The synthesis was performed as described for inhibitor **12** in the main manuscript. The linear precursor was synthesized on 150 mg 2-CTC resin (0.24 mmol absolute) using Fmoc-Leu-OH, Fmoc-β-Ala-OH, Fmoc-4-aPhac-OH **(S10)**, Fmoc-Phe(4-NO_2)-OH, and Tfa-Txa-OH **(S1)**. After mild acidic cleavage from resin, the crude compound **10a** (HPLC method A: 24.23 min) was hydrogenated and the amine was purified by preparative HPLC yielding intermediate **10b** (93 mg colorless solid, 0.13 mmol, 3 %, HPLC method A: 16.66 min, purity > 99 %, MS calcd.: 732.35, m/z found: 733.27 $[M+H]^+$). Compound **10b** was cyclized as described before. Afterwards, the solvent was removed and the remaining crude Tfa-protected derivative **10c** (structure not shown, HPLC method A: 21.96 min, MS calcd.: 714.34, m/z found 715.37 $[M+H]^+$) was treated with a mixture of 1 N aq. NaOH, 1,4-dioxane and DMSO as solvent for 1

h at rt to cleave the Tfa-protecting group. The final inhibitor **10** was purified by preparative HPLC (22 mg colorless solid, 0.03 mmol, 24 % for cyclization and deprotection, HPLC method B: 23.05 min, start at 10 % B, purity > 98 %, MS calcd.: 618.35, m/z found: 619.34 $[M+H]^+$, ^{1}H NMR (500 MHz, DMSO-d_6): δ[ppm]= 9.78 (s, 0.3H), 9.66 (s, 0.7H), 8.95 (s, 0.7H), 8.89 (s, 0.3H), 8.13 (d, ^3J = 7.6 Hz, 0.3H), 8.08 (d, ^3J = 7.4 Hz, 0.7H), 8.01 (d, ^3J = 8.3 Hz, 0.7H), 7.98 (d, ^3J = 7.8 Hz, 0.3H), 7.78 – 7.55 (m, 4H), 7.40 (d, ^3J = 8.6 Hz, 0.5H), 7.27 (d, ^3J = 8.0 Hz, 1.5H), 7.06 (d, ^3J = 8.5 Hz, 1.5H), 7.00 (d, ^3J = 8.7 Hz, 0.5H), 6.93 (d, ^3J = 8.5 Hz, 0.5H), 6.84 (d, ^3J = 8.5 Hz, 1.5H), 6.81 – 6.69 (m, 2H), 4.59 (dt, ^2J = 11.0 Hz, ^3J = 7.0 Hz, 0.7H), 4.51 – 4.41 (m, 1H), 4.39 – 4.32 (m, 0.3H), 3.34 – 3.27 (m, 3H), 3.25 – 3.14 (m, 2H), 3.08 (dd, ^2J = 12.7 Hz, ^3J = 6.3 Hz, 0.7H), 3.02 (dd, ^2J = 12.7 Hz, ^3J = 6.2 Hz, 0.3H), 2.71 – 2.65 (m, 2H), 2.63 – 2.55 (m, 1H), 2.28 – 2.19 (m, 2H), 1.88 – 1.72 (m, 4H), 1.68 – 1.45 (m, 4H), 1.40 – 1.29 (m, 2H), 1.03 – 0.95 (m, 2H), 0.94 – 0.86 (m, 6H).

Inhibitor 11

(1r,4S)-4-(aminomethyl)-N-((4S,9R)-9-isobutyl-3,8,11,15-tetraoxo-2,7,10,14-tetraaza-1,6(1,4)-dibenzenacyclohexadecaphane-4-yl)cyclohexane-1-carboxamide × TFA
or H-Txa-Phe(c[4-NH])-4-aPhac-βAla-DLeu] × TFA

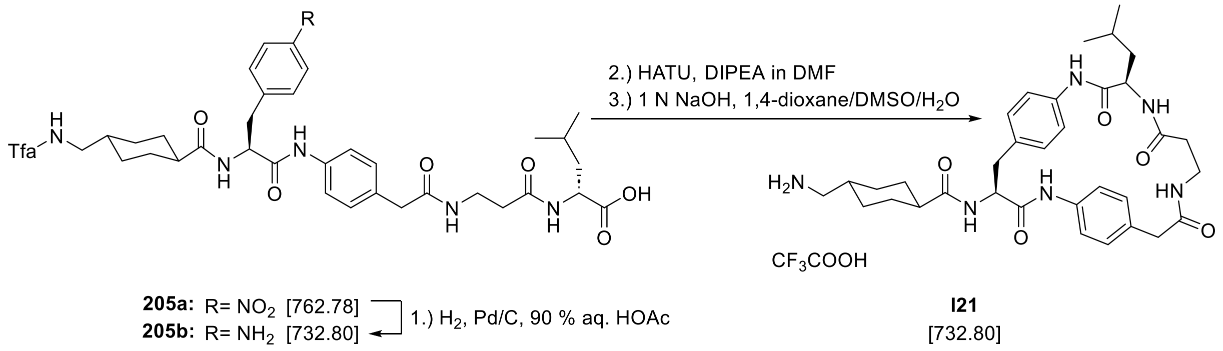

The synthesis was performed as described for inhibitor **12** in the main manuscript. The linear precursor was synthesized on 150 mg 2-CTC resin (0.24 mmol absolute) using Fmoc-DLeu-OH, Fmoc-β-Ala-OH, Fmoc-4-aPhac-OH **(S10)**, Fmoc-Phe(4-NO_2)-OH, and Tfa-Txa-OH **(S1)**. After mild acidic cleavage from resin, the crude compound **11a** (HPLC method A: 24.24 min, MS calcd.: 762.32, m/z found: 761.45 $[M-H]^-$) was hydrogenated and the amine was purified by preparative HPLC yielding intermediate **11b** (91 mg, 0.12 mmol colorless solid, 52 %, HPLC method A: 16.68 min, purity > 99 %, MS calcd.: 732.35, m/z found: 733.32 $[M+H]^+$).

Compound **11b** was cyclized as described before. Afterwards, the solvent was removed and the remaining crude Tfa-protected derivative **11c** (structure not shown, HPLC method A: 21.98 min, MS calcd.: 714.34, m/z found: 715.20 [M+H]+) was treated with a mixture of 1 N aq. NaOH, 1,4-dioxane and DMSO as solvent for 1 h at rt to cleave the Tfa-protecting group. The final inhibitor **11** was purified by preparative HPLC (27 mg, 0.037 mmol colorless lyophilized solid, 30.8 % over two steps, HPLC method A: 14.46 min, purity > 99 %, MS : ber.: 618.35, m/z: 619.50 $[M+H]^+$, **^{1}H NMR (500 MHz, DMSO-d6):** δ[ppm] = 9.78 (s, 0.4H), 9.66 (s, 0.6H), 8.96 (s, 0.6H), 8.89 (s, 0.4H), 8.13 (d, J = 7.5 Hz, 0.4H), 8.08 (d, J = 7.4 Hz, 0.6H), 8.02 (d, J = 8.3 Hz, 0.6H), 7.98 (d, J = 7.8 Hz, 0.4H), 7.78 - 7.65 (m, 4H), 7.40 (d, 3J = 8.7 Hz, 0.6H), 7.27 (d, 3J = 7.8 Hz, 1.4H), 7.06 (d, 3J = 8.5 Hz, 1.2H), 7.00 (d, 3J = 8.6 Hz, 0.8H), 6.93 (d, 3J = 8.5 Hz, 0.8H), 6.84 (d, 3J = 8.5 Hz, 1.2H), 6.78 (d, 3J = 8.5 Hz, 0.8H), 6.76 (d, 3J = 8.5 Hz, 1.2H), 4.59 (dt, 2J = 11.1 Hz, 3J = 7.1 Hz, 0.6H), 4.51 - 4.40 (m, 1H), 4.40 - 4.32 (m, 0.4H), 3.41 - 3.27 (m, 3H), 3.25 - 3.12 (m, 2H), 3.08 (dd, 2J = 12.5 Hz, 3J = 6.5 Hz, 0.6H), 3.02 (dd, 2J = 12.5 Hz, 3J = 6.1 Hz, 0.4H), 2.72 - 2.64 (m, 2H), 2.64 - 2.57 (m, 1H), 2.28 - 2.18 (m, 2H), 1.84 - 1.74 (m, 4H), 1.66 - 1.43 (m, 4H), 1.40 - 1.31 (m, 2H), 1.03 - 0.94 (m, 2H), 0.94 - 0.86 (m, 6H).

Inhibitor 12

The synthesis of inhibitor **12** is described in the main manuscript.

7. Synthesis of inhibitors of second series (Table 2 in main manuscript)

Inhibitor 22

(1r,4S)-4-(Aminomethyl)-N-((S)-14-methyl-5,9,16-trioxo-6,10,17-triaza-1(1,3),2,7(1,4)-tribenzenacycloheptadecaphane-4-yl)cyclohexane-1-carboxamide x TFA
or H-Txa-Bpa(4'-Me, c[3'-NH)-4-aPhac-Aca] × TFA

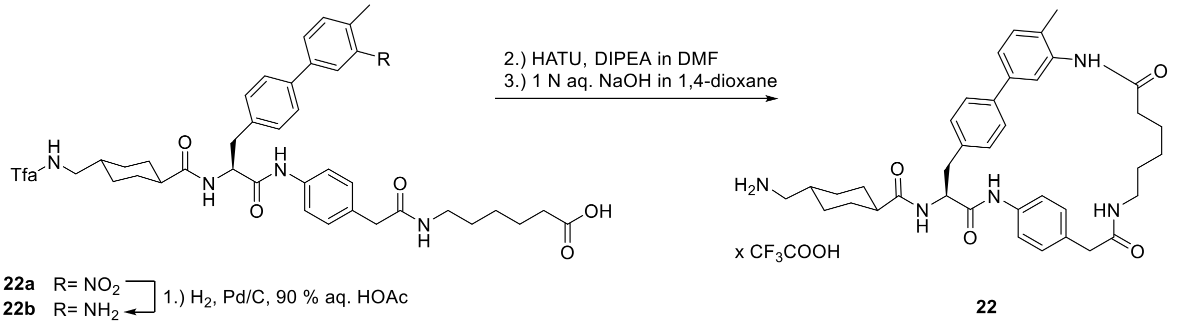

The linear precursor **22a** (HPLC method A: 28.53 min, MS calcd. 781.33, m/z found: 780.7 $[M-H]^-$) was synthesized on 150 mg 2-CTC resin (0.24 mmol absolute) as described for inhibitor **33** in the main manuscript using commercially available Fmoc-6-Aca-OH, and building blocks Fmoc-4-aPha-OH (**S10)**, Fmoc-Bpa(4'-Me,3'-NO_2)-OH (**S4)**, and Tfa-Txa-OH (**S1)**. Hydrogenation of the crude peptide **22a** provided the amine **22b**, which was purified by preparative HPLC (29 mg colorless solid, 0.038 mmol, HPLC method A: 19.35 min, purity > 99 %, MS calcd.: 751.36, m/z found: 752.32 $[M+H]^+$, ^{1}H NMR (500 MHz, DMSO-d_6): δ[ppm]= 10.02 (s, 1H), 9.34 (t, ^3J = 5.8 Hz, 1H), 8.05 (d, ^3J = 8.4 Hz, 1H), 7.93 (t, ^3J = 5.6 Hz, 1H), 7.52 – 7.47 (m, 4H), 7.35 (d, ^3J = 8.2 Hz, 2H), 7.28 (s, 1H), 7.23 – 7.15 (m, 4H), 4.67 (td, ^3J = 8.7 Hz, 5.1 Hz, 1H), 3.09 – 2.95 (m, 5H), 2.89 (dd, ^2J = 13.7 Hz, ^3J = 9.5 Hz, 1H), 2.22 (s, 3H), 2.18 (t, ^3J = 7.4 Hz, 2H), 2.15 – 2.08 (m, 1H), 1.78 – 1.56 (m, 4H), 1.54 – 1.33 (m, 5H), 1.33 – 1.10 (m, 6H), 0.99 – 0.67 (m, 2H). 13C NMR (126 MHz, DMSO-D6): δ[ppm] = 175.00, 174.34, 170.12, 169.92, 156.45, 156.16, 138.57, 137.64, 137.08, 137.02, 131.54, 131.18, 129.77, 129.11, 125.84, 119.27, 116.87, 115.32, 114.85, 54.36, 45.07, 43.45, 41.80, 38.43, 36.33, 33.57, 29.36, 29.25, 28.76, 28.50, 28.31, 25.91, 24.15, 16.69.

Compound **22b** was cyclized as described for inhibitor **33** in the main manuscript. The crude Tfa-protected cyclization product (**22c**, HPLC method A: 24.96 min, MS calcd.: 733.35, m/z found 734.60 $[M+H]^+$) was deprotected as described for inhibitor **33**, followed by preparative HPLC (9 mg colorless solid, 0.012 mmol, 31 % over two steps for cyclization and Tfa removal). HPLC method B:, 27.81 min, start at 10 % B, purity > 98 %, MS calcd.: 637.36, m/z found:

638.45 [M+H]$^+$, ^{1}H NMR (500 MHz, DMSO-d_6): δ[ppm]= 9.35 (s, 1H), 9.17 (s, 1H), 8.11 (d, ^3J = 7.5 Hz, 1H), 7.90 – 7.83 (m, 1H), 7.68 (s, 3H), 7.64 – 7.57 (m, 1H), 7.44 – 7.32 (m, 2H), 7.31 – 7.13 (m, 4H), 7.11 – 6.96 (m, 4H), 4.63 – 4.52 (m, 1H), 3.33 – 3.23 (m, 2H), 3.09 – 2.97 (m, 3H), 2.91 – 2.78 (m, 1H), 2.72 – 2.66 (m, 2H), 2.45 – 2.35 (m, 2H), 2.31 – 2.18 (m, 4H), 1.88 – 1.77 (m, 4H), 1.65 – 1.58 (m, 2H), 1.56 – 1.46 (m, 1H), 1.46 – 1.27 (m, 6H), 1.05 – 0.86 (m, 2H).

Inhibitor 23

(1r,4S)-4-(aminomethyl)-N-((51S,54S,13S)-24-methyl-4,8,12-trioxo-3,7,11-triaza-1,10(1,4),2(1,3)-tribenzena-5(1,4)-cyclohexanacyclotetradecaphane-13-yl)cyclohexane-1-carboxamide × TFA
or H-Txa-Bpa(4'-Me,*c*[3'-NH)-4-aPhac-Txa] × TFA

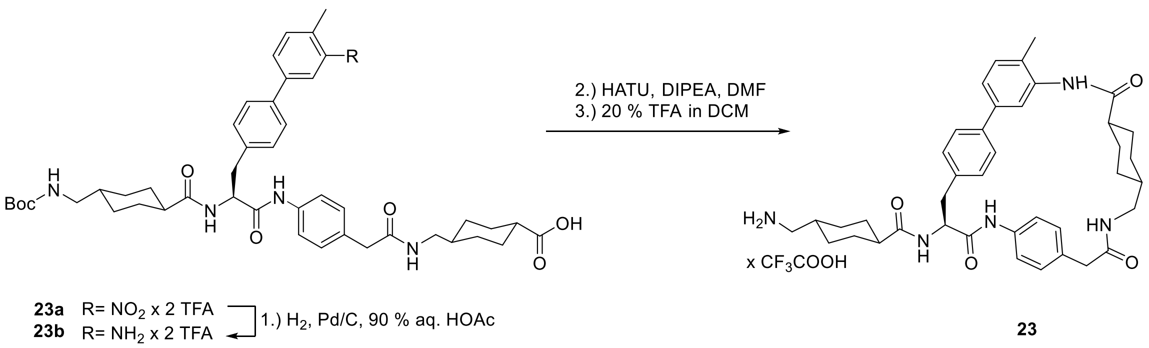

The synthesis was performed as described for inhibitor **33** in the main manuscript. The linear precursor was synthesized on 130 mg 2-CTC resin (0.208 mmol absolute) using building blocks Fmoc-Txa-OH **(S8)**, Fmoc-4-aPhac-OH (**S10)**, Fmoc-Bpa(4'-Me,3'-NO_2)-OH (**S4)**, and Boc-Txa-OH **(S2)**. After mild acidic cleavage from resin, the crude compound **23a** (HPLC method A: 31.24 min) was hydrogenated and the amine was purified by preparative HPLC yielding intermediate **23b** (75 mg, 0.1 mmol colorless solid, 46 %, HPLC method A: 22.26 min, purity > 99 %). Compound **23b** was cyclized as described before. Afterwards, the solvent was removed and the remaining crude Boc-protected derivative **23c** (structure not shown, HPLC method A: 27.14 min) was treated with 4 mL 20 % TFA in DCM (v/v) at room temperature. The final inhibitor **23** was purified by preparative HPLC (28 mg, 0.036 mmol colorless lyophilized solid, 36 % over two steps, HPLC method A: 17.50 min, purity > 98 %, MS calcd.: 663.83, m/z found: 664.54 [M+H]$^+$).

Inhibitor 24

(1r,4S)-4-(aminomethyl)-N-((S)-24-methyl-4,8,12-trioxo-3,7,11-triaza-1,6,10(1,4),2(1,3)-tetrabenzenacyclotetradecaphane-13-yl)cyclohexane-1-carboxamide × TFA
or H-Txa-Bpa(4'-Me, c[3'-NH)-4-aPhac-4-aPhac] × TFA

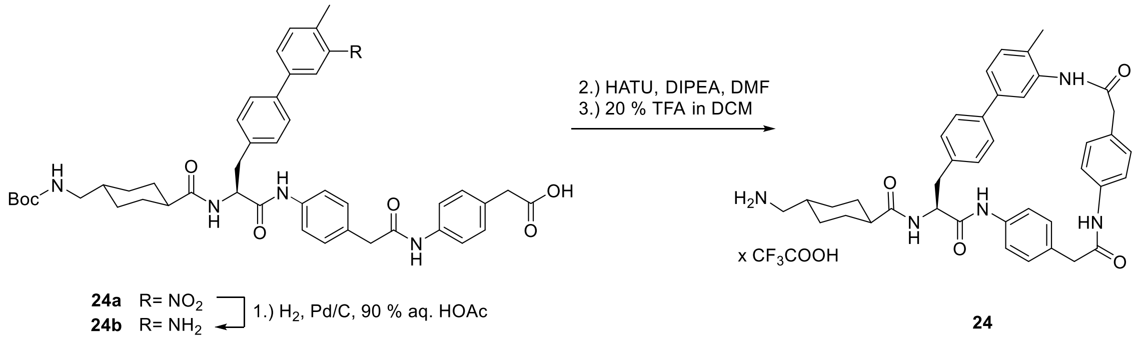

The synthesis was performed as described for inhibitor **33** in the main manuscript. The linear precursor was synthesized on 130 mg 2-CTC resin (0.208 mmol absolute) using the following building blocks 2 × Fmoc-4-aPhac-OH **(S10)**, Fmoc-Bpa(4'-Me,3'-NO_2)-OH **(S4)**, and Boc-Txa-OH **(S2)**. After mild acidic cleavage from resin, the crude compound **24a** (HPLC method A: 31.73 min) was hydrogenated and the amine was purified by preparative HPLC yielding intermediate **24b** (94 mg, 0.12 mmol colorless solid, 58 %, HPLC method A: 22.62 min, purity > 99 %).

Compound **24b** was cyclized as described before. Afterwards, the solvent was removed and the remaining crude Boc-protected derivative **24c** (structure not shown, HPLC method A: 28.85) was stirred 1 h with 4 mL 20 % TFA in DCM at room temperature. The final inhibitor **24** was purified by preparative HPLC (19 mg, 0.025 mmol colorless lyophilized solid, 21 % over two steps, HPLC method A: 18.93 min, purity > 99 %, MS calcd..: 657.33, m/z found: 658.40 $[M+H]^+$).

Inhibitor 25

(1r,4S)-4-(Aminomethyl)-N-((S)-14-methyl-5,9,13,16-tetraoxo-6,10,14,17-tetraaza-1(1,3),2,7(1,4)-tribenzenacycloheptadecaphane-4-yl)cyclohexane-1-carboxamide × TFA or H-Txa-Bpa(4'-Me,*c*[3'-NH)-4-aPhac-βAla-Gly] × TFA

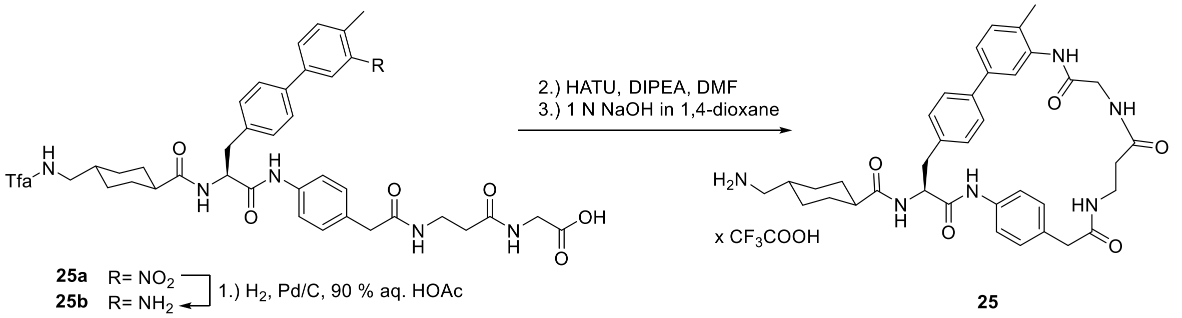

The synthesis was performed as described for inhibitor **33** in the main manuscript. The linear precursor was synthesized on 150 mg 2-CTC resin (0.24 mmol absolute) using Fmoc-Gly-OH, Fmoc-β-Ala-OH, Fmoc-4-aPhac-OH **(S10)**, Fmoc-Bpa(4'-Me,3'-NO_2)-OH **(S4)**, and Tfa-Txa-OH **(S1)**. After mild acidic cleavage from resin, the crude compound **25a** (HPLC method A: 27.45 min) was hydrogenated and the amine was purified by preparative HPLC yielding intermediate **25b** (55 mg, 0.07 mmol colorless solid, 30 %, HPLC method A: 18.34 min, purity > 99 %, MS calcd.: 766.33, m/z found: 767.23 $[M+H]^+$). **^{1}H NMR (500 MHz, DMSO-*d*$_6$):** δ[ppm]= 10.02 (s, 1H), 9.34 (t, ^3J = 5.6 Hz, 1H), 8.19 (t, ^3J = 5.8 Hz, 1H), 8.05 (d, ^3J = 8.3 Hz, 1H), 7.96 (t, ^3J = 5.6 Hz, 1H), 7.55 – 7.44 (m, 4H), 7.35 (d, ^3J = 8.1 Hz, 2H), 7.25 (s, 1H), 7.24 – 7.12 (m, 4H), 4.67 (td, ^3J = 8.6 Hz, 5.2 Hz, 1H), 3.74 (d, ^3J = 5.9 Hz, 2H), 3.34 (s, 2H), 3.24 (q, ^3J = 6.9 Hz, 2H), 3.11 – 2.98 (m, 3H), 2.89 (dd, ^2J = 13.7Hz, ^3J = 9.6 Hz, 1H), 2.30 (t, ^3J = 7.1 Hz, 2H), 2.21 (s, 3H), 2.14 (tt, $^3J_{ax.-ax.}$ = 11.9 Hz, $^3J_{ax.-eq.}$ = 2.7 Hz, 1H), 1.75 – 1.56 (m, 4H), 1.49 – 1.37 (m, 1H), 1.25 (qd, J = $^3J_{ax.-ax.}$ = 13.2 Hz, $^3J_{ax.-eq.}$ = 3.1 Hz, 1H), 1.21 – 1.14 (m, 1H), 0.98 – 0.78 (m, 2H). 13C NMR (126 MHz, DMSO-D6): δ[ppm] = 175.59, 171.86, 171.30, 170.72, 170.70, 157.03, 139.13, 138.28, 137.67, 137.55, 131.96, 131.73, 130.34, 129.75, 126.41, 120.00, 119.85, 54.95, 45.65, 44.03, 42.28, 41.09, 37.96, 36.91, 35.86, 35.58, 29.93, 29.83, 29.08, 28.89, 17.28.

Compound **25b** was cyclized as described before. Afterwards, the solvent was removed and the remaining crude Tfa-protected derivative **25c** (structure not shown, HPLC method A: 23.39 min) was treated with a mixture of 1 N aq. NaOH, 1,4-dioxane and DMSO as solvent for 1 h at rt to cleave the Tfa-protecting group. The final inhibitor **11** was purified by preparative HPLC (14 mg, 0.018 mmol colorless lyophilized solid, 25.7 % over two steps, HPLC method B:

24.29 min start at 10 % B, purity > 99 %, MS calcd.: 652.34, m/z found: 653.50 $[M+H]^+$, **^{1}H NMR (500 MHz, DMSO-d6):** δ[ppm] = 9.33 (s, 1H), 9.16 (s, 1H), 8.20 (t, ^3J = 6.0 Hz, 1H), 8.13 (d, ^3J = 7.5 Hz, 1H), 7.95 – 7.81 (m, 1H), 7.74 (bs, 4H), 7.35 (d, ^3J = 7.7 Hz, 2H), 7.27 (s, 2H), 7.17 (d, ^3J = 8.1 Hz, 2H), 7.09 (d, ^3J = 8.4 Hz, 2H), 6.84 (d, ^3J = 8.1 Hz, 2H), 4.79 – 4.45 (m, 1H), 4.10 (dd, ^2J = 15.9 Hz, ^3J = 4.8 Hz, 1H), 3.91 (dd, ^2J = 16.4 Hz, ^3J = 5.1 Hz, 1H), 3.38 – 3.33 (m, 2H), 3.32 – 3.27 (m, 1H), 3.05 (dd, ^2J = 12.6 Hz, ^3J = 5.0 Hz, 1H), 2.88 – 2.72 (m, 1H), 2.68 (t, ^3J = 6.2 Hz, 2H), 2.43 – 2.21 (m, 7H), 1.91 – 1.74 (m, 4H), 1.53 (ttt, $^3J_{ax.-ax.}$ = 10.3 Hz, ^3J = 6.6 Hz, $^3J_{ax.-eq.}$ = 3.4 Hz, 1H), 1.44 – 1.31 (m, 2H), 1.06 – 0.86 (m, 2H). **13C NMR (126 MHz, DMSO-D6):** δ[ppm] = 174.63, 171.48, 170.17, 169.32, 138.53, 136.54, 135.79, 135.53, 131.80, 130.70, 129.85, 128.89, 128.66, 126.55, 122.65, 121.18, 54.94, 44.36, 43.03, 42.20, 40.02, 38.65, 35.51, 35.18, 35.07, 28.90, 28.87, 28.36, 28.31, 17.37.

Inhibitor 26

(1r,4S)-4-(aminomethyl)-N-((S)-14-methyl-5,9,12,16-tetraoxo-6,10,13,17-tetraaza-1(1,3),2,7(1,4)-tribenzenacycloheptadecaphane-4-yl)cyclohexane-1-carboxamide × TFA or H-Txa-Bpa(4'-Me, c[3'-NH)-4-aPhac-Gly-βAla] × TFA

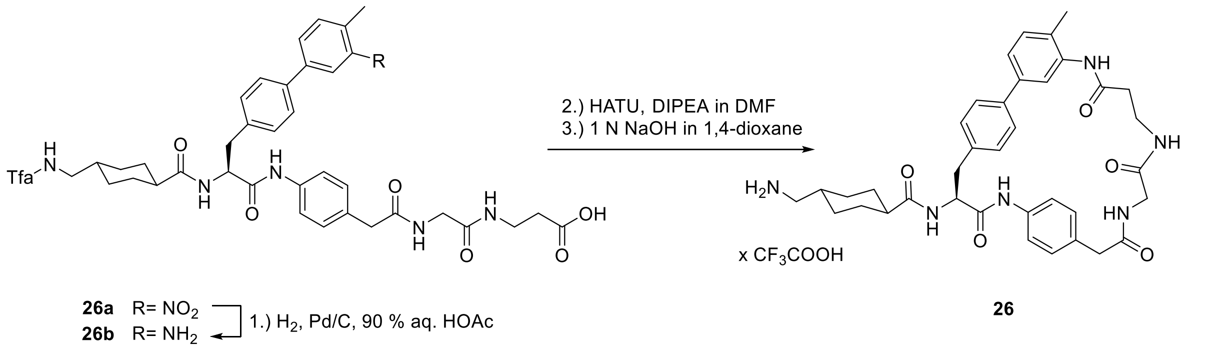

The synthesis was performed as described for inhibitor **33** in the main manuscript. The linear precursor was synthesized on 150 mg 2-CTC resin (0.24 mmol absolute) using Fmoc-β-Ala-OH, Fmoc-Gly-OH, Fmoc-4-aPhac-OH **(S10)**, Fmoc-Bpa(4'-Me,3'-NO_2)-OH **(S4)**, and Tfa-Txa-OH **(S1)**. After mild acidic cleavage from resin, the crude compound **26a** (HPLC method A: 26.04 min, MS calcd.: 762.32, m/z found: 761.45 $[M-H]^-$) was hydrogenated and the amine was purified by preparative HPLC yielding intermediate **26b** (35 mg, 0.05 mmol colorless solid, 19 %, HPLC method A: 17.29 min, purity > 99 %, MS calcd.: 766.33, m/z found: 767.39 $[M+H]^+$). ^{1}H NMR (500 MHz, DMSO-d_6): δ[ppm]= 10.03 (s, 1H), 9.34 (t, ^3J = 5.9 Hz, 1H),

8.15 (t, ^3J = 5.9 Hz, 1H), 8.05 (d, ^3J = 8.4 Hz, 1H), 7.90 (t, ^3J = 5.6 Hz, 1H), 7.59 – 7.47 (m, 3H), 7.42– 7.33 (m, 3H), 7.28 (s, 1H), 7.24 – 7.18 (m, 4H), 4.71 – 4.62 (m, 1H), 3.65 (d, ^3J = 5.8 Hz, 2H), 3.43 (s, 2H), 3.32 – 3.20 (m, 2H), 3.13 – 2.97 (m, 3H), 2.90 (dd, ^2J = 13.7 Hz, ^3J = 9.5 Hz, 1H), 2.38 (t, ^3J = 6.9 Hz, 2H), 2.22 (s, 3H), 2.14 (tt, $^3J_{ax.-ax.}$ = 12.1 Hz, $^3J_{ax.-eq.}$ = 3.4 Hz, 1H), 1.75 – 1.53 (m, 4H), 1.49 – 1.33 (m, 1H), 1.33 – 1.08 (m, 2H), 0.97 – 0.74 (m, 2H). ^{13}C NMR (126 MHz, DMSO-d_6): δ[ppm]= 175.00, 172.75, 170.46, 170.12, 168.76, 158.28, 158.01, 157.73, 156.45, 156.16, 138.58, 137.61, 137.15, 137.03, 131.20, 129.78, 129.30, 125.84, 119.25, 54.36, 45.08, 43.46, 42.00, 41.47, 37.38, 36.34, 34.71, 33.79, 29.36, 29.25, 28.51, 28.32, 16.68.

Compound **26b** was cyclized as described before. Afterwards, the solvent was removed and the remaining crude Tfa-protected derivative **26c** (structure not shown, HPLC method A: 22.30 min, MS calcd.: 714.34, m/z found: 715.20 [M+H]+) was treated with a mixture of 1 N aq. NaOH, 1,4-dioxane and DMSO as solvent for 1 h at rt to cleave the Tfa-protecting group. The final inhibitor **26** was purified by preparative HPLC (21 mg, 0.027 mmol colorless lyophilized solid, 54 % over two steps, HPLC method A: 16.12 min, purity > 99 %, MS calcd.: 652.34, m/z found: 653.52 $[M+H]^+$, ^{1}H NMR (500 MHz, DMSO-d6): δ[ppm] = 9.28 (pd, 2H), 8.13 (d, ^3J = 7.5 Hz, 1H), 8.10 – 8.00 (m, 1H), 7.79 – 7.59 (m, 5H), 7.37 (d, ^3J = 7.4 Hz, 2H), 7.31 – 7.23 (m, 2H), 7.20 (d, ^3J = 8.2 Hz, 2H), 7.11 (d, ^3J = 8.5 Hz, 2H), 6.97 (d, ^3J = 8.2 Hz, 2H), 4.67 – 4.51 (m, 1H), 3.74 – 3.62 (m, 2H), 3.44 – 3.36 (m, 4H), 3.05 (dd, ^2J = 13.1 Hz, ^3J = 5.4 Hz, 1H), 2.87 – 2.77 (m, 1H), 2.73 – 2.65 (m, 2H), 2.57 (bs, 2H), 2.30 – 2.19 (m, 4H), 1.89 – 1.77 (m, 4H), 1.59 – 1.46 (m, 1H), 1.37 1.45 – 1.30 (m, 2H), 1.03 – 0.92 (m, 2H).

Inhibitor 27

(1r,4S)-4-(Aminomethyl)-N-((S)-14-methyl-5,9,12,17-tetraoxo-6,10,13,18-tetraaza-1(1,3),2,7(1,4)-tribenzenacyclooctadecaphane-4-yl)cyclohexane-1-carboxamide × TFA
or H-Txa-Bpa(4'-Me,*c*[3'-NH)-4-aPhac-Gly-Gaba] × TFA

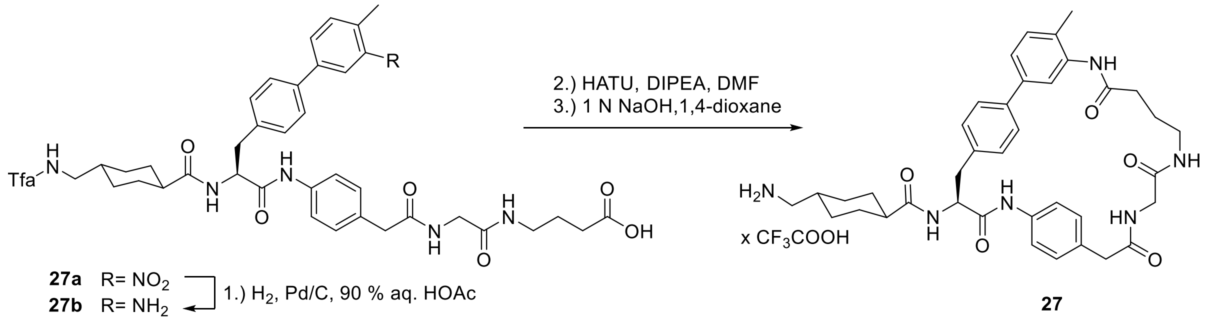

The synthesis was performed as described for inhibitor **33** in the main manuscript. The linear precursor was synthesized on 150 mg 2-CTC resin (0.24 mmol absolute) using Fmoc-Gaba-OH, Fmoc-Gly-OH, Fmoc-4-aPhac-OH **(S10)**, Fmoc-Bpa(4'-Me,3'-NO_2)-OH **(S4)**, and Tfa-Txa-OH **(S1)**. After mild acidic cleavage from resin, the crude compound **27a** (HPLC method A: 27.90 min, MS calcd.: 810.32, m/z found: 809.58 $[M-H]^-$) was hydrogenated and the amine was purified by preparative HPLC yielding intermediate **27b** (70 mg, 0.09 mmol colorless solid, 38 %, HPLC method A: 18.81 min, purity > 97 %, MS calcd.: 780.35, m/z found: 781.31 $[M+H]^+$). ^1H NMR (500 MHz, DMSO-d_6): δ[ppm]= 10.02 (s, 1H), 9.34 (t, ^3J = 5.8 Hz, 1H), 8.14 (t, ^3J = 5.8 Hz, 1H), 8.05 (d, ^3J = 8.4 Hz, 1H), 7.84 (t, ^3J = 5.7 Hz, 1H), 7.57 – 7.43 (m, 4H), 7.34 (d, ^3J = 8.2 Hz, 2H), 7.29 – 7.08 (m, 5H), 4.67 (td, ^3J = 8.7 Hz, 5.2 Hz, 1H), 3.65 (d, ^3J = 5.7 Hz, 2H), 3.43 (s, 2H), 3.17 – 2.97 (m, 5H), 2.94 – 2.79 (m, 1H), 2.26 – 2.09 (m, 6H), 1.77 – 1.54 (m, 6H), 1.50 – 1.37 (m, 1H), 1.31 – 1.13 (m, 2H), 0.94 – 0.81 (m, 2H). ^{13}C NMR (126 MHz, DMSO-d6): δ[ppm] = 175.01, 174.12, 170.47, 170.12, 168.66, 138.54, 137.77, 137.14, 136.94, 131.24, 131.21, 131.11, 129.76, 129.30, 125.83, 119.25, 54.37, 45.07, 43.46, 42.09, 41.51, 37.88, 37.37, 36.34, 30.97, 29.36, 29.25, 28.51, 28.31, 24.51, 16.72.

Compound **27b** was cyclized as described before. Afterwards, the solvent was removed and the remaining crude Tfa-protected derivative **27c** (structure not shown, HPLC method A: 23.96 min) was treated with a mixture of 1 N aq. NaOH, 1,4-dioxane, THF and acetone as solvent for 1 h at rt to cleave the Tfa-protecting group. The final inhibitor **27** was purified by preparative HPLC (44 mg, 0.058 mmol colorless lyophilized solid, 64 % over two steps, HPLC method B: 23.26 min start at 10 % solvent B, purity > 99 %, MS calcd.: 666.35, m/z found: 667.47

[M+H]+, ^{1}H NMR (500 MHz, DMSO-d_6): δ[ppm]= 9.74 (bs, 1H), 9.39 (s, 1H), 8.06 (d, ^{3}J = 7.6 Hz, 1H), 7.99 (t, ^{3}J = 5.4 Hz, 1H), 7.90 (t, ^{3}J = 5.6 Hz, 1H), 7.76 (s, 3H), 7.43 (d, ^{3}J = 8.1 Hz, 2H), 7.39 – 7.30 (m, 4H), 7.26 (d, ^{3}J = 8.1 Hz, 1H), 7.25 – 7.16 (m, 4H), 4.77 – 4.45 (m, 1H), 3.67 (d, ^{3}J = 5.8 Hz, 1H), 3.65 – 3.56 (m, 1H), 3.43 – 3.31 (m, 2H), 3.18 – 3.05 (m, 2H), 3.04 – 2.96 (m, 2H), 2.73 – 2.62 (m, 2H), 2.38 – 2.23 (m, 3H), 2.20 (s, 3H), 1.92 – 1.76 (m, 4H), 1.75 – 1.65 (m, 2H), 1.61 – 1.47 (m, 1H), 1.46 – 1.30 (m, 2H), 1.03 – 0.87 (m, 2H). ^{13}C NMR (126 MHz, DMSO-d_6): δ[ppm]= 174.57, 170.90, 170.44, 169.10, 168.47, 138.08, 137.80, 136.74, 135.76, 131.28, 131.17, 130.72, 129.83, 129.30, 129.29, 126.23, 124.22, 123.17, 119.67, 54.81, 44.36, 42.99, 41.96, 41.66, 38.30, 37.82, 35.08, 32.73, 28.91, 28.84, 28.41, 28.26, 25.62, 17.41.

Inhibitor 28

(1r,4S)-4-(Aminomethyl)-N-((S)-14-methyl-5,9,14,17-tetraoxo-6,10,15,18-tetraaza-1(1,3),2,7(1,4)-tribenzenacyclooctadecaphane-4-yl)cyclohexane-1-carboxamide × TFA
or H-Txa-Bpa(4'-Me,*c*[3'-NH)-4-aPhac-Gaba-Gly] × TFA

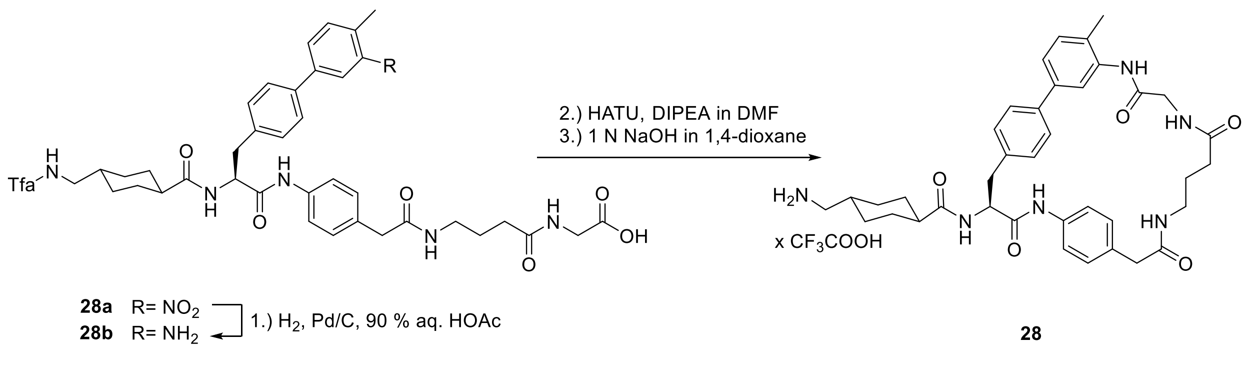

The synthesis was performed as described for inhibitor **33** in the main manuscript. The linear precursor was synthesized on 150 mg 2-CTC resin (0.24 mmol absolute) using Fmoc-Gly-OH, Fmoc-Gaba-OH, Fmoc-4-aPha-OH **(S10)**, Fmoc-Bpa(4'-Me,3'-NO_2)-OH **(S4)**, and Tfa-Txa-OH **(S1)**. After mild acidic cleavage from resin, the crude compound **28a** (HPLC method A: 25.92 min) was hydrogenated and the amine was purified by preparative HPLC yielding intermediate **28b** (112 mg, 0.14 mmol colorless solid, 60 %, HPLC method A: 16.88 min, purity > 93 %, MS calcd.: 780.35, m/z found: 781.45 [M+H]+, ^{1}H NMR (500 MHz, DMSO-d_6): δ[ppm]= 9.98 (t, ^{3}J = 7.3 Hz, 1H), 9.31 (s, 1H), 8.08 (t, ^{3}J = 5.9 Hz, 1H), 8.01 (d, ^{3}J = 8.3 Hz, 1H), 7.93 (t, ^{3}J = 5.6 Hz, 1H), 7.49 – 7.44 (m, 4H), 7.31 (d, ^{3}J = 8.3 Hz, 2H), 7.18 (bs, 1H),

7.16 – 7.12 (m, 3H), 7.15 – 7.06 (m, 1H), 4.64 (td, ^3J = 8.6 Hz, 5.1 Hz, 1H), 3.69 (d, ^3J = 5.9 Hz, 2H), 3.09 – 3.05 (m, 4H), 3.04 – 2.94 (m, 1H), 2.86 (dd, ^2J = 13.7 Hz, ^3J = 9.5 Hz, 1H), 2.17 (s, 3H), 2.13 – 2.05 (m, 3H), 1.75 – 1.47 (m, 6H), 1.46 – 1.34 (m, 1H), 1.28 – 1.17 (m, 2H), 0.92 – 0.78 (m, 2H). Compound **28b** was cyclized as described before. Afterwards, the solvent was removed and the remaining crude Tfa-protected derivative **28c** (structure not shown, HPLC method A: 22.97 min) was treated with a mixture of 1 N aq. NaOH, 1,4-dioxane, THF, and acetone as solvent for 1 h at rt to cleave the Tfa-protecting group. The final inhibitor **28** was purified by preparative HPLC (35 mg, 0.045 mmol colorless lyophilized solid, 32 % over two steps, HPLC method B: 22.02 min start at 10 % solvent B, purity > 99 %, MS calcd.: 666.35, m/z found: 667.38 $[M+H]^+$).

Inhibitor 29

(1r,4S)-4-(Aminomethyl)-N-((S)-14-methyl-5,9,15,17-tetraoxo-6,10,16,19-tetraaza-1(1,3),2,7(1,4)-tribenzenacyclononadecaphane-4-yl)cyclohexane-1-carboxamide × TFA
or H-Txa-Bpa(4'-Me,*c*[3'-NH)-4-aPhac-5-Ava-Gly] × TFA

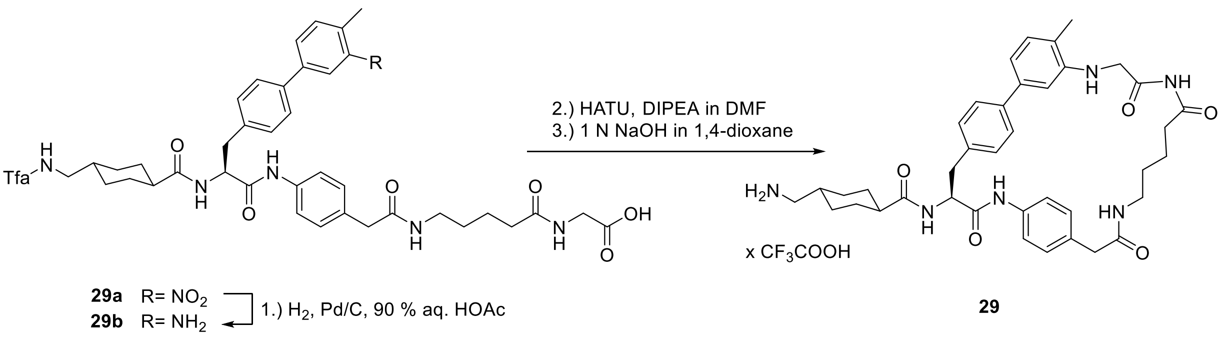

The synthesis was performed as described for inhibitor **33** in the main manuscript. The linear precursor was synthesized on 150 mg 2-CTC resin (0.24 mmol absolute) using Fmoc-Gly-OH, Fmoc-5-Ava-OH, Fmoc-4-aPha-OH **(S10)**, Fmoc-Bpa(4'-Me,3'-NO_2)-OH **(S4)**, and Tfa-Txa-OH **(S1)**. After mild acidic cleavage from resin, the crude compound **29a** (HPLC method A: 27.90 min, MS calcd.: 824.34, m/z found: 823.54 $[M-H]^-$) was hydrogenated and the amine was purified by preparative HPLC yielding intermediate **29b** (76 mg, 0.10 mmol colorless solid, 41 %, HPLC method A: 18.93 min, purity > 97 %, MS calcd.: 794.36, m/z found: 795.28 $[M+H]^+$, ^{1}H NMR (500 MHz, DMSO-d_6): δ[ppm]= 10.02 (s, 1H), 9.34 (t, ^3J = 5.7 Hz, 1H), 8.17 – 8.01 (m, 2H), 7.94 (t, ^3J = 5.5 Hz, 1H), 7.56 – 7.46 (m, 4H), 7.35 (d, ^3J = 8.3 Hz, 2H), 7.24 (s, 1H), 7.22 – 7.11 (m, 4H), 4.67 (td, ^3J = 8.8 Hz, 5.1 Hz, 1H), 3.72 (d, ^3J = 5.9 Hz, 2H), 3.33 (s, 2H), 3.10 – 2.95 (m, 5H), 2.89 (dd, ^2J = 13.8 Hz, ^3J = 9.5 Hz, 1H), 2.21 (s, 3H), 2.14 – 2.04 (m, 3H),

1.77 – 1.55 (m, 4H), 1.56 – 1.33 (m, 5H), 1.31 – 1.12 (m, 2H), 0.96 – 0.77 (m, 2H), ^{13}C NMR (126 MHz, DMSO-d_6): δ[ppm]= 175.01, 172.36, 170.12, 169.94, 156.31 (d, $^{2}J_{C\text{-}F}$ = 35.7 Hz), 150.94, 138.55, 137.74, 137.07, 136.96, 131.54, 131.13, 129.76, 129.13, 125.84, 119.28, 115.99 (d, $^{2}J_{f}$ = 288.4 Hz), 54.36, 45.07, 43.46, 41.78, 40.50, 38.36, 37.38, 36.34, 34.64, 29.36, 29.25, 28.64, 28.51, 28.32, 22.61, 16.71.

Compound **29b** was cyclized as described before. Afterwards, the solvent was removed and the remaining crude Tfa-protected derivative **29c** (structure not shown, HPLC method A: 23.83 min, MS calcd.: 714.34, m/z found: 715.20 [M+H]+) was treated with a mixture of 1 N aq. NaOH, 1,4-dioxane and DMSO as solvent for 1 h at rt to cleave the Tfa-protecting group. The final inhibitor **11** was purified by preparative HPLC (9 mg, 0.011 mmol colorless lyophilized solid, 12 % over two steps, HPLC method A: 16.07 min, purity > 98 %, MS calcd.: 680.37, m/z found: 681.43 $[M+H]^+$).

Inhibitor 30

(1r,4S)-4-(Aminomethyl)-N-((4S,16S)-16-benzyl-14-methyl-5,9,14,17-tetraoxo-6,10,15,18-tetraaza-1(1,3),2,7(1,4)-tribenzenacyclooctadecaphane-4-yl)cyclohexane-1-carboxamide × TFA or H-Txa-Bpa(4’-Me, c[3’-NH)-4-aPhac-Gaba-Phe] × TFA

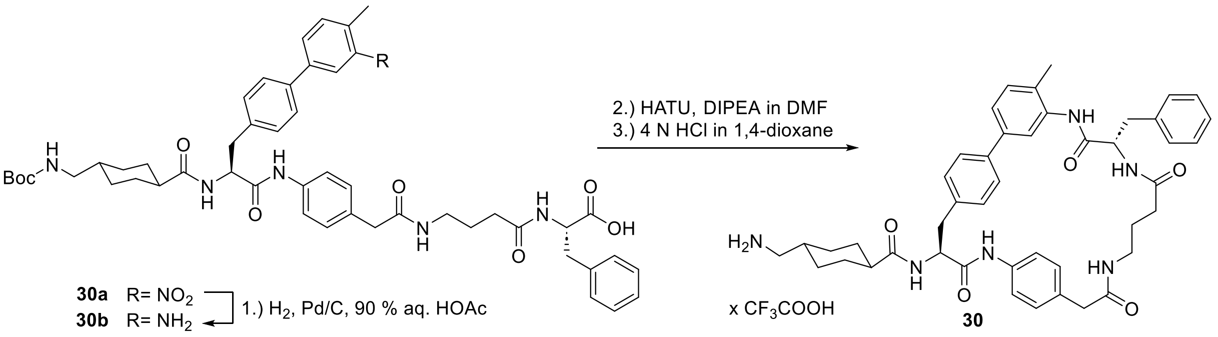

The synthesis was performed as described for inhibitor **33** in the main manuscript with slight variations. The linear precursor was synthesized on 200 mg 2-CTC resin (0.32 mmol absolute) using Fmoc-Phe-OH, Fmoc-Gaba-OH, Fmoc-4-aPha-OH **(S10)**, Fmoc-Bpa(4’-Me,3’-NO_2)-OH **(S4)**, and Boc-Txa-OH **(S2)**. After mild acidic cleavage from resin, the crude compound **30a** (HPLC method A: 30.79 min) was hydrogenated, the catalyst was removed by filtration, and the solvent was evaporated. The remaining residue was dissolved in 2 mL methanol and the product precipitated by addition of diethyl ether. The precipitate was washed twice with diethylether and dried *in vacuo* yielding crude intermediate **30b** (75 mg, 0.09 mmol colorless

solid, 27 %, HPLC method A: 21.80 min, purity > 95 %, MS calcd.: 874.46, m/z found: 875.29 $[M+H]^+$).

Compound **30b** was cyclized as described before. Afterwards, the solvent was removed and the remaining crude Tfa-protected derivative **11c** (structure not shown, HPLC method A: 29.19 min) was treated with 4 N HCl in dioxane for 1 h at room temperature. The final inhibitor **30** was purified by preparative HPLC (25 mg, 0.029 mmol colorless lyophilized solid, 33 % over two steps, HPLC method B: 22.52 min start at 20 % B, purity > 98 %, MS calcd.: 756.40, m/z found: 757.56 $[M+H]^+$).

Inhibitor 31

(1r,4S)-4-(Aminomethyl)-N-((4S,16S)-14-methyl-5,9,14,17-tetraoxo-16-phenethyl-6,10,15,18-tetraaza-1(1,3),2,7(1,4)-tribenzenacyclooctadecaphane-4-yl)cyclohexane-1-carboxamide × TFA or H-Txa-Bpa(4'-Me,*c*[3'-NH)-4-aPhac-Gaba-hPhe] × TFA

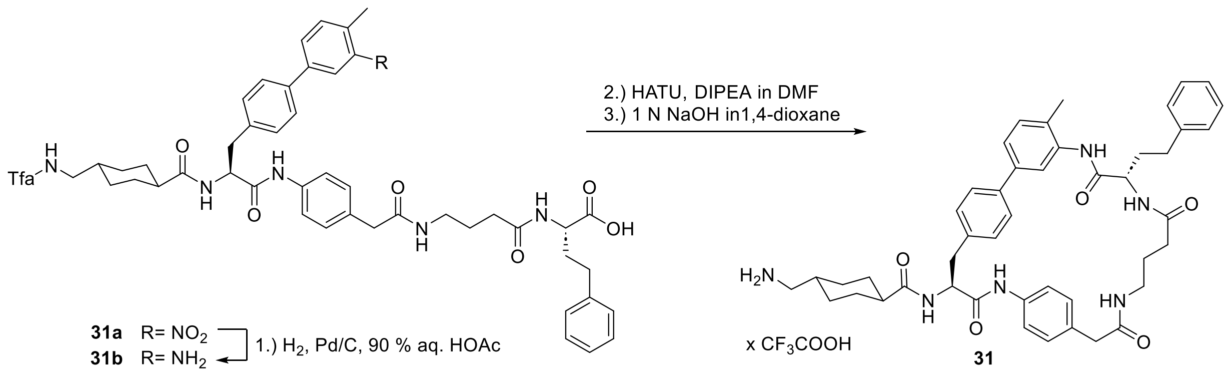

The synthesis was performed as described for inhibitor **33** in the main manuscript with slight variations. The linear precursor was synthesized on 150 mg 2-CTC resin (0.24 mmol absolute) using Fmoc-HPhe-OH, Fmoc-Gaba-OH, Fmoc-4-aPha-OH **(S10)**, Fmoc-Bpa(4'-Me,3'-NO_2)-OH **(S4)**, and Tfa-Txa-OH **(S1)**. After mild acidic cleavage from resin, the crude compound **31a** (HPLC method A: 31.85 min, MS calcd.: 762.32, m/z found: 761.45 $[M-H]^-$) was hydrogenated. After filtration of the catalyst and evaporation of the solvent, the remaining residue was dissolved in methanol and precipitated by addition of water (containing 0.1 % TFA). This precipitation procedure was repeated thrice, the precipitate was suspended in water and lyophilized yielding the crude intermediate 3**1b** (13 mg, 0.15 mmol colorless solid, 61 %, HPLC method A: 22.31 min, purity > 94 %, MS calcd.: 884.41, m/z found: 885.50 $[M+H]^+$).

Compound **31b** was cyclized as described before. Afterwards, the solvent was removed and the remaining crude Tfa-protected derivative **31c** (structure not shown, HPLC method A: 46.61 min) was treated with a mixture of 1 N aq. NaOH, 1,4-dioxane, THF, and acetone as solvent for 1 h at rt to cleave the Tfa-protecting group. The final inhibitor **31** was purified by preparative HPLC (9 mg, 0.01 mmol colorless lyophilized solid, 7 % over two steps, HPLC method B: 32.60 min, purity > 97 %, MS calcd.: 770.42, m/z found: 771.77 $[M+H]^+$.

Inhibitor 32

(S)-4-((1r,4S)-4-(Aminomethyl)cyclohexane-1-carboxamido)-5,9,14,17-tetraoxo-N-phenyl-6,10,15,18-tetraaza-1(1,3),2,7(1,4)-tribenzenacyclooctadecaphane-14-carboxamide × TFA
or H-Txa-Bpa(4'-Pha, *c*[3'-NH)-4-aPhac-Gaba-Gly] × TFA

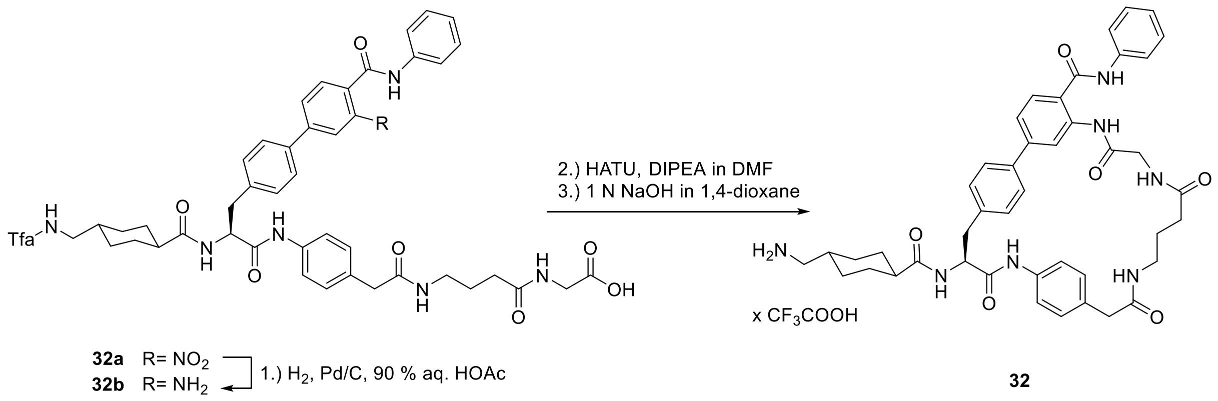

The synthesis was performed as described for inhibitor **33** in the main manuscript. The linear precursor was synthesized on 150 mg 2-CTC resin (0.24 mmol absolute) using Fmoc-Gly-OH, Fmoc-Gaba-OH, Fmoc-4-aPha-OH **(S10)**, Fmoc-Bpa(4'-Pha,3'-NO_2)-OH **(S4)**, and Tfa-Txa-OH **(S1)**. After mild acidic cleavage from resin, the crude compound **32a** (HPLC method A: 25.15 min, MS calcd.: 915.34, m/z found: 914.50 $[M-H]^-$) was hydrogenated and the amine was purified by preparative HPLC yielding intermediate **32b** (108 mg, 0.12 mmol colorless solid, 51 %, HPLC method A: 23.96 min, purity > 98 %, MS calcd.: 885.37, m/z found: 886.50 $[M+H]^+$), ^{1}H NMR (500 MHz, DMSO-d_6): δ[ppm]= 12.44 (bs, 1H), 10.23 – 9.80 (m, 2H), 9.34 (t, ^3J = 5.9 Hz, 1H), 8.12 (t, ^3J = 5.9 Hz, 1H), 8.06 (d, ^3J = 8.3 Hz, 1H), 7.96 (t, ^3J = 5.6 Hz, 1H), 7.75 – 7.70 (m, 3H), 7.55 (d, ^3J = 8.3 Hz, 2H), 7.50 (d, ^3J = 8.6 Hz, 2H), 7.37 (d, ^3J = 8.3 Hz, 2H), 7.36 – 7.31 (m, 2H), 7.18 (d, ^3J = 8.6 Hz, 1H), 7.08 (tt, ^3J = 7.6 Hz, ^4J = 1.2 Hz, 1H), 7.05 (d, ^4J = 1.8 Hz, 1H), 6.90 (dd, ^3J = 8.3 Hz, ^4J = 1.8 Hz, 1H), 4.69 (td, ^3J = 8.7 Hz, 5.1 Hz, 1H), 3.73 (d, ^3J = 5.9 Hz, 2H), 3.34 (s, 2H), 3.14 – 2.98 (m, 5H), 2.91 (dd, ^2J = 13.7 Hz,

^{3}J = 9.6 Hz, 1H), 2.19 – 2.07 (m, 3H), 1.77 – 1.56 (m, 6H), 1.49 – 1.39 (m, 1H), 1.32 – 1.12 (m, 2H), 0.94 – 0.81 (m, 2H). ^{13}C NMR (126 MHz, DMSO-D_6): δ[ppm]= 175.60, 172.72, 171.91, 170.68, 170.63, 168.05, 156.89 (pd, $^{2}J_{C\text{-}F}$ = 35.9 Hz), 143.95, 139.79, 138.23, 138.14, 137.67, 132.07, 130.33, 129.93, 129.73, 129.02, 126.69, 123.92, 121.10, 117.72, 115.43, 114.90, 114.71, 114.03, 54.91, 45.66, 44.04, 42.37, 41.10, 41.02, 40.61, 38.87, 36.92, 33.15, 29.95, 29.84, 29.08, 28.91, 25.87.

Compound **32b** was cyclized as described before. Afterwards, the solvent was removed and the remaining crude Tfa-protected derivative **32c** (structure not shown, HPLC method A: 25.76 min) was treated with a mixture of 1 N aq. NaOH, 1,4-dioxane, THF, and acetone as solvent for 1 h at rt to cleave the Tfa-protecting group. The final inhibitor **32** was purified by preparative HPLC (11 mg, 0.012 mmol colorless lyophilized solid, 10 % over two steps, HPLC method B: 18.93 min start at 20 % solvent B, purity > 97 %, MS calcd.: 771.39, m/z found: 772.48 $[M+H]^+$).

Inhibitor 33

The synthesis of inhibitor **33** is described in the main manuscript

Inhibitor 34

(1r,4S)-N-((4S,15R)-15-amino-14-methyl-5,9,16-trioxo-6,10,17-triaza-1(1,3),2,7(1,4)-tribenzenacycloheptadecaphane-4-yl)-4-(aminomethyl)cyclohexane-1-carboxamide × 2 TFA

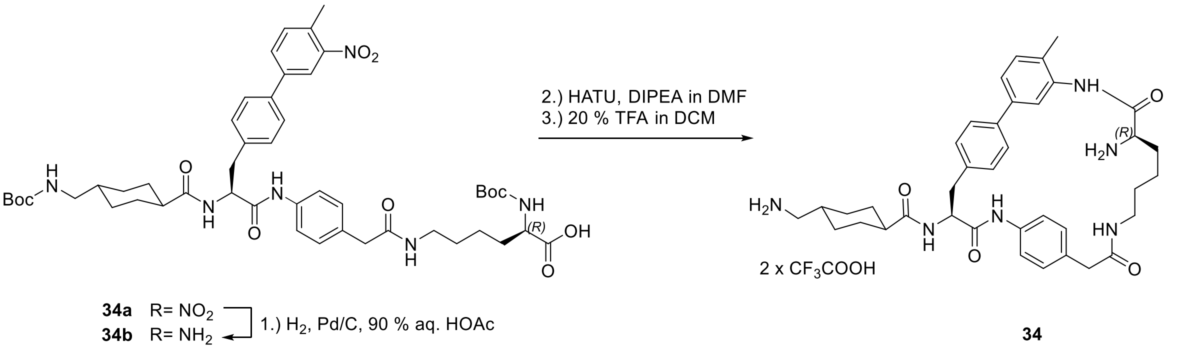

The synthesis was performed as described for inhibitor **33** in the main manuscript. The linear precursor was synthesized on 120 mg 2-CTC resin (0.19 mmol absolute) using Boc-DLys(Fmoc)-OH, Fmoc-4-aPha-OH **(S10)**, Fmoc-Bpa(4'-Me,3'-NO_2)-OH **(S4)**, and Boc-Txa-OH **(S2)**. After mild acidic cleavage from resin, the crude compound **34a** (HPLC method A:

32.54 min, MS calcd.: 900.46, m/z found: 901.43 $[M+H]^+$) was hydrogenated and the amine was purified by preparative HPLC yielding intermediate **34b** (72 mg, 0.084 mmol colorless solid, 43 %, HPLC method A: 23.51 min, purity > 99 %, MS calcd.: 870.49, m/z found: 869.71 $[M-H]^-$).

Compound **34b** was cyclized as described before. Afterwards, the solvent was removed and the remaining crude Boc-protected derivative **34c** (structure not shown, HPLC method A: 30.66 min) was treated with 4 mL 20 % TFA in DCM and the product precipitated in diethyl ether. The final inhibitor **34** was purified by preparative HPLC (13 mg, 0.015 mmol colorless lyophilized solid, 24 % over two steps, HPLC method A: 14.12 min, purity > 98 %, MS calcd.: 652.37, m/z found: 653.47 $[M+H]^+$).

Inhibitor 35

(1r,4S)-N-((4S,15S)-15-amino-14-methyl-5,9,16-trioxo-6,10,17-triaza-1(1,3),2,7(1,4)-tribenzenacycloheptadecaphane-4-yl)-4-(aminomethyl)cyclohexane-1-carboxamide × 2 TFA

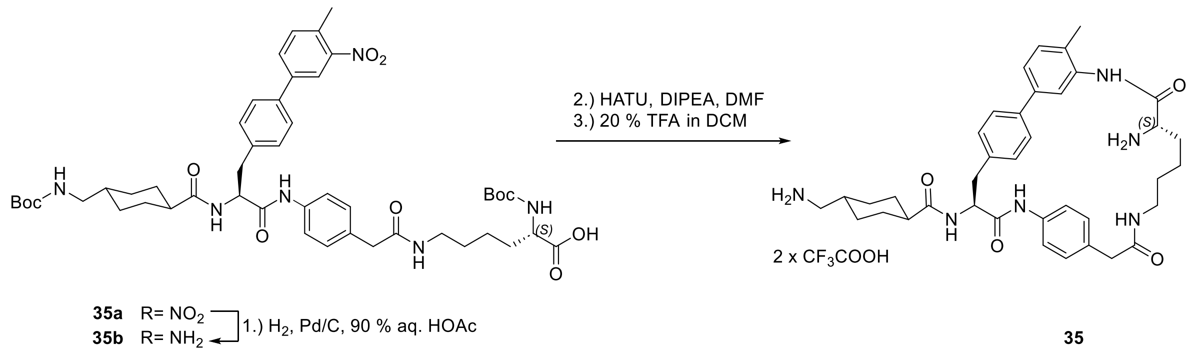

The synthesis was performed as described for inhibitor **33** in the main manuscript. The linear precursor was synthesized on 120 mg 2-CTC resin (0.19 mmol absolute) using Boc-Lys(Fmoc)-OH, Fmoc-4-aPha-OH **(S10)**, Fmoc-Bpa(4’-Me,3’-NO_2)-OH **(S4)**, and Boc-Txa-OH **(S2)**. After mild acidic cleavage from resin, the crude compound **35a** (HPLC method A: 32.79 min, MS calcd.: 900.46, m/z found: 901.54 $[M+H]^+$) was hydrogenated and the amine was purified by preparative HPLC yielding intermediate **35b** (97 mg, 0.11 mmol colorless solid, 58 %, HPLC method A: 23.82 min, purity > 98 %, MS calcd.: 870.49, m/z found: 871.74 $[M-H]^+$).

Compound **35b** was cyclized as described before. Afterwards, the solvent was removed and the remaining crude Boc-protected derivative **35c** (structure not shown, HPLC method A: 30.71 min) was treated with 4 mL 20 % TFA in DCM and the product precipitated in diethyl ether.

The final inhibitor **35** was purified by preparative HPLC (49 mg, 0.056 mmol colorless lyophilized solid, 50 % over two steps, HPLC method A: 14.08 min, purity > 98 %, MS calcd.: 652.37, m/z found: 653.56 $[M+H]^+$).

Inhibitor 36

(1r,4S)-4-(aminomethyl)-N-((S)-14-methyl-5,9,14-trioxo-6,15-diaza-10(1,4)-piperazina-1(1,3),2,7(1,4)-tribenzenacyclopentadecaphane-4-yl)cyclohexane-1-carboxamide × 2 TFA

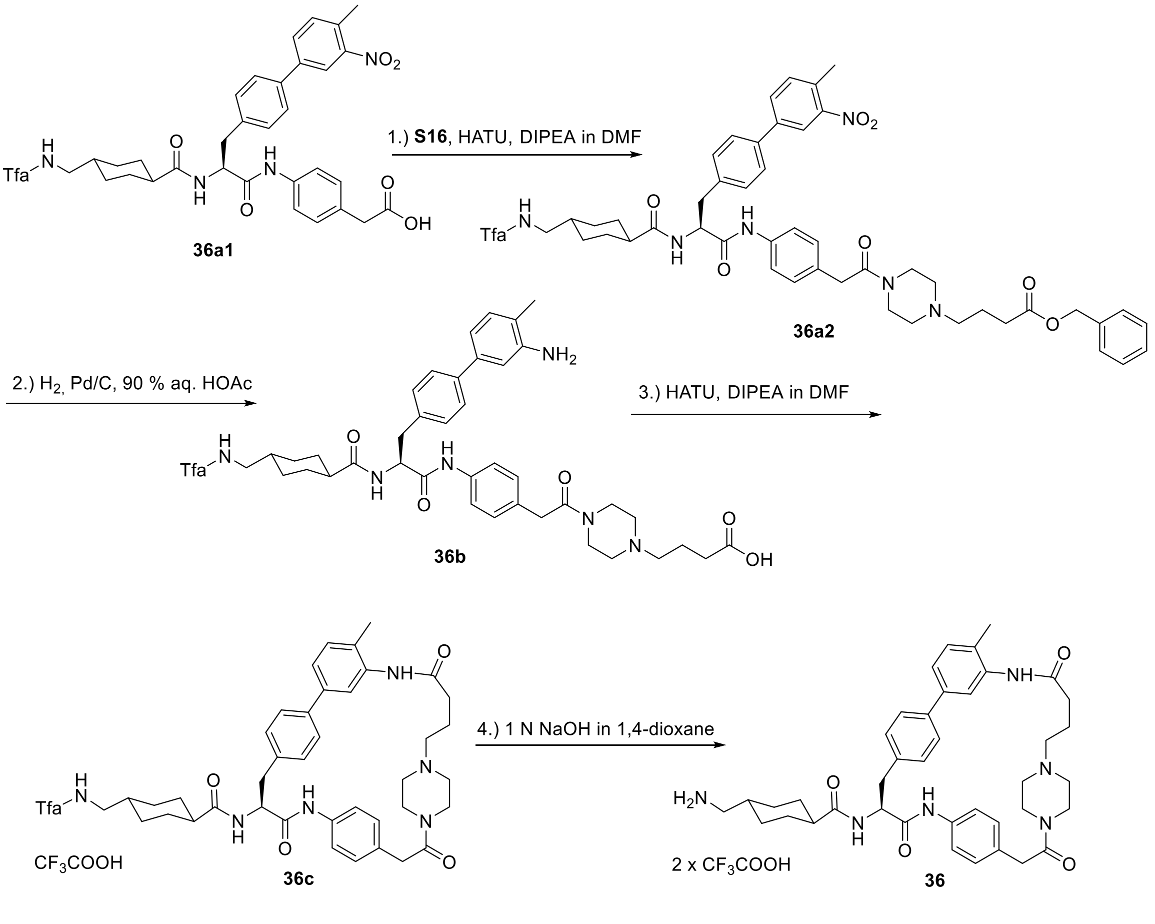

Inhibitor **36** was prepared with slight modifications (see scheme above). The linear precursor was synthesized on 150 mg 2-CTC resin (0.24 mmol absolute) using Fmoc-4-aPha-OH **(S10)**, Fmoc-Bpa(4'-Me,3'-NO_2)-OH **(S4)**, and Tfa-Txa-OH **(S1)**. After mild acidic cleavage from resin and evaporation of the solvent, the crude compound **36a1** was obtained (128 mg, 0.19 mmol, 1 eq.). It was dissolved in 5 mL DMF and treated with intermediate **S16** (93.2 mg, 0.19 mmol, 1 eq.), DIPEA (145 µL, 0.86 mmol, 4.5 eq.), and HATU (73 mg, 0.19 mmol, 1 eq.). The mixture was stirred 1 h at 0 °C and 30 min at room temperature. The solvent was removed *in vacuo* providing the crude intermediate **36a2**, which was hydrogenated. The amine was purified by preparative HPLC yielding intermediate **36b** (81 mg, 0.089 mmol colorless solid,

37 %, HPLC method A: 17.42 min, purity > 97 %, MS calcd.: 792.38, m/z found: 793.38 $[M+H]^+$), ^{1}H NMR (500 MHz, DMSO-d_6): δ[ppm]= 10.07 (s, 1H), 9.35 (t, ^3J = 5.7 Hz, 1H), 8.07 (d, ^3J = 8.2 Hz, 1H), 7.53 (d, ^3J = 8.6 Hz, 2H), 7.49 (d, ^3J = 8.3 Hz, 2H), 7.35 (d, ^3J = 8.3 Hz, 2H), 7.23 – 7.06 (m, 5H), 4.66 (td, ^3J = 8.5 Hz, 5.0 Hz), 3.71 (s), 3.14 – 3.08 (m), 3.06 – 3.00 (m), 2.89 (dd, ^2J = 13.8 Hz, ^3J = 9.7 Hz, 2.33 (t, ^3J = 7.2 Hz, 2H), 2.19 (s, 3H), 2.17 – 2.09 (m, 1H), 1.92 – 1.82 (m, 2H), 1.76 – 1.56 (m, 4H), 1.49 – 1.38 (m, 1H), 1.30 – 1.12 (m, 2H), 0.94 – 0.80 (m, 2H). Together with the water content of the solvent, the signals caused by piperazine form two very broad signals between approx. 5.5 and 2.5 ppm. The overlapping signals that could be clearly distinguished in this region are listed without integrals. ^{13}C NMR (126 MHz, DMSO-d_6): δ[ppm]= 175.07, 173.36, 170.27, 169.24, 156.31 (pd, $^2J_{C\text{-}F}$ = 36.0 Hz), 138.48, 137.92, 137.29, 136.87, 131.01, 130.19, 129.71, 129.31, 125.83, 119.36, 117.14, 115.78, 115.35, 114.85, 54.96, 54.46, 50.96, 50.66, 45.07, 43.45, 42.25, 38.46, 38.19, 37.30, 36.34, 30.42, 29.35, 29.25, 28.50, 28.32, 18.85, 16.76.

Compound **36b** was cyclized as described before. Afterwards, the solvent was removed and the remaining crude Tfa-protected cyclic derivative was purified by preparative HPLC yielding intermediate **36c** (HPLC method A: 22.06 min, MS calcd.: 774.37, m/z found: 775.30 $[M+H]^+$). The Tfa group was removed by treating intermediate **36c** with a mixture of 1 N aq. NaOH and 1,4-dioxane as solvent for 1 h at rt to cleave the Tfa-protecting group. After 2 h stirring, the mixture was neutralized by addition of TFA and the product purified by preparative HPLC (28 mg, 0.032 mmol colorless lyophilized solid, 36 %, HPLC method B: 22.46 min start at 10 % solvent B, purity > 97 %, MS calcd.: 678.39, m/z found: 679.34 $[M+H]^+$).

Inhibitor 37

(1r,4S)-4-(Aminomethyl)-N-((S)-24-methyl-4,8,12-trioxo-3,11-diaza-7(1,4)-piperazina-1,10(1,4),2(1,3)-tribenzenacyclotetradecaphane-13-yl)cyclohexane-1-carboxamide × 2 TFA

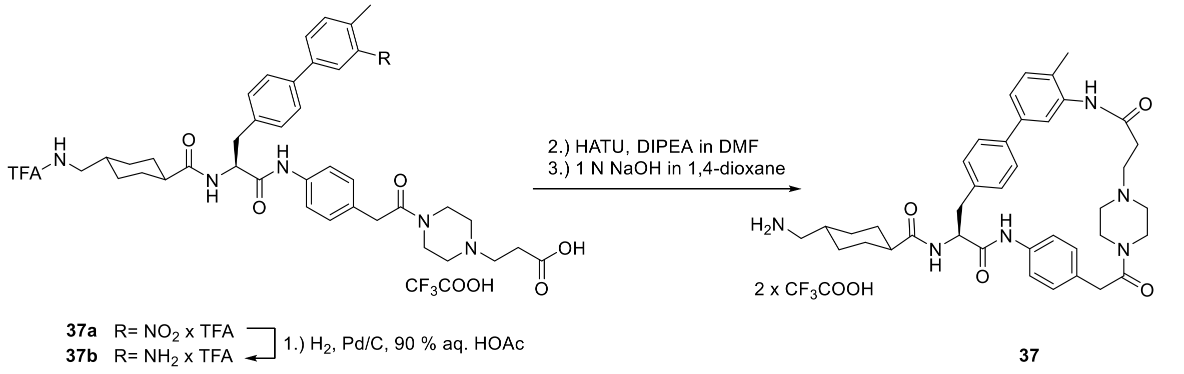

The synthesis was performed as described for inhibitor **33** in the main manuscript. The linear precursor was synthesized on 180 mg 2-CTC resin (0.29 mmol absolute) using intermediate **S14**, Fmoc-4-aPha-OH **(S10)**, Fmoc-Bpa(4'-Me,3'-NO_2)-OH **(S4)**, and Tfa-Txa-OH **(S1)**. After mild acidic cleavage from resin, the crude compound **37a** (HPLC method A: 24.07 min, MS calcd.: 808.34 m/z found: 809.32 $[M+H]^+$) was hydrogenated and the amine was purified by preparative HPLC yielding intermediate **37b** (168 mg, 0.19 mmol colorless solid, 65 %, HPLC method A: 16.24 min, purity > 98 %, MS calcd.: 778.37, m/z found: 779.32 $[M+H]^+$). ^{1}H NMR (500 MHz, DMSO-d_6): δ[ppm]= 10.07 (s, 1H), 9.35 (t, ^3J = 5.8 Hz, 1H), 8.07 (d, ^3J = 8.2 Hz, 1H), 7.53 (d, ^3J = 8.6 Hz, 2H), 7.48 (d, ^3J = 8.3 Hz, 2H), 7.34 (d, ^3J = 8.3 Hz, 2H), 7.18 – 7.10 (m, 4H), 7.04 (d, ^3J = 8.0 Hz, 1H), 4.66 (td, ^3J = 8.8 Hz, 5.0 Hz, 1H), 3.71 (s), 3.33 (t, ^3J = 7.4 Hz, 2H), 3.23 (bs, 4H), 3.15 – 2.98 (m, 3H), 2.89 (dd, ^2J = 13.8 Hz, ^3J = 9.6 Hz, 1H), 2.75 (t, ^3J = 7.4 Hz, 2H), 2.17 (s, 3H), 2.16 – 2.06 (m, 1H), 1.76 – 1.55 (m, 4H), 1.48 – 1.37 (m, 1H), 1.31 – 1.12 (m, 2H), 0.96 – 0.81 (m, 2H). Together with the water content of the solvent, one of the two the signals caused by piperazine forms a very broad signals between approx. 4.5 and 3.25 ppm. Therefor, the overlapping singlet at 3.71 ppm is listed without integral. ^{13}C NMR (126 MHz, DMSO-d_6): δ[ppm]= 175.05, 171.43, 170.27, 169.24, 156.30 (pd, $^2J_{C-F}$ = 35.9 Hz), 156.30 (pd, $^2J_{C-F}$ = 35.9 Hz), 138.42, 138.07, 137.29, 136.77, 135.04, 130.90, 130.18, 129.68, 129.31, 125.80, 119.35, 117.69, 117.13, 114.84, 54.44, 51.35, 51.13, 50.92, 45.06, 43.44, 42.20, 38.43, 38.19, 37.29, 36.33, 29.34, 29.24, 28.53, 28.49, 28.32, 16.80.

Compound **37b** was cyclized as described before. Afterwards, the solvent was removed and the remaining crude Tfa-protected derivative **37c** (structure not shown, HPLC method A: 22.16 min, MS calcd.: 760.86 m/z found: 761.41 $[M+H]^+$) was treated with a mixture of 1 N aq. NaOH

and 1,4-dioxane as solvent for 1 h at rt to cleave the Tfa-protecting group. The final inhibitor **37** was purified by preparative HPLC (98 mg, 0.11 mmol colorless lyophilized solid, 58 % over two steps, HPLC method A: 14.86 min, purity > 98 %, MS calcd.: 664.37, m/z found: 665.38 $[M+H]^+$).

Inhibitor 38

(1r,4S)-4-(Aminomethyl)-N-((S)-24-methyl-4,7,11-trioxo-3,10-diaza-6(1,4)-piperazina-1,9(1,4),2(1,3)-tribenzenacyclotridecaphane-12-yl)cyclohexane-1-carboxamide × 2 TFA

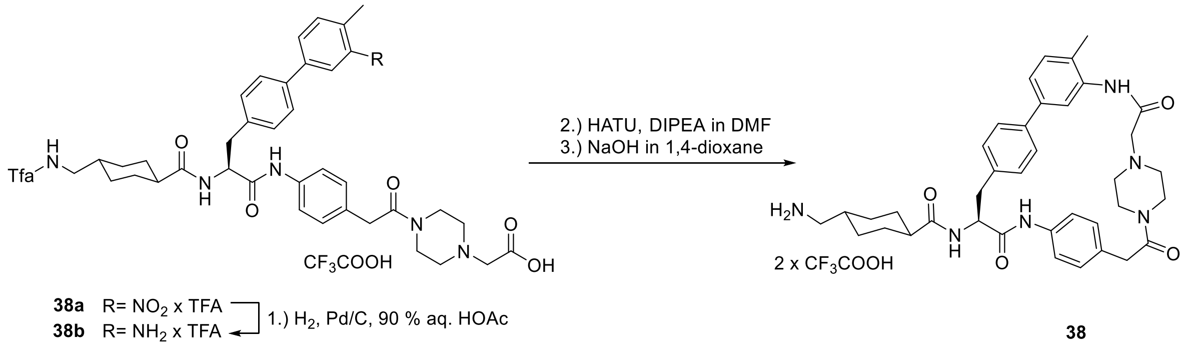

The synthesis was performed as described for inhibitor **33** in the main manuscript. The linear precursor was synthesized on 200 mg 2-CTC resin (0.32 mmol absolute) using intermediate **S13**, Fmoc-4-aPha-OH **(S10)**, Fmoc-Bpa(4'-Me,3'-NO_2)-OH **(S4)**, and Tfa-Txa-OH **(S1)**. After mild acidic cleavage from resin, the crude compound **38a** (HPLC method A: 24.14 min, MS calcd.: 794.33, m/z found: 795.40 $[M+H]^+$) was hydrogenated and the amine was purified by preparative HPLC yielding intermediate **38b** (148 mg, 0.17 mmol colorless solid, 52 %, HPLC method A: 15.89 min, purity > 98 %, MS calcd.: 764.35, m/z found: 765.42 $[M+H]^+$). ^{1}H NMR (500 MHz, DMSO-d_6): δ[ppm] = 10.07 (s, 1H), 9.35 (t, ^3J = 5.8 Hz, 1H), 8.07 (d, ^3J = 8.3 Hz, 1H), 7.53 (d, ^3J = 8.6 Hz, 2H), 7.48 (d, ^3J = 8.3 Hz, 2H), 7.35 (d, ^3J = 8.3 Hz, 2H), 7.18 – 7.12 (m, 4H), 7.06 (d, ^3J = 7.5 Hz, 1H), 4.66 (td, ^3J = 8.4 Hz, 5.1 Hz, 1H), 4.08 (s, 2H), 3.81 – 3.61 (m, 6H), 3.22 (s, 4H), 3.09 – 2.99 (m, 3H), 2.89 (dd, ^2J = 13.8 Hz, ^3J = 9.7 Hz, 1H), 2.18 (s, 3H), 2.16 – 2.09 (m, 1H), 1.74 – 1.54 (m, 4H), 1.48 – 1.37 (m, 1H), 1.30 – 1.08 (m, 2H), 0.98 – 0.77 (m, 2H). ^{13}C NMR (126 MHz, DMSO-d_6): δ[ppm] = 175.05, 170.27, 169.20, 167.44, 158.16, 157.90, 157.90, 157.63, 156.44, 156.16, 138.43, 138.03, 137.30, 136.80, 130.93, 130.17, 129.68, 129.28, 125.81, 119.36, 55.27, 55.27, 54.43, 51.69, 51.47, 45.06, 43.43, 42.09, 38.45, 38.04, 36.33, 29.34, 29.24, 28.49, 28.31, 16.78.

Compound **38b** was cyclized as described before. Afterwards, the solvent was removed and the remaining crude Tfa-protected derivative **38c** (structure not shown, HPLC method A: 20.05 min, MS calcd.: 714.34, m/z found: 715.20 [M+H]+) was treated with a mixture of 1 N aq. NaOH, 1,4-dioxane and DMSO. The final inhibitor **38** was purified by preparative HPLC (65 mg, 0.075 mmol colorless lyophilized solid, 44 % over two steps, HPLC method A: 13.91 min, purity > 99 %, MS calcd.: 650.36, m/z found: 651.37 $[M+H]^+$).

Inhibitor 39

(1r,4S)-4-(Aminomethyl)-N-((S)-14-methyl-5,9,15,17-tetraoxo-6,10,16,19-tetraaza-1,7(1,3),2(1,4)-tribenzenacyclononadecaphane-4-yl)cyclohexane-1-carboxamide × TFA
or H-Txa-Bpa(4'-Me,*c*[3'-NH)-3-aPhac-Ava-Gly] × TFA

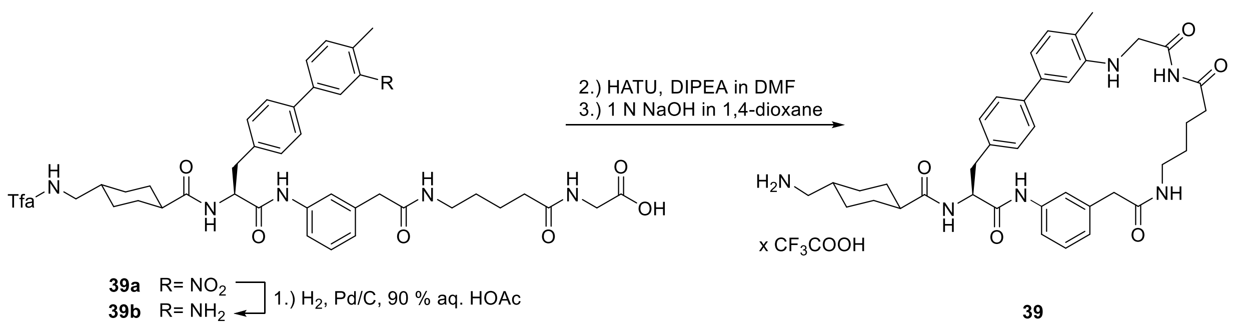

The synthesis was performed as described for inhibitor **33** in the main manuscript. The linear precursor was synthesized on 130 mg 2-CTC resin (0.208 mmol absolute) using Fmoc-Gly-OH, Fmoc-5-Ava-OH, Fmoc-3-aPha-OH **(S9)**, Fmoc-Bpa(4'-Me,3'-NO_2)-OH **(S4)**, and Tfa-Txa-OH **(S1)**. After mild acidic cleavage from resin, the crude compound **39a** (HPLC method A: 26.08 min) was hydrogenated and the amine was purified by preparative HPLC yielding intermediate **39b** (84 mg, 0.11 mmol colorless solid, 51 %, HPLC method A: 18.80 min, purity > 99 %, MS calcd.: 794.36, m/z found: 795.39 $[M+H]^+$). ^{1}H NMR (500 MHz, DMSO-d_6): δ[ppm] = 10.05 (s, 1H), 9.34 (t, ^3J = 5.9 Hz, 1H), 8.08 (t, ^3J = 5.9 Hz, 1H), 8.04 (d, ^3J = 8.3 Hz, 1H), 8.00 (t, ^3J = 5.6 Hz, 1H), 7.51 – 7.45 (m, 4H), 7.36 (d, ^3J = 8.3 Hz, 2H), 7.24 – 7.12 (m, 4H), 6.94 (d, ^3J = 7.6 Hz, 1H), 4.67 (td, ^3J = 8.5 Hz, 4.9 Hz, 1H), 3.72 (d, ^3J = 5.9 Hz, 2H), 3.36 (s, 2H), 3.11 – 3.00 (m, 5H), 2.89 (dd, ^2J = 13.8 Hz, ^3J = 9.7 Hz, 1H), 2.20 (s, 3H), 2.15 – 2.08 (m, 3H), 1.76 – 1.57 (m, 4H), 1.56 – 1.46 (m, 2H), 1.44 – 1.36 (m, 3H), 1.31 – 1.10 (m, 2H), 0.97 – 0.78 (m, 2H). ^{13}C NMR (126 MHz, DMSO-d_6): δ[ppm] = 175.01, 172.35, 171.33, 170.25, 169.68, 158.14, 138.67, 138.53, 137.79, 137.76, 137.04, 136.97, 131.08, 129.73,

128.41, 127.09, 125.82, 124.06, 119.90, 117.39, 117.13, 54.41, 45.06, 43.45, 42.37, 40.48, 38.39, 37.30, 36.32, 34.63, 29.35, 29.25, 28.62, 28.49, 28.31, 22.62, 16.73.

Compound **39b** was cyclized as described before. Afterwards, the solvent was removed and the remaining crude Tfa-protected derivative **39c** (structure not shown, HPLC method A: 24.86 min) was treated with a mixture of 1 N aq. NaOH, 1,4-dioxane and acetone as solvent for 1 h at rt to cleave the Tfa-protecting group. The final inhibitor **39** was purified by preparative HPLC (30 mg, 0.038 mmol colorless lyophilized solid, 36 % over two steps, HPLC method A: 16.86 min, purity > 99 %, MS calcd.: 680.37, m/z found: 681.43 $[M+H]^+$).

Inhibitor 40

(1r,4S)-4-(Aminomethyl)-N-((S)-14-methyl-5,9,14,17-tetraoxo-6,10,15,18-tetraaza-1,7(1,3),2(1,4)-tribenzenacyclooctadecaphane-4-yl)cyclohexane-1-carboxamide × TFA
or H-Txa-Bpa(4'-Me,*c*[3'-NH)-3-aPhac-Gaba-Gly] × TFA

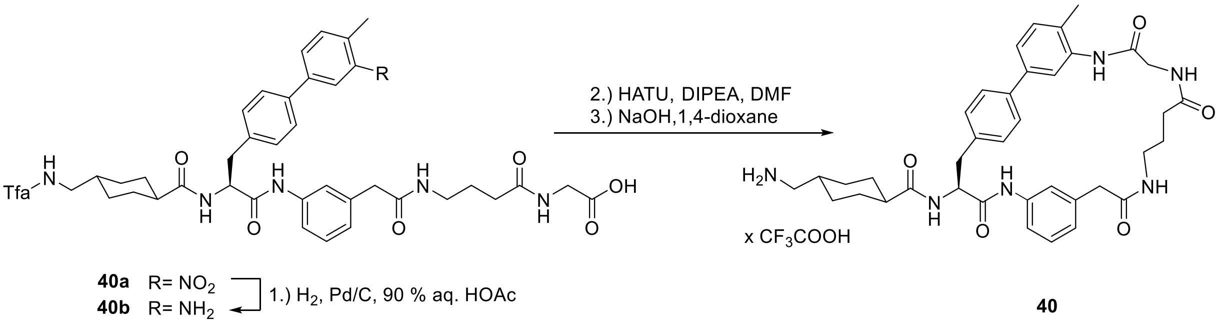

The synthesis was performed as described for inhibitor **33** in the main manuscript. The linear precursor was synthesized on 130 mg 2-CTC resin (0.21 mmol absolute) using Fmoc-Gly-OH, Fmoc-Gaba-OH, Fmoc-3-aPha-OH **(S9)**, Fmoc-Bpa(4'-Me,3'-NO_2)-OH **(S4)**, and Tfa-Txa-OH **(S1)**. After mild acidic cleavage from resin, the crude compound **40a** (HPLC method A: 26.69 min) was hydrogenated and the amine was purified by preparative HPLC yielding intermediate **40b** (70 mg, 0.09 mmol, 43 %, HPLC method A: 18.88 min, purity > 99 %, MS calcd.: 780.35, m/z found: 781.30 $[M+H]^+$, ^{1}H NMR (500 MHz, DMSO-d_6): δ[ppm] = 10.06 (s, 1H), 9.34 (t, ^3J = 5.8 Hz, 1H), 8.12 (t, ^3J = 5.8 Hz, 1H), 8.09 – 8.00 (m, 2H), 7.52 – 7.45 (m, 4H), 7.36 (d, ^3J = 8.3 Hz, 2H), 7.28 – 7.13 (m, 4H), 6.95 (d, ^3J = 7.8 Hz, 1H), 4.67 (td, ^3J = 8.9 Hz, 4.9 Hz, 1H), 3.73 (d, J = 5.9 Hz, 2H), 3.37 (s, 2H), 3.12 – 2.97 (m, 5H), 2.89 (dd, ^2J = 13.8 Hz, ^3J = 9.8 Hz, 1H), 2.21 (s, 3H), 2.19 – 2.05 (m, 3H), 1.77 – 1.53 (m, 6H), 1.51 – 1.35 (m, 1H), 1.30 – 1.10 (m, 2H), 0.97 – 0.79 (m, 2H). 13C NMR (126 MHz, DMSO-d6):

δ[ppm] = 175.02, 172.14, 171.33, 170.27, 169.80, 158.05 (pd, 2JC-F = 34.3 Hz), 156.3 (pd, 2JC-F = 35.9 Hz), 138.69, 138.54, 137.74, 136.99, 131.11, 129.74, 128.43, 125.83, 124.07, 120.57, 119.90, 119.41, 117.41, 117.13, 54.42, 45.07, 43.45, 42.39, 40.52, 38.31, 37.29, 36.33, 32.56, 29.35, 29.25, 28.49, 28.31, 25.27, 16.72.

Compound **40b** was cyclized as described before. Afterwards, the solvent was removed and the remaining crude Tfa-protected derivative **40c** (structure not shown, HPLC method A: 24.35 min) was treated with a mixture of 1 N aq. NaOH, 1,4-dioxane and acetone. The final inhibitor **40** was purified by preparative HPLC (19 mg, 0.024 mmol colorless lyophilized solid, 27 % over two steps, HPLC method A: 16.65 min, purity > 99 %, MS calcd.: 666.35, m/z found: 667.37 $[M+H]^+$).

Inhibitor 41

(1r,4S)-4-(aminomethyl)-N-((S)-14-methyl-5,9,13,16-tetraoxo-6,10,14,17-tetraaza-1,7(1,3),2(1,4)-tribenzenacycloheptadecaphane-4-yl)cyclohexane-1-carboxamide × TFA

or H-Txa-Bpa(4’-Me,*c*[3’-NH)-3-aPhac-βAla-Gly] × TFA

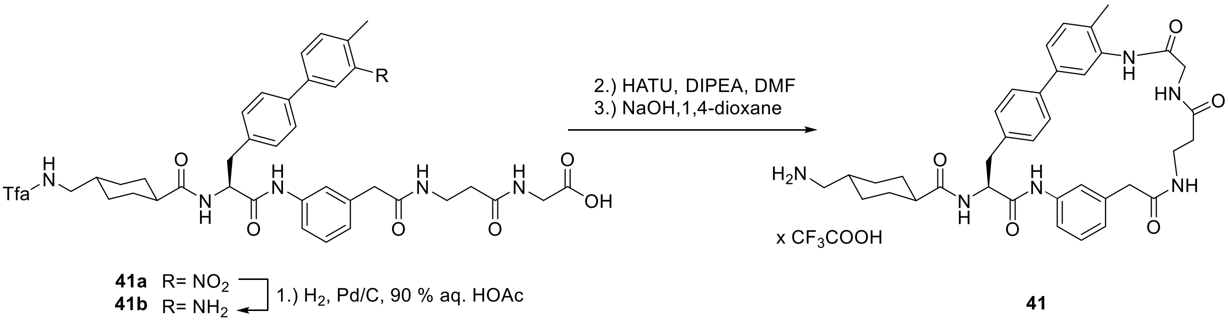

The synthesis was performed as described for inhibitor **33** in the main manuscript. The linear precursor was synthesized on 150 mg 2-CTC resin (0.24 mmol absolute) using Fmoc-Gly-OH, Fmoc-β-Ala-OH, Fmoc-3-aPha-OH **(S9)**, Fmoc-Bpa(4’-Me,3’-NO_2)-OH **(S4)**, and Tfa-Txa-OH **(S1)**. After mild acidic cleavage from resin, the crude compound **41a** (HPLC method A: 26.61 min) was hydrogenated and the amine was purified by preparative HPLC yielding intermediate **41b** (94 mg, 0.12 mmol colorless solid, 59 %, HPLC method A: 18.73 min, purity > 97 %, MS calcd.: 766.33, m/z found: 767.29 $[M+H]^+$, ^1H NMR (500 MHz, DMSO-d_6): δ[ppm] = 10.05 (s, 1H), 9.34 (t, ^3J = 5.7 Hz, 1H), 8.19 (t, ^3J = 5.7 Hz, 1H), 8.08 – 8.00 (m, 2H), 7.55 – 7.43 (m, 4H), 7.36 (d, ^3J = 8.1 Hz, 2H), 7.25 – 7.11 (m, 4H), 6.94 (d, ^3J = 7.5 Hz, 1H), 4.67 (td, ^3J = 8.9 Hz, 4.9 Hz, 1H), 3.74 (d, ^3J = 5.8 Hz, 2H), 3.36 (s, 2H), 3.28 – 3.20 (m, 2H), 3.09 – 2.98 (m, 3H), 2.88 (dd, ^2J = 13.7 Hz, ^3J = 9.6 Hz, 1H), 2.31 (t, ^3J = 7.2 Hz, 2H), 2.20 (s,

3H), 2.16 – 2.08 (m, 1H), 1.76 – 1.53 (m, 4H), 1.49 – 1.37 (m, 1H), 1.31 – 1.09 (m, 2H), 0.96 – 0.81 (m, 2H), ^{13}C NMR (126 MHz, DMSO-d_6): δ[ppm] = 175.03, 171.29, 170.69, 170.28, 169.89, 138.69, 138.52, 137.83, 136.96, 136.89, 131.06, 129.73, 128.43, 125.83, 119.94, 119.42, 117.41, 45.07, 43.45, 42.29, 40.52, 37.29, 36.33, 35.30, 35.02, 29.35, 29.25, 28.50, 28.30, 16.74, 16.42.

Compound **41b** was cyclized as described before. Afterwards, the solvent was removed and the remaining crude Tfa-protected derivative **41c** (structure not shown, HPLC method A: 24.21 min) was treated with a mixture of 1 N aq. NaOH, 1,4-dioxane, THF and acetone as solvent for 1 h at rt to cleave the Tfa-protecting group. The final inhibitor **41** was purified by preparative HPLC (34 mg, 0.044 mmol colorless lyophilized solid, 36 % over two steps, HPLC method A: 16.09 min, purity > 99 %, MS calcd.: 652.34, m/z found: 653.42 [M+H]$^+$).

Inhibitor 42

(1r,4S)-4-(Aminomethyl)-N-((S)-14-methyl-5,10,15,18-tetraoxo-6,11,16,19-tetraaza-1,8(1,3),2(1,4)-tribenzenacyclononadecaphane-4-yl)cyclohexane-1-carboxamide × TFA
or H-Txa-Bpa(4'-Me, c[3'-NH)-3-aMePhac-Gaba-Gly] × TFA

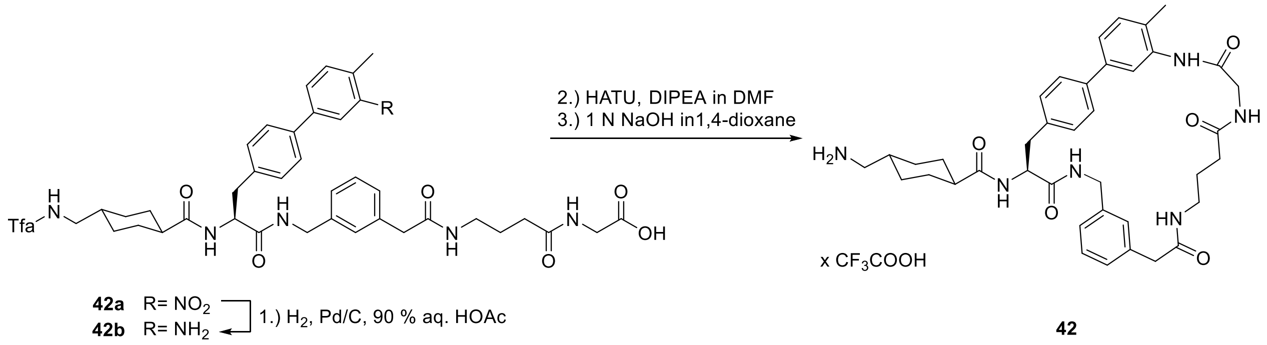

The synthesis was performed as described for inhibitor **33** in the main manuscript. The linear precursor was synthesized on 130 mg 2-CTC resin (0.21 mmol absolute) using Fmoc-Gly-OH, Fmoc-Gaba-OH, Fmoc-3-aPha-OH **(S10)**, Fmoc-Bpa(4'-Me,3'-NO_2)-OH **(S4)**, and Tfa-Txa-OH **(S1)**. After mild acidic cleavage from resin, the crude compound **42a** (HPLC method A: 26.89 min) was hydrogenated and the amine was purified by preparative HPLC yielding intermediate **42b** (87 mg, 0.11 mmol colorless solid, 53 %, HPLC method A: 18.54 min, purity > 97 %, MS calcd.: 794.36, m/z found: 795.23 [M+H]$^+$, ^{1}H NMR (500 MHz, DMSO-d_6): δ[ppm]= 9.33 (t, ^3J = 5.8 Hz, 1H), 8.42 (t, ^3J = 5.9 Hz, 1H), 8.11 (t, ^3J = 5.8 Hz, 1H), 8.00 (t, ^3J = 5.6 Hz, 1H), 7.94 (d, ^3J = 8.5 Hz, 1H), 7.47 (d, ^3J = 8.3 Hz, 2H), 7.29 (d, ^3J = 8.3 Hz, 2H), 7.24 – 7.05 (m, 6H), 7.00 (d, ^3J = 7.7 Hz, 1H), 4.55 (td, ^3J = 9.0 Hz, 5.1 Hz, 1H), 4.29 – 4.22

(m, 2H), 3.72 (d, ^3J = 5.9 Hz, 2H), 3.36 (s, 2H), 3.09 – 3.00 (m, 5H), 2.83 (dd, ^2J = 13.7 Hz, ^3J = 9.6 Hz, 1H), 2.20 (s, 3H), 2.14 – 2.05 (m, 3H), 1.76 – 1.53 (m, 6H), 1.48 – 1.37 (m, 1H), 1.30 – 1.20 (m, 1H), 1.14 (qd, $^3J_{ax.-ax.}$ = 12.9 Hz, $^3J_{ax.-eq.}$ = 3.3 Hz, 1H), 0.95 – 0.81 (m, 2H). 13C NMR (126 MHz, DMSO-d6): δ[ppm] = 174.84, 172.14, 171.32, 171.19, 169.83, 157.99 (pd, 2JC F = 34.4 Hz), 156.29 (pd, 2JC-F = 36.13 Hz), 139.06, 138.56, 137.71, 137.18, 136.36, 131.06, 129.71, 128.03, 127.86, 127.31, 125.79, 124.99, 117.13, 114.83, 53.66, 45.07, 43.51, 42.31, 42.00, 40.51, 38.30, 37.35, 36.33, 32.55, 29.38, 29.23, 28.54, 28.20, 25.26, 16.74.

Compound **42b** was cyclized as described before. Afterwards, the solvent was removed and the remaining crude Tfa-protected derivative **42c** (structure not shown, HPLC method A: 22.89 min) was treated with a mixture of 1 N aq. NaOH, 1,4-dioxane, THF and acetone as solvent for 1 h at rt to cleave the Tfa-protecting group. The final inhibitor **42** was purified by preparative HPLC (21 mg, 0.026 mmol colorless lyophilized solid, 24 % over two steps, HPLC method A: 16.51 min, purity > 99 %, MS calcd.: 680.37, m/z found: 681.44 $[M+H]^+$).

Inhibitor 43

(1r,4S)-4-(Aminomethyl)-N-((S)-14-methyl-5,8,14,17-tetraoxo-6,9,15,18-tetraaza-7(2,6)-naphthalena-1(1,3),2(1,4)-dibenzenacyclooctadecaphane-4-yl)cyclohexane-1-carboxamide × TFA or H-Txa-Bpa(4'-Me,*c*[3'-NH)-6-aNap-Ava-Gly] × TFA

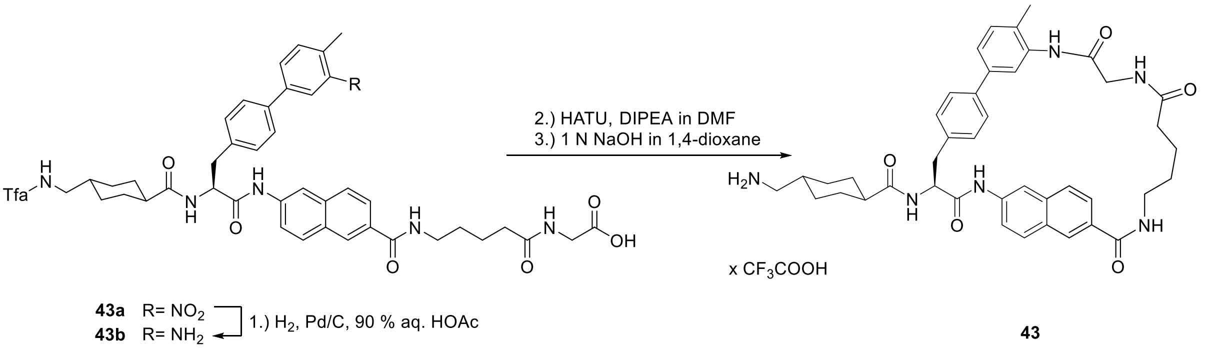

The synthesis was performed as described for inhibitor **33** in the main manuscript. The linear precursor was synthesized on 130 mg 2-CTC resin (0.21 mmol absolute) using Fmoc-Gly-OH, Fmoc-5-Ava-OH, Fmoc-6-aNap-OH **(S11)**, Fmoc-Bpa(4'-Me,3'-NO_2)-OH **(S4)**, and Tfa-Txa-OH **(S1)**. After mild acidic cleavage from resin, the crude compound **43a** (HPLC method A: 29.53 min) was hydrogenated and the amine was purified by preparative HPLC yielding intermediate **43b** (50 mg, 0.06 mmol, 30 %, HPLC method A: 20.47 min, purity > 97 %, MS calcd.: 830.36, m/z found: 831.45 $[M+H]^+$). ^{1}H NMR (500 MHz, DMSO-d_6): δ[ppm] = 10.39

(s, 1H), 9.35 (t, ^3J = 5.8 Hz, 1H), 8.54 (t, ^3J = 5.6 Hz, 1H), 8.37 – 8.32 (m, 2H), 8.15 (d, ^3J = 8.1 Hz, 1H), 8.11 (t, ^3J = 5.9 Hz, 1H), 7.96 (d, ^3J = 9.1 Hz, 1H), 7.89 – 7.83 (m, 2H), 7.66 (dd, ^3J = 8.9 Hz, ^4J = 2.0 Hz, 1H), 7.51 (d, ^3J = 8.3 Hz, 2H), 7.39 (d, ^3J = 8.3 Hz, 2H), 7.26 (s, 1H), 7.19 (s, 2H), 4.75 (td, ^3J = 8.7 Hz, 5.2 Hz, 1H), 3.73 (d, ^3J = 5.9 Hz, 2H), 3.30 (q, ^3J = 6.0 Hz, 2H), 3.12 (dd, ^2J = 13.9 Hz, ^3J = 5.0 Hz, 1H), 3.03 (t, ^3J = 6.3 Hz, 2H), 2.95 (dd, ^2J = 13.8 Hz, ^3J = 9.6 Hz, 1H), 2.21 (s, 3H), 2.21 – 2.10 (m, 3H), 1.77 – 1.52 (m, 8H), 1.50 – 1.39 (m, 1H), 1.32 – 1.15 (m, 2H), 0.95 – 0.84 (m, 2H). ^{13}C NMR (126 MHz, DMSO-d_6): δ[ppm] = 175.15, 172.43, 171.35, 170.82, 166.09, 158.10 (pd, $^2J_{C\text{-}F}$ = 34.6 Hz), 156.31 (pd, $^2J_{C\text{-}F}$ = 35.8 Hz), 138.54, 137.77, 137.73, 136.96, 134.57, 131.17, 130.74, 129.78, 129.49, 128.80, 127.19, 127.01, 125.88, 124.67, 120.59, 119.43, 119.42, 117.14, 116.63, 115.02, 66.32, 54.60, 45.08, 43.46, 40.51, 37.26, 36.35, 34.75, 29.36, 29.27, 28.77, 28.49, 28.36, 22.73, 16.70.

Compound **43b** was cyclized as described before. Afterwards, the solvent was removed and the remaining crude Tfa-protected derivative **11c** (structure not shown, HPLC method A: 21.98 min, MS calcd.: 714.34, m/z found: 715.20 $[M+H]^+$) was treated with a mixture of 1 N aq. NaOH, 1,4-dioxane, THF, and acetone as solvent for 1 h at rt to cleave the Tfa-protecting group. The final inhibitor **43** was purified by preparative HPLC (11 mg, 0.013 mmol colorless lyophilized solid, 22 % over two steps, HPLC method A: 18.20 min, purity > 97 %, MS calcd.: 716.37, m/z found: 717.54 $[M+H]^+$).

Inhibitor 44

(1r,4S)-4-(Aminomethyl)-N-((S)-14-methyl-5,8,13,16-tetraoxo-6,9,14,17-tetraaza-7(2,6)-naphthalena-1(1,3),2(1,4)-dibenzenacycloheptadecaphane-4-yl)cyclohexane-1-carboxamide × TFA or H-Txa-Bpa(4'-Me, c[3'-NH)-6-aNap-Gaba-Gly] × TFA

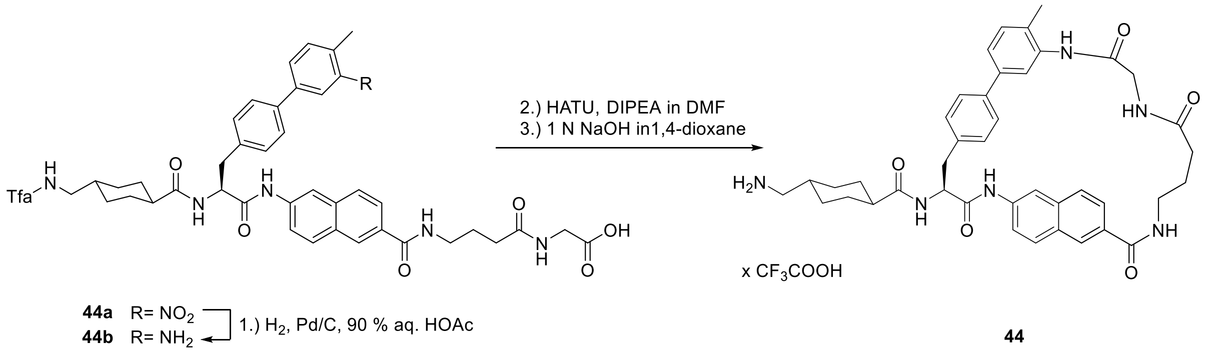

The synthesis was performed as described for inhibitor **33** in the main manuscript. The linear precursor was synthesized on 130 mg 2-CTC resin (0.21 mmol absolute) using Fmoc-Gly-OH,

Fmoc-Gaba-OH, Fmoc-6-aNap-OH **(S11)**, Fmoc-Bpa(4'-Me,3'-NO_2)-OH **(S4)**, and Tfa-Txa-OH **(S1)**. After mild acidic cleavage from resin, the crude compound **44a** (HPLC method A: 29.38 min) was hydrogenated and the amine was purified by preparative HPLC yielding intermediate **44b** (39 mg, 0.05 mmol colorless solid, 23 %, HPLC method A: 20.22 min, purity > 97 %, MS calcd.: 816.35, m/z found: 817.38 $[M+H]^+$). ^{1}H NMR (500 MHz, DMSO-d_6): δ[ppm] = 10.40 (s, 1H), 9.35 (t, ^3J = 5.9 Hz, 1H), 8.57 (t, ^3J = 5.7 Hz, 1H), 8.40 – 8.32 (m, 2H), 8.22 – 8.11 (m, 2H), 7.96 (d, ^3J = 9.1 Hz, 1H), 7.91 – 7.85 (m, 2H), 7.67 (dd, ^3J = 8.9 Hz, ^4J = 2.0 Hz, 1H), 7.50 (d, ^3J = 8.3 Hz, 2H), 7.39 (d, ^3J = 8.3 Hz, 2H), 7.24 – 7.11 (m, 3H), 4.74 (td, ^3J = 8.4 Hz, 5.2 Hz, 1H), 3.75 (d, ^3J = 5.9 Hz, 2H), 3.32 (q, ^3J = 6.7 Hz, 2H), 3.12 (dd, ^2J = 13.8 Hz, ^3J = 4.9 Hz, 1H), 3.03 (t, ^3J = 6.3 Hz, 2H), 2.95 (dd, ^2J = 13.6 Hz, ^3J = 9.5 Hz, 1H), 2.27 – 2.12 (m, 6H), 1.85 – 1.59 (m, 6H), 1.50 – 1.40 (m, 1H), 1.33 – 1.15 (m, 2H), 0.96 – 0.83 (m, 2H). ^{13}C NMR (126 MHz, DMSO-d_6): δ[ppm] = 175.16, 172.30, 171.35, 170.83, 166.21, 158.07 (pd, $^2J_{C\text{-}F}$ = 33.8 Hz), 156.32 (pd, $^2J_{C\text{-}F}$ = 35.6 Hz), 138.50, 137.88, 137.80, 136.88, 134.59, 131.08, 130.68, 129.76, 129.50, 128.80, 127.21, 127.06, 125.87, 124.68, 120.61, 119.73, 117.15, 116.09, 115.02, 114.85, 54.61, 45.08, 43.46, 40.55, 37.26, 36.35, 32.73, 29.36, 29.27, 28.50, 28.36, 25.32, 16.74. One aliphatic signal could not be distinguished from the noise.

Compound **44b** was cyclized as described before. Afterwards, the solvent was removed and the remaining crude Tfa-protected derivative **44c** (structure not shown, HPLC method A: 24.44 min) was treated with a mixture of 1 N aq. NaOH, 1,4-dioxane, THF, and acetone as solvent for 1 h at rt to cleave the Tfa-protecting group. The final inhibitor **44** was purified by preparative HPLC (6 mg, 0.007 mmol colorless lyophilized solid, 15 % over two steps, HPLC method A: 17.80 min, purity > 97 %, MS calcd.: 702.35, m/z found: 703.10 $[M+H]^+$).

Inhibitor 45

(1r,4S)-4-(aminomethyl)-N-((S)-14-methyl-5,8,12,15-tetraoxo-6,9,13,16-tetraaza-7(2,6)-naphthalena-1(1,3),2(1,4)-dibenzenacyclohexadecaphane-4-yl)cyclohexane-1-carboxamide × TFA or H-Txa-Bpa(4'-Me, c[3'-NH)-6-aNap-βAla-Gly] × TFA

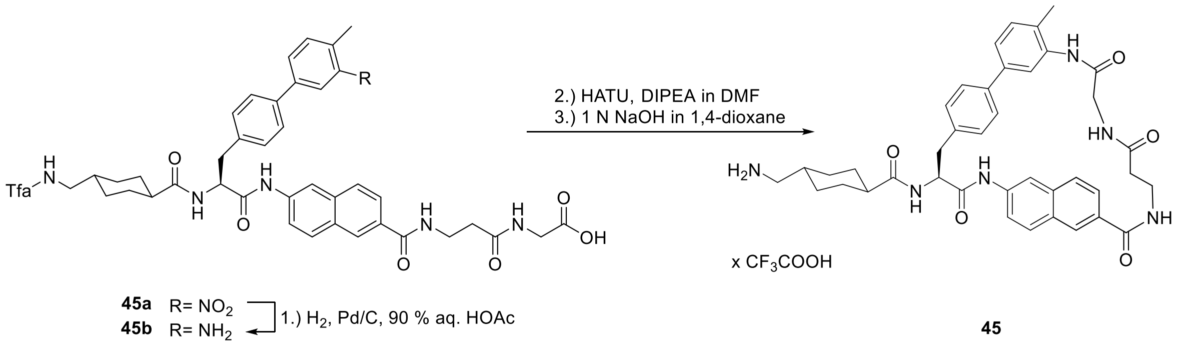

The synthesis was performed as described for inhibitor **33** in the main manuscript. The linear precursor was synthesized on 150 mg 2-CTC resin (0.24 mmol absolute) using Fmoc-Gly-OH, Fmoc-β-Ala-OH, Fmoc-6-aNap-OH **(S10)**, Fmoc-Bpa(4'-Me,3'-NO_2)-OH **(S4)**, and Tfa-Txa-OH **(S1)**. After mild acidic cleavage from resin, the crude compound **45a** (HPLC method A: 29.12 min) was hydrogenated and the amine was purified by preparative HPLC yielding intermediate **45b** (86 mg, 0.11 mmol, 51 %, HPLC method A: 19.94 min, purity > 98 %, MS calcd.: 802.33, m/z found: 803.31 $[M+H]^+$, ^{1}H NMR (500 MHz, DMSO-d_6): δ[ppm]= 10.39 (s, 1H), 9.34 (t, ^3J = 5.8 Hz, 1H), 8.55 (t, ^3J = 5.6 Hz, 1H), 8.35 (s, 1H), 8.33 (d, ^4J = 1.8 Hz, 1H), 8.26 (t, ^3J = 5.9 Hz, 1H), 8.14 (d, ^3J = 8.1 Hz, 1H), 7.96 (d, ^3J = 9.1 Hz, 1H), 7.92 – 7.84 (m, 2H), 7.66 (dd, ^3J = 8.9, ^4J = 2.1 Hz, 1H), 7.50 (d, ^3J = 8.3 Hz, 2H), 7.38 (d, ^3J = 8.3 Hz, 2H), 7.22 – 7.10 (m, 3H), 4.74 (td, 3J = 8.4 Hz, 5.1 Hz, 1H), 3.77 (d, ^3J = 5.9 Hz, 2H), 3.60 – 3.44 (m, 2H), 3.12 (dd, ^2J = 13.9 Hz, ^3J = 4.9 Hz, 1H), 3.03 (t, ^3J = 6.4 Hz, 2H), 2.94 (dd, ^2J = 13.9 Hz, ^3J = 9.6 Hz, 1H), 2.49 – 2.45 (m, 2H), 2.19 (s, 3H), 2.18 – 2.08 (m, 1H), 1.77 – 1.57 (m, 4H), 1.49 – 1.39 (m, 1H), 1.34 – 1.12 (m, 2H), 0.95 – 0.81 (m, 2H), ^{13}C NMR (126 MHz, DMSO-d_6): δ[=ppm]= 175.13, 171.31, 170.83, 170.82, 166.21, 158.30 (pd, $^2J_{C\text{-}F}$ = 35.4 Hz), 156.30 (pd, $^2J_{C\text{-}F}$ = 35.9 Hz), 138.48, 137.91, 137.82, 136.84, 134.61, 131.03, 130.52, 129.74, 129.51, 128.77, 127.22, 127.10, 125.85, 124.60, 120.60, 115.99 (pd, $^1J_{C\text{-}F}$ = 288.3 Hz), 115.00, 70.28, 54.59, 45.07, 43.44, 40.56, 37.26, 36.34, 36.07, 35.07, 29.35, 29.26, 28.49, 28.35, 16.74. In the aromatic region, not all carbon atoms could be clearly distinguished.

Compound **45b** was cyclized as described before. Afterwards, the solvent was removed and the remaining crude Tfa-protected derivative **45c** (structure not shown, HPLC method A: 25.07

min, MS calcd.: 714.34, m/z found: 715.20 [M+H]+) was treated with a mixture of 1 N aq. NaOH, 1,4-dioxane, THF, and acetone as solvent for 1 h at rt to cleave the Tfa-protecting group. The final inhibitor **45** was purified by preparative HPLC (26 mg, 0.032 mmol colorless lyophilized solid, 30 % over two steps, HPLC method A: 16.82 min, purity > 98 %, MS calcd.: 688.34, m/z found: 689.66 $[M+H]^+$).

8. Synthesis of linear reference inhibitors 46 and 47

Inhibitor 46

(4-(2-(4-((S)-3-(3'-amino-4'-methyl-[1,1'-biphenyl]-4-yl)-2-((1r,4S)-4-(aminomethyl)cyclohexane-1-carboxamido)propanamido)phenyl)acetamido)butanoyl)glycine × TFA or H-Txa-Bpa(4'-Me, 3'-NH2)-4-aPhac-Gaba-Gly-OH × TFA

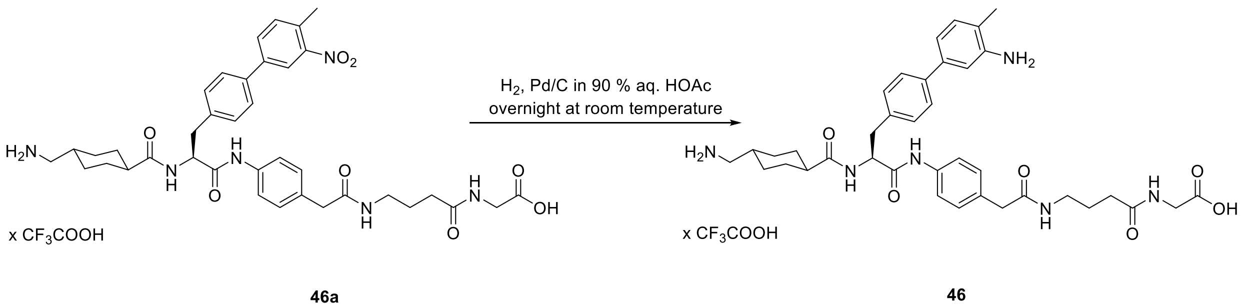

The synthesis was performed as described for the first steps of the synthesis of inhibitor **33** in the main manuscript. The precursor was synthesized on 100 mg 2-CTC resin (0.16 mmol absolute) using Fmoc-Gly-OH, Fmoc-Gaba-OH, Fmoc-4-aPha-OH **(S10)**, Fmoc-Bpa(4'-Me,3'-NO_2)-OH **(S4)**, and Boc-Txa-OH **(S2)**. The peptide was cleaved from the resin using a mixture of TFA/$H_2$0/TIS (95/2.5/2.5, v/v/v) providing the crude compound **46a** (HPLC method A: 19.87 min), which was hydrogenated and purified by preparative HPLC yielding the linear inhibitor **46** (69 mg, 0.086 mmol colorless solid, 54 %, HPLC method A: 12.80 min, purity > 99 %, MS calcd.: 684.36, m/z found: 685.42 $[M+H]^+$).

Inhibitor 47

(1r,4S)-N-((S)-1-((4-(2-((4-((2-amino-2-oxoethyl)amino)-4-oxobutyl)amino)-2-oxoethyl)phenyl)amino)-3-(3'-amino-4'-methyl-[1,1'-biphenyl]-4-yl)-1-oxopropan-2-yl)-4-(aminomethyl)cyclohexane-1-carboxamide × TFA

or H-Txa-Bpa(4'-Me, 3'-NH_2)-4-aPhac-Gaba-Gly-NH_2 × TFA

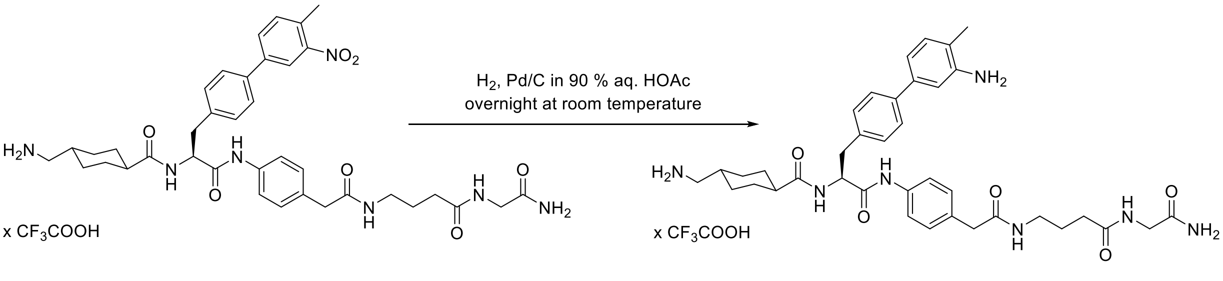

The synthesis was performed as described for the first steps of the synthesis of inhibitor **33** in the main manuscript. The precursor was synthesized on 200 mg Rink-amide resin (loading 0.74 mmol/g, 0.148mmol absolute) using Fmoc-Gly-OH, Fmoc-Gaba-OH, Fmoc-4-aPha-OH **(S10)**, Fmoc-Bpa(4'-Me,3'-NO_2)-OH **(S4)**, and Boc-Txa-OH **(S2)**. The peptide was cleaved from the resin using a mixture of TFA/$H_2$0/TIS (95/2.5/2.5, v/v/v) providing the crude compound **47a** (HPLC method A: 18.53 min), which was hydrogenated and purified by preparative HPLC yielding the linear inhibitor **47** (59 mg, 0.074 mmol colorless solid, 50 %, HPLC method A: 12.32 min, purity > 99 %, MS calcd.: 683.38, m/z found: 684.492 $[M+H]^+$).

9. Structure determination of μ-plasmin mutant in complex with inhibitor 28

Table S1. X-ray crystal structure data collection and refinement statistics (values in parentheses are for the highest resolution shell).

**Complex**	**μ-plasmin Ser195(741)Ala mutant/inhibitor 28**
**PDB ID**	**9AZK**
**Data Collection**	
space group	P1 21 1
NO. mol/au	6
Cell dimensions	
a, b, c (Å)	74.85, 128.88, 79.08
α, β, γ (°)	90.00, 108.06, 90.00
Resolution range (Å)	47.76 – 2.1
R_{merge}/R_{pim}	0.221 (1.599)/0.093 (0.682)
Mean *I*/sigma(*I*)	7.4 (1.5)
Completeness (%)	100 (100)
Multiplicity	6.5 (6.4)
**Refinement**	
No. reflections (total/R_{free})	82777/4059
R_{work}/R_{free}	0.2370/0.2549
No. of non-hydrogen atoms	12266
macromolecules	11363
ligands	570
solvent	609
Average B-factor	40.26
macromolecules	40.91
ligands	21.68
solvent	37.07
r.m.s deviations	
Bond lengths (Å)	0.013
Bond angles (°)	1.55
MolProbity analysis	
Ramachandran favored	95.13%
Ramachandran allowed	4.38%
Ramachandran outliers	0.49%
Clash score	12.49

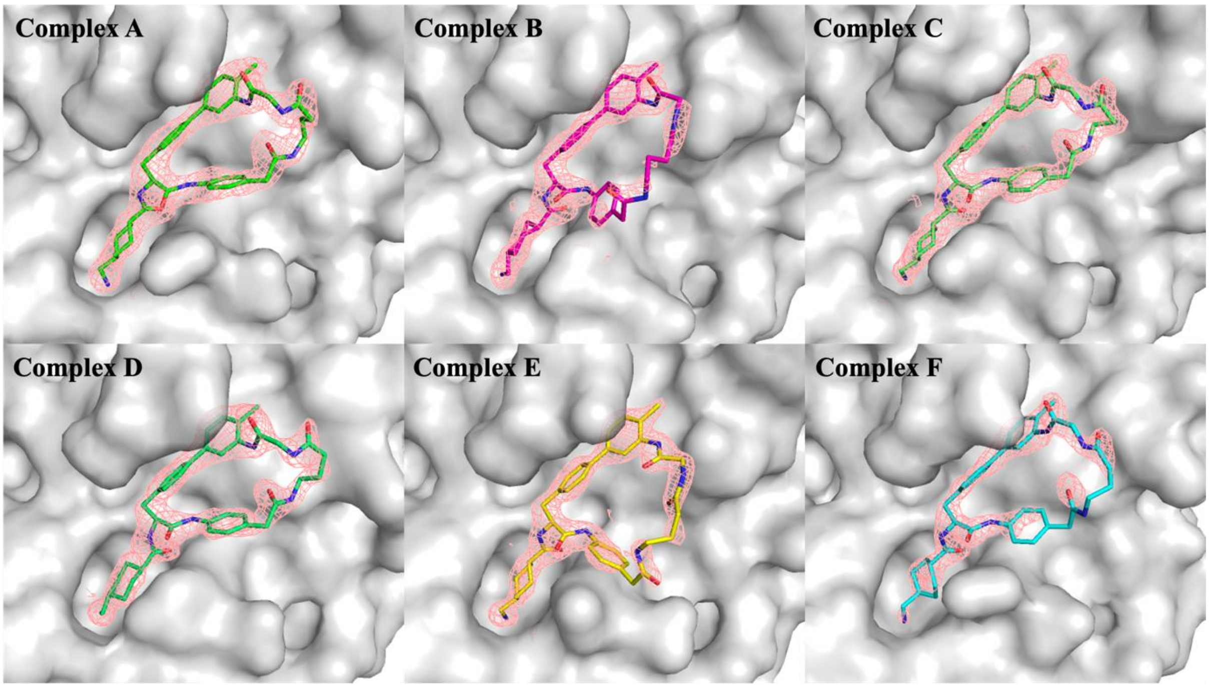

Figure S1. In the co-crystal structure of μ-plasmin Ser195(741)Ala mutant/inhibitor **28**, there are six molecules per asymmetric unit (Complex A-F, as shown). The μ-plasmin is shown as grey surfaces and inhibitor **28** as sticks and colored. The 2Fo-Fc electron density maps (salmon color) of the inhibitors are contoured at 1.5 sigma.

10. Abbreviations

6-Aca, 6-aminocaproic acid; 5-Ava, 5-aminovaleric acid; Boc, tert-butyloxycarbonyl; Cbz, benzyloxycarbonyl; DIPEA, N,N-diisopropylethylamine; DCM, dichloromethane; DMF, N,N-dimethylformamide; EtOAc, ethyl acetate; HATU, 2-(7-Aza-1H-benzotriazol-1-yl)-1,1,3,3-tetramethyluronium-hexafluorophosphat; HBTU, 2-(1H-Benzotriazole-1-yl)-1,1,3,3-tetramethyluronium hexafluorophosphate; hPhe, homophenylalanine; MS, mass spectrometry; NMM, 4-methylmorpholine; Phe(4-NO_2), p-nitrophenylalanine; TFA, trifluoroacetic acid; Tfa, trifluoroacetyl; THF, tetrahydrofurane.
